# USP8 controls proteostasis pathways in B cells and multiple myeloma

**DOI:** 10.1038/s41375-026-03086-y

**Published:** 2026-08-11

**Authors:** Almut Dufner, Fabien Thery, Gianni Monaco, Jelena Lazarevic, Oliver Gorka, Nina Chevalier, Marta Campos Alonso, Maximilian Frosch, Gerbrand J. van der Heden van Noort, Kira Allmeroth, Marco Prinz, Olaf Groß, Huib Ovaa, Paul P. Geurink, Wolfgang W. Schamel, Martin S. Denzel, Vigo Heissmeyer, Benedikt Jacobs, Mirle Schemionek, Francis Impens, Heiko Bruns, Klaus-Peter Knobeloch

**Affiliations:** 1 Institute of Neuropathology, Medical Center–University of Freiburg/Medical Faculty - University of Freiburg, Freiburg, Germany; 2 VIB-UGent Center for Medical Biotechnology, Gent, Belgium; 3 Department of Biomolecular Medicine, Gent, Belgium; 4 Institute for Transfusion Medicine and Gene Therapy, Medical Center - University of Freiburg/Medical Faculty - University of Freiburg, Freiburg, Germany; 5 Department of Hematology, Oncology, Hemostaseology and Stem Cell Transplantation University Hospital RWTH Aachen, Aachen, Germany; 6 Department of Rheumatology and Clinical Immunology, Medical Center – University of Freiburg/Medical Faculty - University of Freiburg, Freiburg, Germany; 7 Department of Cell and Chemical Biology, Leiden University Medical Center, Leiden, The Netherlands; 8 Max Planck Institute for Biology of Ageing, Cologne, Germany; 9 Signalling Research Centres BIOSS and CIBSS, University of Freiburg, Freiburg, Germany; 10 Center for Basics in NeuroModulation (NeuroModulBasics), Medical Center – University of Freiburg/Medical Faculty - University of Freiburg, Freiburg, Germany; 11 Faculty of Biology, Albert-Ludwigs-University of Freiburg, Freiburg, Germany; 12 Center of Chronic Immunodeficiency CCI, Medical Center – University of Freiburg/Medical Faculty - University of Freiburg, Freiburg, Germany; 13 CECAD - Cluster of Excellence, University of Cologne, Cologne, Germany; 14 Center for Molecular Medicine Cologne (CMMC), University of Cologne, Cologne, Germany; 15 Institute for Immunology, Biomedical Center, Ludwig-Maximilians-Universität München, Planegg-Martinsried, Germany; 16 Research Unit Molecular Immune Regulation, Helmholtz Zentrum München, Munich, Germany; 17 Department of Internal Medicine 5, Hematology and Oncology, Friedrich Alexander University Erlangen-Nuremberg (FAU) and Universitätsklinikum Erlangen, Erlangen, Germany; 18 Institute for Functional Gene Analytics (IFGA), Bonn-Rhein-Sieg University of Applied Sciences, Rheinbach, Germany; 19 VIB Proteomics Core, Gent, Belgium

**Keywords:** Myeloma, Preclinical research

## Abstract

Ubiquitin-specific protease 8 (USP8) plays a pivotal role in the regulation of endosomal and lysosomal trafficking and is critically involved in the pathogenesis of various tumor entities. USP8 represents a vulnerability gene in multiple myeloma (MM), suggesting a functional role in the B- and plasma cell compartment. Here we analyzed mice with stage-specific *Usp8* deletion during B-cell development and investigated its role in patient-derived MM cells that are sensitive or resistant to the proteasome inhibitor Bortezomib (BTZ) using USP8 depletion and treatment with DUB-IN-2, a reported USP8 inhibitor. *Usp8* depletion in *Usp8*^f/f^C*d19*-Cre mice affected B-cell survival and development favoring immature, innate-like B cells, and germinal center and plasma cells, while also elevating immune-responses and causing Roquin depletion. Cells expressing catalytically inactive USP8 accumulated proteins modified with mixed ubiquitin/NEDD8 chains indicative of proteotoxic stress, which we identified as preferred USP8 substrates. In MM cells, USP8 knockdown reduced survival via lysosomal dysfunction. In contrast, DUB-IN-2 induced an enhanced ER stress response to treatment with BTZ questioning DUB-IN-2 function as a USP8 inhibitor, as confirmed by biochemical analysis. Thus, our results highlight the therapeutic potential of targeting USP8 and identify the combination of DUB-IN-2 and BTZ as a novel strategy for treating BTZ-resistant MM.

**Figure:**
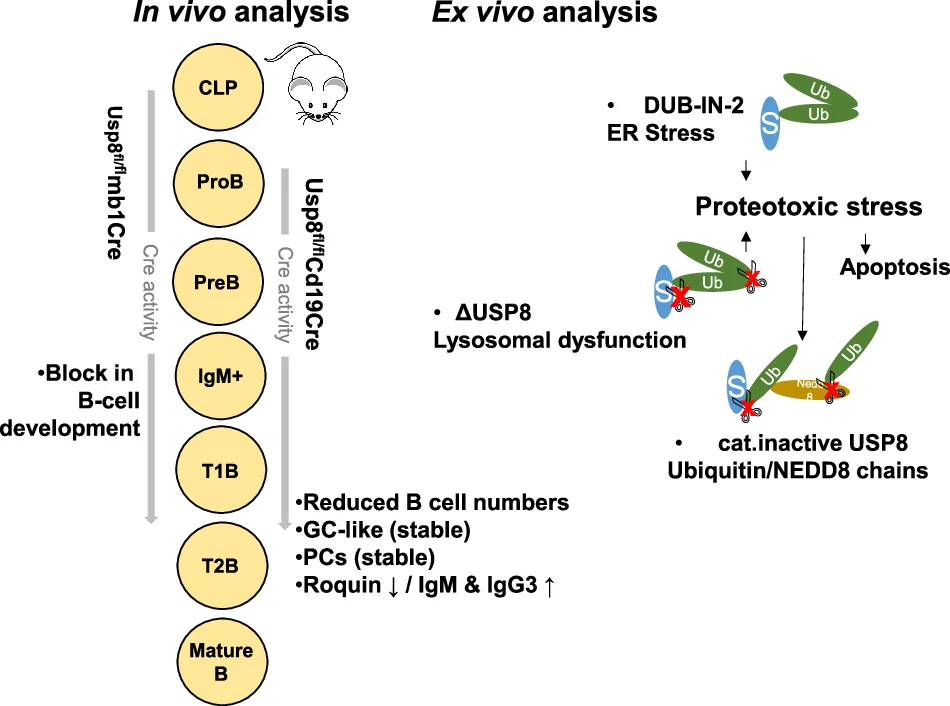

## Introduction

Targeting the ubiquitin proteasome system (UPS) represents an effective strategy in B-cell malignancies such as multiple myeloma (MM) [1]. Although proteasome inhibition by bortezomib (BTZ, Velcade®) or Carfilzomib (CFZ, Kyprolis®) improve patient outcome, relapse is common. More than 90 deubiquitinating enzymes (DUBs), including ~50 USPs, counteract ubiquitylation [2]. USP8 has a multi-domain structure and regulates endosomal sorting and transmembrane protein stability through interactions with endosomal sorting complexes required for transport (ESCRT) components, including STAM2 and charged multivesicular body (CHMP) proteins [3]. USP8’s C-terminal catalytic domain is inhibited by phosphorylation-dependent 14-3-3 binding to its adjacent motif [4, 5]. Somatic mutations affecting the 14-3-3 BM cause Cushing’s disease (CD) leading to EGFR stabilization in ACTH-secreting pituitary adenomas [6, 7]. USP8 was also associated with autophagy and mitophagy regulation [3, 8, 9], and with the progression of many types of cancer including effects on the tumor microenvironment [10–14]. Multiple studies on USP8 in cancer have used DUB-IN-2, a reported USP8 inhibitor [10–12, 15–17], but its mechanism of action, specificity and kinetic parameters remain unclear [18, 19].

In T cells, USP8 interacts with GRB2-related adaptor protein downstream of Shc (GADS) and controls stability of the ESCRT component CHMP5 to stabilize the anti-apoptotic Bcl2 protein as a prerequisite for the positive selection of thymocytes [20]. T-cell-specific USP8 deletion impairs T-cell expansion and leads to spontaneous colitis with dysfunctional regulatory T cells [21].

Ubiquitylation and deubiquitylation also regulate B-cell activation, proliferation, apoptosis, and their differentiation and function in innate and humoral immune responses [22]. USP8 was identified as a stimulation-dependent component of the B-cell receptor signalosome [23]. Moreover, an RNAi lethality screen identified *Usp8* as a key gene with increased vulnerability in MM compared to non-MM cells [24]. MM cells, which represent malignant plasma cells, have high protein synthesis rates, depend on UPS-mediated turnover, and are highly sensitive to disruptions in protein homeostasis. Inhibiting USPs like USP7, USP9X, and USP14 induces apoptosis in MM cells, underscoring the therapeutic potential of USP inhibitors [25–27]. Despite links to B-cell signaling and MM survival, USP8’s role in B-cell development, immunity, and MM pathogenesis remains unclear.

Here, we investigate the role of USP8 in B-cell development and multiple myeloma (MM), including proteasome inhibitor-resistant disease. Using stage-specific USP8 deletion models, we uncover essential functions of USP8 in early B-cell development and immune homeostasis. Mechanistically, USP8 regulates proteotoxic stress responses linked to ubiquitin signaling. While DUB-IN-2 [18] resensitizes BTZ-resistant MM cells via ER stress, USP8 knockdown reduces survival independently of BTZ by inducing lysosomal dysfunction. Our findings highlight USP8 inhibition and DUB-IN-2/BTZ combination as potential strategies against BTZ-resistant MM, with DUB-IN-2 acting via USP8-independent effects.

## Methods

Supplementary Materials and Methods are provided in the Supplementary Data file on the Leukemia website.

### Cell preparation and flow cytometry

Spleens, lymph nodes, and thymi were processed into single-cell suspensions in FACS buffer (PBS supplemented with 1–2% FCS). Bone marrow cells were obtained from tibias and femurs by flushing with FACS buffer. Peritoneal cavity cells were harvested post-euthanasia by i.p. injection of FACS buffer followed by gentle massage. Splenocytes and BM cells were depleted of red blood cells using RBC lysis buffer (Sigma or BioLegend). Cell suspensions were incubated with mouse Fc Block (BD or BioLegend) prior to staining. B cells were isolated from spleen using biotinylated anti-CD19 antibody (clone ID3, BD Pharmingen) or the BD IMag^TM^ Biotinylated Mouse B Lymphocyte Enrichment Cocktail in combination with Streptavidin Particles Plus. T cells were isolated using the BD IMag^TM^ Biotinylated Mouse CD4 T lymphocyte enrichment Cocktail with Streptavidin Particles Plus. DAPI or Zombie UV fixable viability dye (Biolegend) was used for live/dead discrimination. Surface and intracellular marker expression was analyzed using flow cytometers (Becton Dickinson FACSCalibur, FACSCanto II, or LSRFortessa SORP) and FlowJo software. Annexin V/PI staining was performed using the eBioscience Annexin V apoptosis detection kit (APC).

### Cell culture, proliferation, activation, and viability

Purified B cells (5 × 10^4^/well) were stimulated with anti-IgM F(ab’)₂ (5 µg ml^−1^, 115-006-020, Jackson Immuno Research) ± anti-CD40 (5 µg ml^−1^, 553787, BD Pharmingen), 100 ng/ml MPL-A (Invivogen) or 100 nM CpG (ODN 1668, Invivogen). Proliferation was measured after 36 h using a BrDU ELISA (Roche). For short-term stimulation, B cells were starved for 2 h in RPMI 1640 with 0.2% bovine serum albumin (no FCS), preincubated with anti-IgM (10 µg ml^−1^) for 30 min on ice, and stimulated at 37 °C. T cells were starved for 2 h in RPMI 1640 without FCS, pre-incubated with soluble anti-mouse CD3ε (10 µg ml^−1^, 145-2C11, eBioscience) and anti-mouse CD28 (2 µg ml^−1^, 37.51, eBioscience) for 30 min on ice, and stimulated at 37 °C with anti-hamster IgG1 (5 µg ml^−1^, G94-56, BD Pharmingen). Ex vivo B cell expansion was performed using murine IL-4 (100 u ml^−1^, Peprotech) and anti-mouse CD40 (5 µg ml^−1^, HM40-3, Biolegend), or anti-IgM F(ab’)₂ fragment (5 µg ml^−1^) plus anti-CD40 (2.5 µg ml^−1^). OHT (1 µM, Sigma) was added 24 h after isolation. DUB-IN-2 (25 µM, MedChemExpress), BTZ (5 to 25 nM, Selleckchem), CFZ (3 nM, MedChemExpress), and OTUB1/USP8 inhibitor 61 (Aobious) were added as indicated. Viability was assessed using the XTT cell proliferation kit II (Roche Diagnostics) and normalized to controls.

### Cell lines, expression constructs, transfection, and inhibitor treatment

NP-specific mIgM-BCR–expressing transfectant K46Lμm cells derived from the Balb C mouse B-cell lymphoma line K46 [28, 29] were obtained from W. Schamel. The human MM cell lines KMS-18 and KMS-27, as well as their BTZ resistant derivatives (T21A and A49V) expressing mutant PSMB5, have been described previously [30]. Cell lines were routinely tested to avoid mycoplasma contamination. Human and mouse USP8, C-terminally FLAG-tagged, were expressed using pFLAG-CMV5a and the retroviral expression vector MIEG3, respectively. Retroviral particles were generated in Platinum-E packaging cells using standard protocols. Mammalian expression vectors encoding HA-NEDD8 and HA-SENP8 were obtained from Addgene. Point mutations were introduced by site-directed mutagenesis using the QuikChange system (Stratagene). HEK293T cells were transfected with FuGENE HD (Promega) and harvested 36 h post transfection. MLN4924 (NAE inhibitor; LKT Laboratories), MLN7243 (UBA1 inhibitor; MedChemExpress), folimycin (MedChemExpress), bortezomib (BTZ; Selleckchem), and DUB-IN-2 (MedChemExpress) were used as indicated. ON-TARGETplus SMARTpool siRNA directed against human USP8 and CHOP, individual siRNAs directed against USP8 (Target Sequence siRNA_1: GGCAAGCCAUUUAAGAUUA; siRNA_2: CCACUAGCAUCCACAAGUA), and a non-targeting (NT) control pool were purchased from Dharmacon. SiRNA transfections were perfomed using Lipofectamine® RNAiMAX. For rescue experiments, the siRNA_2 target sequence in USP8 was mutated to CCCCUAGCUUCCACAAGUA.

## Results

### USP8 is essential for B-cell development

USP8 is critical for T-cell homeostasis where GADS acts as a binding partner [21]. However, B lymphocytes express only marginal levels of GADS (Fig. S1A), suggesting a different mechanism. To study USP8 function in B cells, we generated *Usp8*^f/f^*Cd19*-Cre mice, which delete Usp8 from the pre-B-cell stage, and compared them with *Usp8*^f/f^ controls (Fig. S1B). Residual USP8 protein in splenic B cells indicates a potential survival advantage of cells with incomplete deletion (Fig. S1C).

**Fig. 1:**
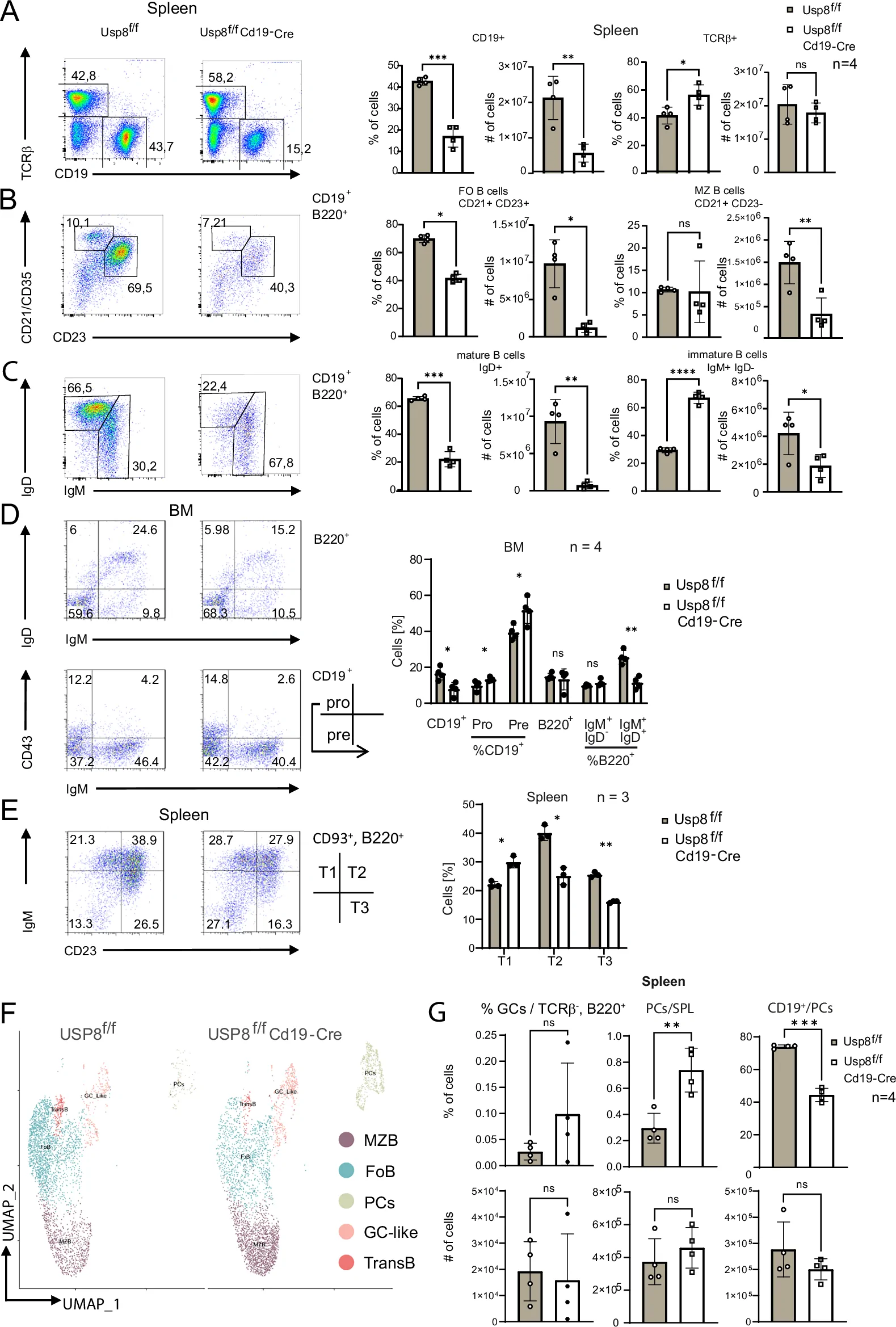
Defect in B-cell development in *Usp8*^f/f^*Cd19*-Cre mice. **A**–**C** Flow cytometry analysis of B-cell subsets in spleen of *Usp8*^f/f^ and *Usp8*^f/f^*Cd19*-Cre mice (*n* = 4). Pre-gating is indicated at the top right of the FACS plots. Percentages and numbers of B-cell subsets are shown in the right panels. Unpaired 2-tailed t-test, mean + /- sd. **D** Flow cytometry analysis of *Usp8*^f/f^ and *Usp8*^f/f^*Cd19*-Cre bone marrow (BM) (*n* = 4). Paired 2-tailed t-test, mean + /- sd. **E** Flow cytometry analysis of splenic transitional *Usp8*^f/f^ and *Usp8*^f/f^*Cd19*-Cre B-cell subsets (*n* = 3). Paired, 2-tailed t-test, mean + /-sd. **F** Uniform Manifold Approximation and Projection (UMAP) of B cells enriched from spleens of *Usp8*^f/f^ and *Usp8*^f/f^*Cd19*-Cre spleens. **G** Flow cytometry analysis of splenic *Usp8*^f/f^ and *Usp8*^f/f^*Cd19*-Cre germinal center (GC; GL7^hi^, Fas^hi^, TCR-β^-^, B220^+^) B cells and CD138^hi^ PCs, and CD19 expression on PCs (*n* = 4). Unpaired 2-tailed t-test, mean + /- sd. MZB marginal zone B cells, FoB follicular B cells, Pro pro-B cells, Pre pre-B cells, TransB Transitional B cells, T1, T2, T3 Transitional T1, T2, T3 B cells, BM bone marrow, PCs plasma cells, GC-like germinal center-like. Paired analysis was used, if sex-matched littermates were derived from n different litters. Source data are provided as source data file.

In contrast to T cells, *Usp8*^f/f^C*d19*-Cre mice exhibited reduced frequencies and numbers of splenic CD19^+^ B cells (Fig. 1A), with a stronger reduction in follicular than in marginal zone B cells (Fig. 1B), consistent with impaired development of mature (IgD^+^) splenic B cells (Fig. 1C). Similarly, percentages of CD19^+^ and mature IgM^+^IgD^+^ BM B cells were reduced, whereas early BM B cells were unaffected (Fig. 1 D). Transitional B-cell development was impaired from the T2 stage onward (Fig. 1E). These findings were confirmed using *Usp8*^+/+^*Cd19*-Cre controls (Fig. S1D). B1 subsets showed a trend toward reduced numbers but inreased frequencies within the IgM^+^ compartment (Fig. S1E). Splenic architecture reflected these defects, with reduced B220+ areas and primary follicles (FO), while T-cell zones remained unaffected (Fig. S1F). USP8-deficient B cells displayed impaired proliferation upon BCR or TLR stimulation, paralleled by reduced Ki67+ cells in vivo (Fig. S1G, H). To assess earlier developmental requirements, we generated *Usp8*^f/f^*mb1*-Cre mice, which delete Usp8 from the pro B-cell stage onward [31]. These mice exhibited a developmental block at the pre-B-cell stage, resulting in the absence of peripheral B cells (Fig. S1I).

Together, these data demonstrate that USP8 is essential for B-cell development at multiple stages.

### Elevated Ig responses and robust PC numbers in *Usp8*^f/f^*Cd19*-Cre mice

To further characterize consequences of USP8 depletion on B-cell development, we performed single cell RNA-seq analysis of splenic B cells from *Usp8*^f/f^ and *Usp8*^f/f^*Cd19*-Cre mice. Clustering confirmed reduced follicular and transitional B cells, alongside relatively increased GC-like B cells and PCs (Figs. 1F, S2A–C). Total GC B-cell and PC numbers remained similar, but CD19+ PCs were underrepresented, suggesting a survival disadvantage of cells with efficient *Cd19*-Cre-mediated *Usp8* deletion (Fig. 1G). Differential gene expression analysis showed increased IL-9R in FoB and MZB, higher expression of specific Ig variable regions in PCs, and reduced ZFP318, a transcriptional regulator of IgD expression [32], in GC-like B cells and FoB of *Usp8*^f/f^*Cd19*-Cre mice (Fig. S2D).

**Fig. 2:**
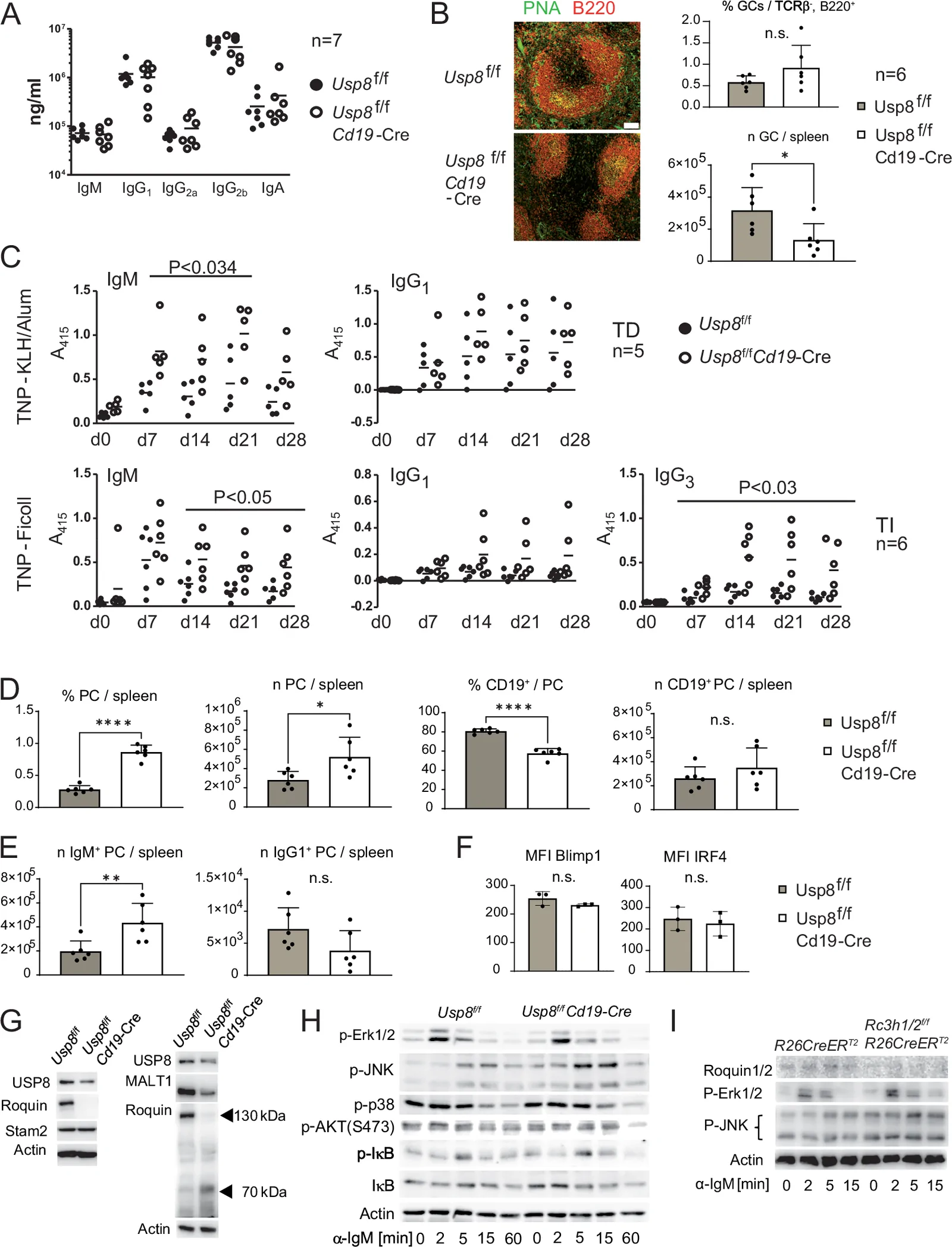
*Usp8*^f/f^*Cd19*-Cre mice exhibit enhanced PC numbers and Ig responses upon T cell-dependent and T cell-independent immunization. **A** Normal basal immunoglobulin titers in *Usp8*^f/f^*Cd19*-Cre mice (*n* = 7). **B** Control and *Usp8*^f/f^*Cd19*-Cre mice were i.p. injected with 50 μg TNP-KLH precipitated in alum and GC B cells were visualized by fluorescence imaging of frozen sections (scale bar, 100 µm) and quantified by FACS analysis (GL7^hi^, Fas^hi^, TCR-β^-^, B220^+^) at day 14 post injection (*n* = 6, unpaired 2-tailed t-test, mean + /-sd). **C** Detection of TNP-specific Ig present in the serum of *Usp8*^*f/f*^ and *Usp8*^f/f^*Cd19*-Cre mice that have been i.p. injected with 50 μg TNP-KLH precipitated in alum (TD response, *n* = 5) or with 25 μg TNP-Ficoll (TI response, *n* = 6) by ELISA. Significant differences are summarized collectively. Unpaired 2-tailed t-test, mean + /-sd. *Usp8*^*f/f*^ and *Usp8*^f/f^*Cd19*-Cre mice were treated as in (**B**) and spleen single cell suspensions were analyzed by FACS to determine frequencies of CD138^hi^ PCs and CD19 expression on PCs (*n* = 6) (**D**), frequencies of IgM^+^ and IgG1^+^ PCs (*n* = 6) (**E**), as well as the expression of Blimp1 and IRF4 in PCs (*n* = 3) (**F**). Unpaired 2-tailed t-test, mean + /-sd. **G** Splenic *Usp8*^f/f^ and *Usp8*^f/f^*Cd19*-Cre B cells were enriched and analyzed by Western blotting (WB). Results are representative of at least 2 independent experiments. **H** Splenic *Usp8*^f/f^ and *Usp8*^f/f^*Cd19*-Cre B cells were starved for 2 h, stimulated for the indicated times with anti-IgM F(ab’)₂ fragment antibody, and analyzed by WB. Results are representative of at least 2 independent experiments. **I** Upon oral gavage of tamoxifen, Roquin1/2 depleted *Rc3h1/2 *^f/f^ *Rosa26-*CreERT2 B cells and *Rosa26-*CreERT2 control cells were enriched from spleens, stimulated as in (**H**), and analyzed by WB (*n* = 3). Source data are provided as source data file.

Basal serum Igs were unchanged (Fig. 2A). Although total GC B-cell numbers were reduced in spleens of TNP-KLH-immunized *Usp8*^f/f^*Cd19*-Cre mice, their frequencies among B cells were unchanged (Fig. 2B). After immunization, IgM and IgG3 levels and splenic PC numbers were increased (Fig. 2C). However, as in unimmunized mice, CD19^+^ PCs were underrepresented within the PC compartment (Fig. 2D). Consistent with elevated IgM responses, *Usp8*^f/f^*Cd19*-Cre mice had more IgM-positive, but not IgG1 positive PCs per spleen (Fig. 2E). Key transcription factors remained unchanged (Fig. 2F). T-cell subsets and basal cytokines were unaffected (Fig. S2E, F).

Roquin proteins (Roquin-1 and -2) are essential for shaping T and B cell-dependent immune responses [33]. We therefore hypothesized that Roquin function might be impaired in *Usp8*^f/f^*Cd19*-Cre B cells. Indeed, Roquin-1/2 levels, but not STAM2, were reduced in USP8-depleted B cells, whereas short-term USP8 deletion ex vivo did not alter Roquin or CHMP5, indicating indirect regulation (Figs. 2G, S3A). Roquin cleavage is mediated by Malt1, whose protease activity is enhanced by mono-ubiquitylation at K644 or excess free ubiquitin [34, 35]. MALT1 ubiquitination was not detected, but gene expression analyses showed coordinated regulation of Usp8, Roquin, and Malt1 (Fig. S3B).

Mechanistically, USP8-depleted B cells showed enhanced JNK activation and Ca2+ mobilization upon BCR stimulation, while Erk1/2, p38, AKT, IκB, and global tyrosine phosphorylation were unaffected (Figs. 2H, S3C, D). Ex vivo USP8 deletion did not reproduce these effects, whereas in vivo Roquin1/2 loss recapitulated increased JNK activation, implicating Roquin loss in this phenotype (Fig. 2I, S3E–G). Pathway analysis further suggested altered Rho-GTPase signaling and stress responses in USP8-deficient B-cell subsets (Fig. S3H, Table S1).

In summary, USP8 deficiency skews the splenic B-cell compartment toward GC-like B cells, PCs, immature, and innate-like B cells with a specific underrepresentation of CD19+ PCs, and enhances Ig and signaling responses, likely driven by indirect effects on Roquin and downstream pathways.

### USP8 limits proteotoxic stress and cleaves ubiquitin/NEDD8 mixed chains

**Fig. 3:**
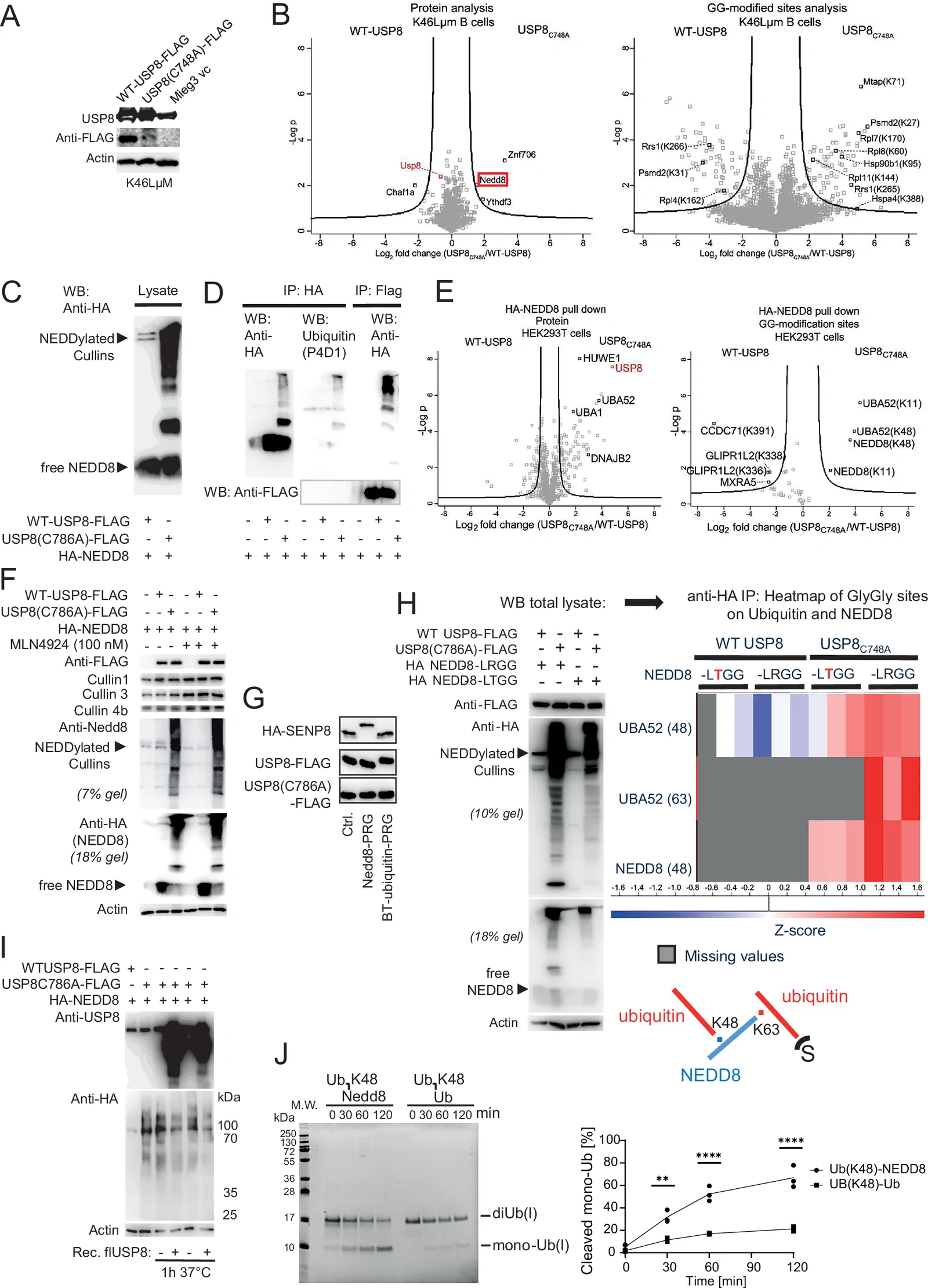
USP8 regulates mixed ubiquitin/NEDD8 chain formation indicating proteotoxic stress. **A**, **B** NEDD8 levels and ribosomal protein modifications are elevated in B cells expressing USP8(C748A)-FLAG. **A** WT-USP8-FLAG and USP8(C748A)-FLAG expression in K46Lµm B cells analyzed by WB. **B** Volcano plots of USP8(C748A):WT-USP8 diGly proteomics experiments. Differentially expressed proteins (shotgun analysis) and regulated diGly sites in proteins derived from K46Lµm B cells (*n* = 3). Lysine positions of diGly sites are included in parentheses. **C**–**E** HEK293T cells transfected with WT-USP8-FLAG or USP8(C786A)-FLAG in the presence of HA-NEDD8. **C** Lysates analyzed by WB. **D** Anti-HA and anti-FLAG pulldowns analyzed by WB. **E** Anti-HA pulldowns analyzed by MS (*n* = 3) to quantify pulled down proteins and GlyGly-K sites. The lysine position of the identified di-glycine site is included in parentheses. **F** HEK293T cells transfected as in (**C**). 24 h prior to lysis cells were treated with MLN4924 (100 nM) as indicated and lysates were analyzed by WB. **G** HEK293T cells transfected with HA-SENP8, WT-USP8-FLAG or USP8(C786A)-FLAG. Lysates were incubated with buffer (ctr.), NEDD8-propargylamide (NEDD8-PRG), or ubiquitin-PRG and analyzed by WB. **H** HEK293T cells transfected with WT-USP8-FLAG or USP8(C786A)-FLAG in the presence of HA-NEDD8(LRGG) or HA-NEDD8(LTGG). Lysates were analyzed by WB (left panels). Anti-HA pulldowns were analyzed by MS (3 replicates/condition) and alterations of ubiquitin (UBA52) and NEDD8 diGly sites are shown in the heat map. Hybrid linkages between both modifiers are reconstructed in the scheme below. **I** Protein lysates from 293T cells transfected with USP8(C786A)-FLAG and HA-NEDD8 were incubated with recombinant USP8 and analyzed by WB. **J** DUB cleavage assays were performed with recombinant human full-length USP8 and synthetic K48-ubiquitinated-NEDD8 (*n* = 3) or K48-diubiquitin (*n* = 2) for the times indicated. Cleavage products were visualized after protein electrophoresis and protein staining of the gel. Statistical analysis after quantification utilized two-tailed unpaired t-test. Source data are provided as source data file.

To further investigate USP8’s role in B cell signaling, survival, and homeostasis, we stably expressed FLAG-tagged mouse wild-type (WT) USP8 and catalytically inactive USP8(C748A) in the murine K46Lµm B-cell lymphoma line (expressing NP-specific mIgM-BCR) [28, 29] (Fig. 3A) and analyzed the proteome and ubiquitin/ubiquitin-like modifications using the diGly enrichment (Ubiscan®) [36]. Overexpression of catalytically inactive USPs can trap their substrates [37]. Unexpectedly, NEDD8 levels were elevated in B cells expressing USP8(C748A) (Fig. 3B, left panel). NEDD8, the ubiquitin-like (ubl) modifier most similar to ubiquitin [38], carried multiple ubiquitin/ubl modified sites itself, including K11, K22, K33, K48, K54, and K60 (deposited in PRIDE database, identifier PXD024395; for accession details please see data availability section). These data suggest that USP8(C748A) increases levels of free or conjugated NEDD8, and that NEDD8 itself undergoes modification by either ubiquitylation or NEDDylation, which cannot be distinguished using diGly enrichment. Enhanced ubiquitin/ubl-modification was also observed on ribosomal proteins Rpl7, Rpl8, Rpl11, and the ribosome biogenesis regulator 1 (Rrs1) (Fig. 3B, right panel).

NEDDylation, like ubiquitin conjugation, is a sequential process mediated by the NEDD8 activating enzyme (NAE, a NAE1/UBA3 heterodimer), the conjugating enzymes UBE2M (UBC12) and UBE2F, and various E3 ligases. Its main targets are cullin proteins, whose mono-NEDDylation activates cullin-RING E3 ubiquitin ligases (CRLs). Under conditions of proteasome inhibition, the altered free ubiquitin-to-NEDD8 ratio triggers atypical conjugation of NEDD8 by the ubiquitin activating enzyme E1 (UBE1/UBA1) and the E3 ligase HUWE1, forming mixed ubiquitin/NEDD8 chains [39–44] that help counter proteotoxic stress [44]. Ribosomal substrates form nuclear aggregates when modified by ubiquitin/NEDD8 mixed chains shielding the nuclear UPS from excess proteins as a hallmark of proteotoxic stress [44].

Similar to atypical NEDDylation during proteotoxic stress, co-expressing HA-NEDD8 with human USP8(C786A)-FLAG in HEK293T cells strongly enhanced overall NEDDylation compared to WT USP8 (Fig. 3C). HA-NEDD8 pulldown contained ubiquitylated proteins, indicating mixed ubiquitin/NEDD8 chains (Fig. 3D), while USP8(C786A)-FLAG pulldowns captured NEDDylated substrates, but not free NEDD8, indicating that catalytically inactive USP8 traps them via ubiquitin in these mixed chains (Fig. 3D). Mass spectrometry (MS) of HA-NEDD8 pulldowns revealed increased accumulation of inactive USP8 versus WT with no ubl-modifications detected on USP8 itself (Fig. 3E, PRIDE PXD024755). Strikingly, UBA52 (ubiquitin), as well as UBE1 (UBA1) and HUWE1 were strongly enriched in the HA-NEDD8 pulldowns with USP8(C786A)-FLAG. Quantification of K-ε-GG modified peptides revealed K11- and K48-linked ubiquitin/NEDD8 modifications, indicating chains form mainly via these linkages (Fig. 3E). Mutating HA-NEDD8 K11/K48 to arginine reduced substrate NEDDylation and ubiquitylation (Fig. S4A). Mixed chains were further supported by the absence of K48/K63-specific ubiquitylation and significant sumoylation (Fig. S4B). NEDD8 (K22, K33) and ubiquitin (K63) modifications were detected only with USP8(C786A)-FLAG (PRIDE PXD024755). Consistent with non-canonical NEDDylation, the NAE1 inhibitor MLN4924 blocked Cullin NEDDylation, but not NEDDylation induced by USP8(C786A)-FLAG overexpression (Fig. 3F). In line with the lack of deNEDDylation activity of USP8, USP8 formed a covalent adduct with ubiquitin–propargylamide (PRG) [45], but not NEDD8-PRG, which selectively targeted the deNEDDylase SENP8 (Fig. 3G). These findings suggest that inactive USP8 induces proteotoxic stress, and that USP8 cleaves ubiquitin within mixed ubiquitin/NEDD8 chains.

Identification of non-cullin NEDD8 substrates has been limited by the lack of MS approaches that distinguish between ubiquitylation and NEDDylation. To confirm ubiquitin/NEDD8 hybrid chain formation (versus parallel chain formation or non-covalent interactions) and map reciprocal modification sites, we co-expressed USP8(C786A)-FLAG (or WT-USP8-FLAG) with HA-tagged WT NEDD8 or a C-terminal mutant (LRGG > LTGG) that remains conjugation-competent, but cannot generate the K-ε-GG remnants upon trypsin digestion [46] (Fig. 3H). MS analysis of HA-pulldowns detected K48 modifications on both, WT-NEDD8 and NEDD8(LTGG), identifying NEDD8(K48) as a ubiquitylation site. Conversely, K63 modifications occurred only in the presence of WT-NEDD8, indicating NEDDylation of ubiquitin at K63. Consistent with USP8-mediated cleavage of mixed chains, recombinant USP8 reduced substrate modifications (Fig. 3I). In vitro deubiquitination assays further showed that USP8 cleaves K48-linked ubiquitin/NEDD8 branches more efficiently than K48-diubiquitin (Fig. 3J).

These data indicate that loss of USP8 activity promotes proteotoxic stress and that USP8 directly cleaves mixed ubiquitin/NEDD8 chains.

### USP8 controls proteostasis and survival of primary B cells

**Fig. 4:**
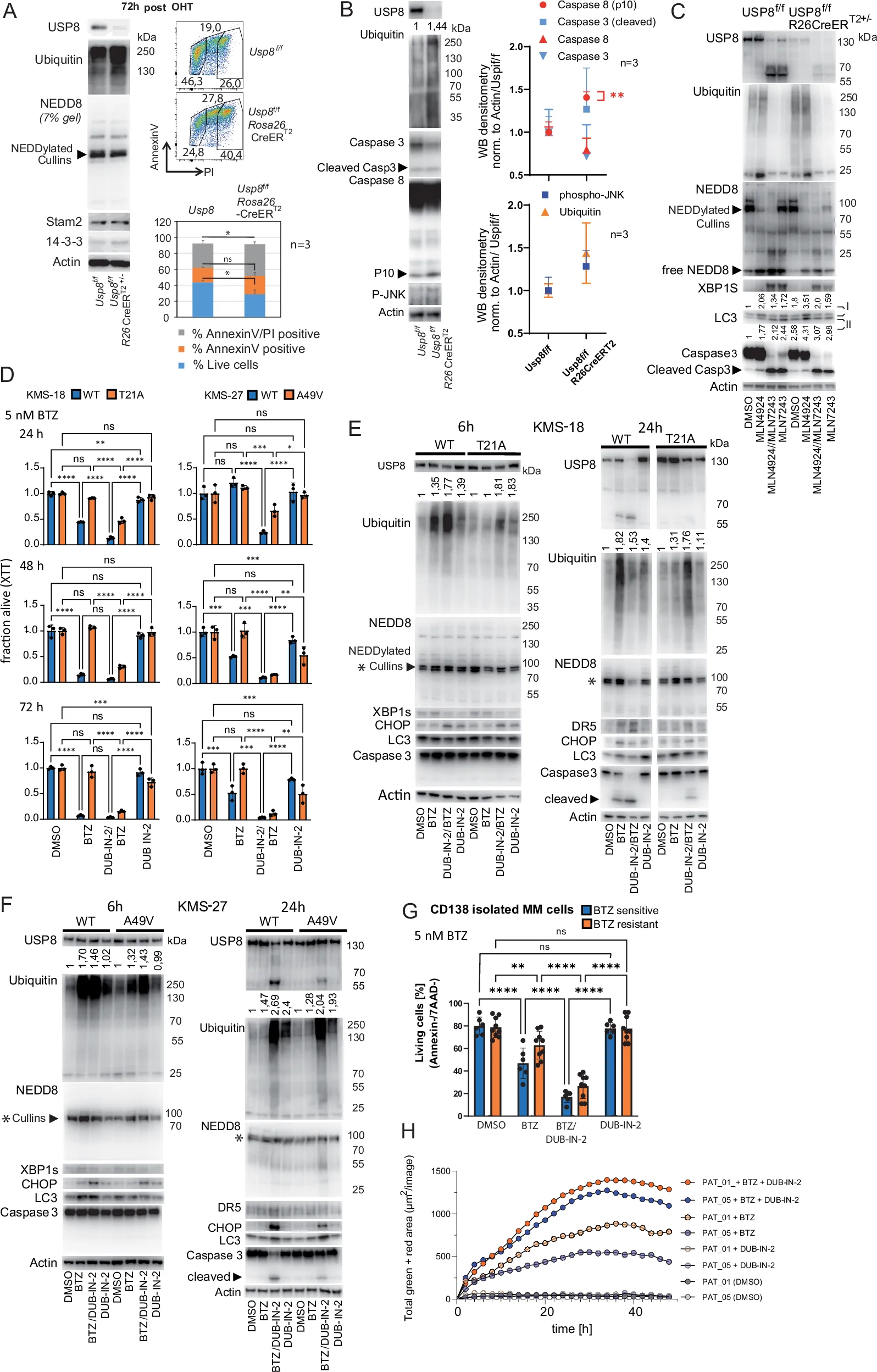
USP8 depletion and BTZ/DUB-IN-2 treatment induce proteotoxic stress and apoptosis in proliferating B and MM cells, respectively; DUB-IN-2 resensitizes resistant MM cells to BTZ. **A** *Usp8*^f/f^ and *Usp8*^f/f^*Rosa26*-CreERT2 splenic B cells were expanded in IL-4/CD40-containing medium for 24 h followed by OHT addition for 72 h. Lysates were subjected to WB analysis (left panels) and apoptosis was detected by FACS analysis (right panels, *n* = 3, two-tailed unpaired t-test, mean + sd). **B** *Usp8*^f/f^ and *Usp8*^f/f^*Rosa26*-CreERT2 splenic B-cell lysates generated as in (**A**) were subjected to WB and expression was quantified (*n* = 3, two-tailed unpaired t-test, mean + /-sd). **C** *Usp8*^f/f^ and *Usp8*^f/f^*Rosa26*-CreERT2 splenic B-cells were expanded as in (**A**) and treated with MLN4924 (NAE inhibitor, 500 nM) and/or MLN7243 (UBA1 inhibitor, 500 nM) for 16 h before lysis and WB analysis. LC3 I/II quantification is indicated. **D** Cell viability of KMS-18 or KMS-27 WT cells and BTZ-resistant mutant MM cells (KMS-18-T21A/KMS-27-A49V) treated with BTZ (5 nM), DUB-IN-2 (25 µM), or both (*n* = 3, 2-way ANOVA, Tukey’s post hoc test, mean + /-sd). WB analysis of KMS-18/KMS-18-T21A (**E**) or KMS-27/KMS-27-A49V (**F**) MM cells after exposure to BTZ (5 nM) and/or DUB-IN-2 (25 µM). **G** BM cells from MM patients defined as “BTZ resistant” (*n* = 9) or “non-resistant” (*n* = 6) were incubated with solvent (DMSO), BTZ (5 nM), DUB-IN-2 (25 µM), or BTZ + DUB-IN-2 for 24 h and viability was measured by AnnexinV/7AAD staining. (2-way ANOVA, Tukey’s post hoc test, mean + /-sd; two sided t-test was used to compare the response to BTZ alone across groups, *p* = 0.03). **H** MM cells from selected patients were stained with CytoLight Rapid Green and treated with inhibitors as in (**G**). Ongoing staining of apoptotic cells was performed with Annexin V Red Dye. WB results in Fig. 4 are representative of at least 2 independent experiments. Quantification of ubiquitin modification shown on top of each blot represents the average of at least 2 independent experiments normalized to actin and DMSO controls. Source data are provided as source data file.

Consistent with proteotoxic stress induced by inactive USP8, OHT-mediated USP8 deletion in primary *Usp8*^f/f^*Rosa26*-CreERT2 B cells increased overall ubiquitylation and accelerated apoptosis (Fig. 4A). A modest rise in JNK activation was observed, along with cleavage of Caspase-8 and loss of the full-length Caspase-8 and Caspase-3 proteins (Fig. 4B).

Remarkably, hyperNEDDylation (indicative of mixed ubiquitin/NEDD8 chains) was not observed after USP8 deletion in primary B cells. Because hyperNEDDylation may require sufficient free NEDD8 [40], USP8-depleted cells were treated with MLN4924 to block canonical Cullin NEDDylation and increase free NEDD8. However, this also failed to induce hyperNEDDylation (Fig. 4C), suggesting that mixed chains form only in certain cell-types or may be removed by enzymes other than USP8.

USP8 depletion in primary B cells induced autophagy, indicated by increased lipidated LC3-II, but also impaired autophagic flux, as shown by accumulation of LC3 (Fig. 4C).

To assess ER stress, XBP1s expression was monitored [47]. ER stress occurred upon UBA1-inhibition with MLN7243, but not after USP8 depletion. Notably, UBA1 inhibition triggered USP8 cleavage, correlating with Caspase 3 activation (Fig. 4C).

Together these data indicate that loss of USP8 increases protein ubiquitylation and triggers proteotoxic stress-induced apoptosis in B-cells without inducing ER stress.

### DUB-IN-2 controls proteostasis and survival of MM cells in synergy with BTZ

Given USP8’s role in preventing proteotoxic stress and its identification as a vulnerability gene, we evaluated its function in MM, where malignant plasma cells are highly sensitive to disrupted protein homeostasis [1, 24]. We hypothesized that USP8 inhibition could enhance proteotoxic stress and resensitize BTZ-resistant MM cells. Using the reported USP8 inhibitor DUB-IN-2 [10–12, 18], we tested KMS-18 and KMS-27 cells, including BTZ-resistant variants carrying PSMB5 mutations (T21A or A49V) [30].

KMS-18 and KMS-27 MM cells, including BTZ-resistant variants, were treated with BTZ, DUB-IN-2, or both. Single-agent treatment was ineffective in resistant cells, and DUB-IN-2 was also ineffective in WT cells. However, the combination drastically decreased cell viability of both WT and BTZ-resistant cell lines (Fig. 4D). In KMS-18-T21A cells, 25 µM DUB-IN-2 was required to overcome BTZ resistance (Fig. S5A). A similar synergistic effect was observed with CFZ and DUB-IN-2 in KMS-27 WT and CFZ-resistant KMS-27-A49V cells [30] (Fig. S5B). Synergy analysis using SynergyFinder Plus [48] confirmed positive interaction scores (Tables S2–S5). Combination treatments induced rapid accumulation of polyubiquitylated proteins within 6 h (Fig. 4E/F; Figure S5C). Increased NEDDylation was observed only in KMS-27/KMS-27-A49V cells after 24 h, indicating a cell type-specific response (Fig. 4F). Similar to UBA1 inhibition in primary B cells, 24 h BTZ treatment triggered USP8 degradation and caspase 3 activation in KMS-18 WT cells, effects enhanced by DUB-IN-2 co-treatment in all cell lines (Fig. 4E/F, right panels). Consistent with proteasome inhibition triggering apoptosis through ER stress in MM cells [49], the ER stress marker CHOP and its target, death receptor 5 (DR5) [47], were upregulated most strongly with combination treatment (Fig. 4E/F, Fig. S5C). XBP1s did not parallel CHOP upregulation, suggesting cell type-specific regulation of pro-apoptotic versus pro-adaptive stress responses [50, 51]. Autophagy induction, indicated by LC3-II accumulation, was not observed (Fig. 4E/F, Fig. S5C).

To validate these findings in patient derived MM cells with heterogenous resistance mechanisms, BTZ/DUB-IN-2 treatment was tested in MM cells isolated from 9 BTZ-resistant and 6 BTZ-responsive patients. The two groups differed significantly in BTZ response (*p* = 0.03) (Fig. 4G). Combination treatment, using BTZ at the most discriminatory dose and DUB-IN-2 at the highest non-toxic concentration (Fig. S5D), reduced viability across samples (Fig. 4G/H, Table S6).

Overall, these data suggest that DUB-IN-2 can overcome BTZ/CFZ resistance by enhancing ER stress, highlighting its therapeutic potential.

### DUB-IN-2 impacts MM cell viability independently of USP8

**Fig. 5:**
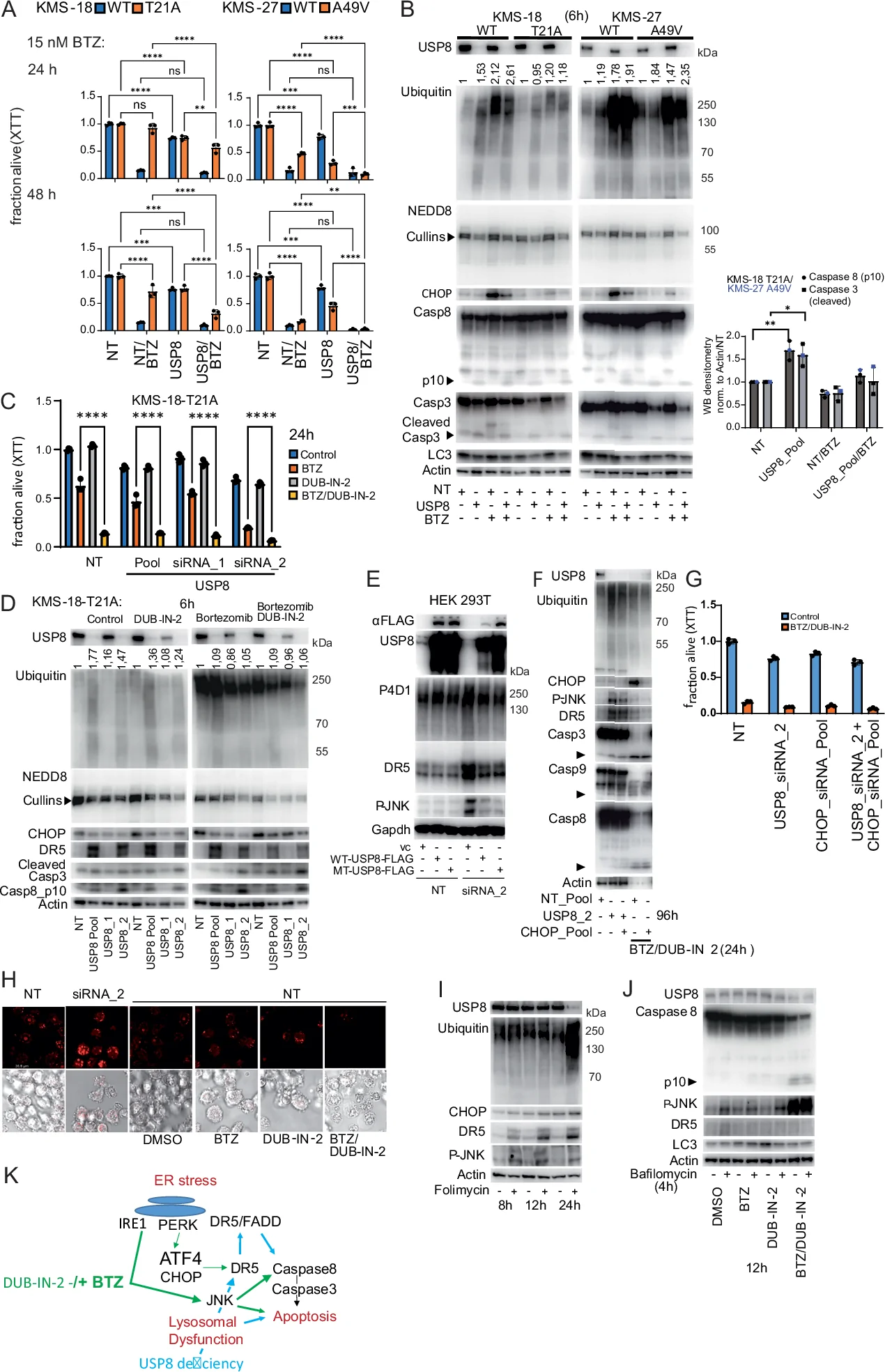
DUB-IN-2 exerts USP8-independent effects on cell viability. **A** Viability of KMS-18/KMS-27 WT and mutant MM cells transfected with pooled (4 siRNAs) non-targeting (NT) or USP8 siRNA for 72 h followed by BTZ (15 nM) treatment as indicated (*n* = 3, 2-way ANOVA, Tukey’s post hoc test, mean + /-sd). **B** MM cells transfected as in (**A**) for 96 h, followed by treatment with BTZ for 6 h and WB analysis. Caspase processing was quantified (*n* = 3, two-tailed unpaired t-test, mean + /-sd; significant results indicated). **C** Viability of KMS-18-T21A MM cells transfected with pooled NT siRNA, or pooled/individual USP8 siRNA_1/2 for 72 h followed by 24 h BTZ (25 nM) and/or DUB-IN-2 (25 µM) treatment (*n* = 3, 2-way ANOVA, Tukey’s post hoc test, mean + /- sd). **D** KMS-18-T21A MM cells transfected as in (**C**), treated with BTZ (25 nM) and/or DUB-IN-2 (25 µM) for 6 h, analyzed by WB. **E** 293 T cells were transfected with USP8-FLAG (WT or silent mutations in the siRNA2 target sequence). USP8 siRNA_2 was transfected after 24 h, lysates for WB were generated after 96 h. **F** WB analysis of KMS-18-T21A MM cells transfected with pooled NT siRNA, USP8_siRNA_2, pooled CHOP-specific siRNA, or USP8- and CHOP-specific siRNA for 72 h followed by 24 h BTZ/DUB-IN-2 (25 nM/25 µM) treatment as indicated. Arrows indicate cleaved caspases. **G** Viability of KMS-18-T21A cells transfected as in (**F**) followed by 48 h inhibitor treatment (*n* = 3, mean + /-sd). **H** KMS-18-T21A cells transfected with pooled NT siRNA or USP8 siRNA_2 for 96 h, and treated with BTZ (25 nM) and/or DUB-IN-2 (25 µM) for 24 h, as indicated, analyzed by AO staining. Results representative of 5 pictures from 2 independent experiments, respectively. **I** KMS-18-T21A cells treated with Folimycin (100 nM) as indicated, analyzed by WB. **J** WB analysis of KMS-18-T21A MM cells treated with BTZ and/or DUB-IN-2 for 12 h. Bafilomycin (200 nM) added 4 h prior to lysis. **K** Differentially regulated apoptosis pathways triggered by proteotoxic stress upon USP8 depletion (blue) or DUB-IN-2/BTZ treatment (green). Green line thickness indicates DUB-IN-2 effect dependence on BTZ co-treatment. WBs in Fig. 5 are representative of at least 2 independent experiments. Ubiquitin quantification as in Fig. 4. Source data provided.

USP8 knockdown in KMS cells only partially mimicked DUB-IN-2 effects. Combined USP8 depletion and BTZ showed only additive effects or mild synergy, requiring 3-fold higher BTZ concentrations (Fig. 5A, Bliss scores [52] are listed in Table S7), with KMS-27-WT/A49V cells being more susceptible than KMS-18-WT/T21A cells. Unlike DUB-IN-2, USP8 depletion did not induce CHOP or enhance BTZ-induced CHOP as an indicator of ER stress, but activated Caspase 8 and Caspase 3 (Fig. 5B), suggesting DUB-IN-2 affects BTZ-resistant MM cells independently of USP8.

To test DUB-IN-2 specificity, USP8 was depleted in KMS-18-T21A cells before BTZ, DUB-IN-2, or combination treatment. Efficient USP8 knockdown (pooled siRNA or siRNA_2) still allowed DUB-IN-2 to further reduce viability, indicating its effects occur independently of USP8 (Fig. 5C).

As these results raised considerable concerns about DUB-IN-2’s USP8 specificity, we further analyzed consequences of USP8 depletion. Although CHOP was not induced, its target DR5 was strongly upregulated, correlating with caspase 8 activation; partial USP8 knockdown with siRNA_1 was insufficient (Fig. 5D). 6 h DUB-IN-2 and/or BTZ treatment alone had no effect on DR5 expression or caspase activation, but enhanced protein ubiquitylation. Reexpression of USP8 in 293 T cells prevented hyper-ubiquitylation, DR5 upregulation, and JNK activation, confirming on-target effects (Fig. 5E). Of note, JNK activation has previously been shown to act upstream of DR5 transcriptional activation [53] and thus may represent a CHOP-independent pathway of DR5 induction. CHOP knockdown in KMS-18-T21A cells (to rule out transient CHOP overexpression) showed DR5 induction occurs independently of CHOP, which is also dispensable for BTZ/DUB-IN-2-induced cell death (Fig. 5F, G). USP8 depletion-induced JNK activation, which triggers apoptosis via both extrinsic and intrinsic pathways [54], was accompanied by caspase 3, 9, and 8 activation (Fig. 5F).

As an ESCRT component, USP8 regulates lysosomal trafficking [3]. To compare consequences of USP8 depletion with DUB-IN-2 treatment, lysosomal integrity was analyzed in KMS-18-T21A and KMS-27-A49V cells. Lysosome enlargement, indicative of impaired degradation, occurred only upon USP8 depletion, but not after BTZ and/or DUB-IN-2 treatment (Figs. 5H and S5E). Pharmacological lysosomal inhibition with folimycin - similar to USP8 depletion - induced JNK phosphorylation and DR5 expression with little effects on CHOP or early ubiquitylation (Fig. 5I). Together, these results indicate that USP8 depletion impairs lysosomal degradation, a phenotype not recapitulated by DUB-IN-2 treatment, further questioning its specificity for USP8.

To analyze potential effects of DUB-IN-2 on autophagic degradation, KMS-18-T21A cells were treated with bafilomycin ± DUB-IN-2 and/or BTZ. No increase in LC3-II was detected, indicating no evidence for authophagy induction or altered autophagic flux. In line with pro-apoptotic IRE1 signaling [50, 51], BTZ/DUB-IN-2 co-treatment induced JNK and caspase 8 activation after 12 h, while DR5 expression remained unchanged (Fig. 5J). These results suggest that BTZ/DUB-IN-2-induced apoptosis is mediated by ER stress-driven JNK independently of DR5, whereas USP8 depletion promotes JNK activation, DR5 upregulation, and apoptosis primarily via lysosomal dysfunction [55] (Fig. 5K). The distinct responses, including lysosomal defects and lack of CHOP induction upon USP8 silencing, indicate that DUB-IN-2 acts independently of USP8.

**Fig. 6:**
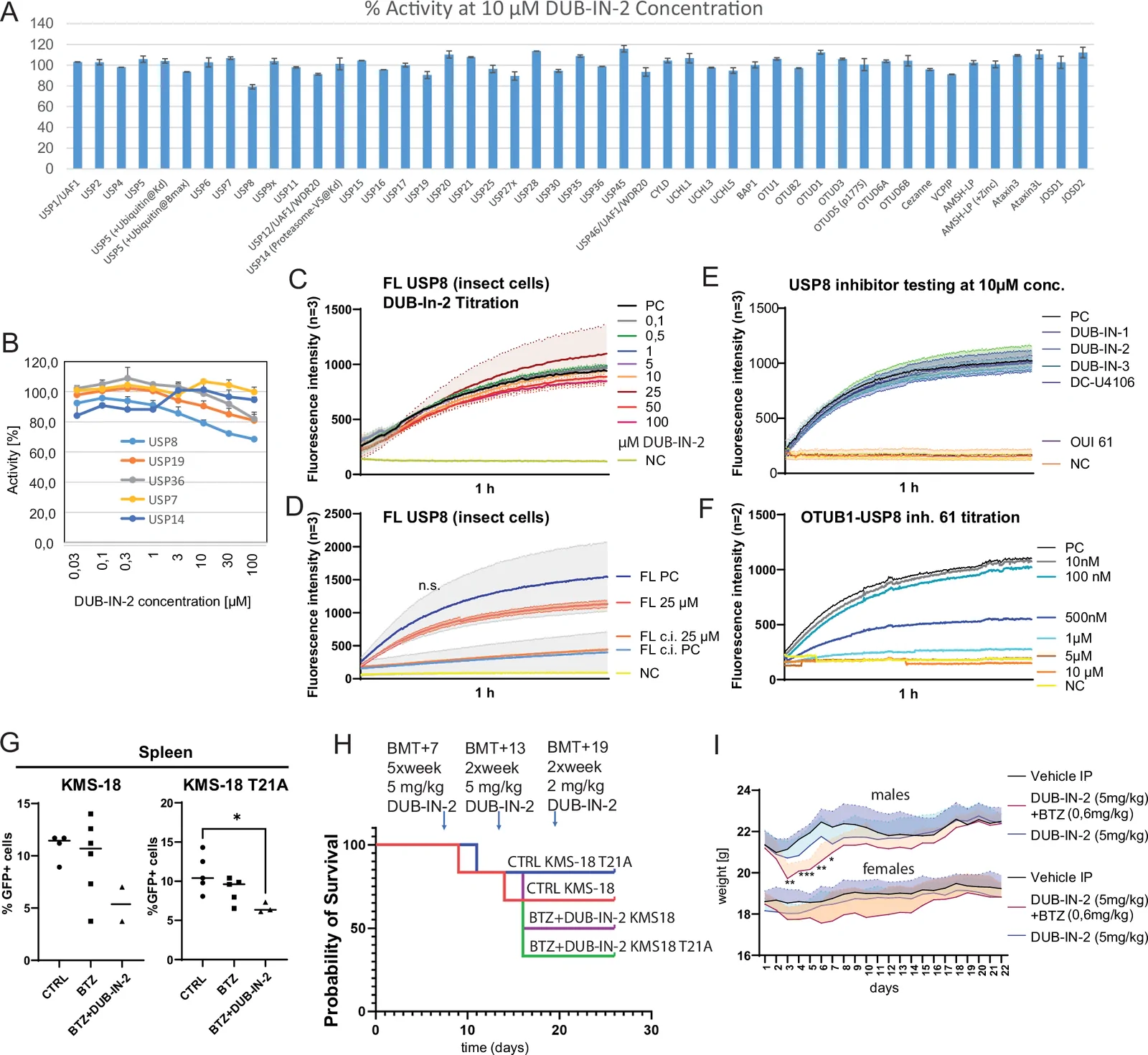
Therapeutic potential of DUB-IN-2 in MM. **A** Evaluation of the selectivity of DUB-IN-2 (10 µM) across the DUB*profiler* panel (Ubiquigent). +Ubiquitin@kd/+Ubiquitin@Bmax: Enzyme activated half-maximally/maximally with additional ubiquitin; Proteasome-V5@kd: Enzyme activated with proteasome-V5. (Mean of duplicate experiments +/-sd). **B** Effect of different DUB-IN-2 concentrations on selected DUBs. (Mean of duplicate experiments +/-sd). **C** Evaluation of different DUB-IN-2 concentrations for inhibiting USP8 expressed in insect cells using ubiquitin-rhodamine as a substrate (*n* = 3; PC positive control, NC negative control (no enzyme)). **D** Assessment of DUB-IN-2 (25 µM) effects on USP8 and a catalytically inactive (c.i.) variant (C786R) purified from insect cells (*n* = 3). **E** Assessment of commercially available USP8 inhibitors (10 µM) for their effects on bacterially expressed USP8 (*n* = 3). **F** Effect of OTUB1/USP8 inhibitor 61 (OUI61) on bacterially expressed USP8 across different concentrations (*n* = 2). **G** Infiltration of MM cells in spleens of NSG mice treated with either control (0.9NaCl/PEG300 1:1 (v/v)), BTZ (BTZ, 0.6 mg/kg, twice a week), or BTZ + DUB-IN-2 (*n* = 6) using 5 mg/kg 5 times a week over a period of 13 days, followed by 2 mg/kg twice a week in spleen (1-way ANOVA, Tukey’s post hoc test). **H** Kaplan–Meier plot showing the toxicity of the DUB-IN2/BTZ combination treatment (5 mg/kg). **I** Weight curves of C57BL/6 J mice (*n* = 5) IP injected with 0.9NaCl/PEG300 1:1 (v/v)), BTZ (BTZ, 0.6 mg/kg twice a week), or BTZ combined with DUB-IN-2 using 5 mg/kg 5 times a week over a period of 22 days. (T-test: combination treatment compared to vehicle control). Source data are provided as source data file.

The profound differences in cell death mechanisms prompted us to reevaluate DUB-IN-2 specificity in biochemical assays. A DUB profiling assay (Ubiquigent, >40 recombinant DUBs) showed no inhibition of USP8 or other DUBs across 30 nM–100 µM (Fig. 6A/B). Consistently, in vitro assays with insect cell or bacterially expressed USP8 confirmed that DUB-IN-2, as well as DUB-IN-1, DUB-IN-3 [18], and DC-U4106 [19], did not inhibit USP8-activity (Fig. 6C–E). In contrast, the dual OTUB1/USP8 inhibitor 61 (OUI61) inhibited USP8 activity at ≥500 nM (Fig. 6E/F). but induced caspase-independent cell death without increased ubiquitylation, indicating off-target toxicity (Figure S6A/B). DUB-IN-2 also did not affect proteasome activity (Fig. S6C). Together, these data demonstrate that DUB-IN-2 does not inhibit USP8 and that its effects on MM viability are USP8-independent.

Although USP8 activity was unaffected, DUB-IN-2 treatment effectively overcame BTZ resistance in MM cells. We therefore evaluated BTZ/DUB-IN-2 co-treatment using NSG mice transplanted with KMS-18 or KMS-18-T21A cells. While BM infiltration was limited (Fig. S6D), stable spleen engraftment revealed reduced tumor expansion upon combination treatment (Fig. 6G). However, notable toxicity was observed under these conditions (Fig. 6H). To evaluate toxicity in a less vulnerable setting, treatment was tested in non-irradiated, immune-competent mice. Here, BTZ/DUB-IN-2 caused only transient weight loss without affecting survival or inducing gross pathological changes (Fig. 6I, Bienta Study Report T011125a). These findings support therapeutic efficacy of the combination and suggest a potentially manageable toxicity profile that may warrant further evaluation in humans.

To explore the potential clinical relevance of USP8 in MM, we next analyzed patient data from the MMRF-COMMPASS cohort. Lower USP8 expression at diagnosis was associated with a trend toward improved overall survival (Fig. S7A), and USP8 expression was significantly higher in stage III compared with stage II disease (Fig. S7B). Notably, these analyses are based on RNA expression levels and may not fully reflect USP8 protein abundance.

In summary, we define USP8 as a key regulator of proteostasis in B cells. Although DUB-IN-2 is not a specific USP8 inhibitor, this compound shows strong anti-MM activity in synergy with BTZ. These findings suggest that both USP8 inactivation and DUB-IN-2 treatment may be exploited therapeutically to overcome BTZ resistance and induce apoptosis in B-cell malignancies.

## Discussion

Our data show that USP8 is critical for B-cell development and survival. Deleting *Usp8* at later stages, as in *Usp8*^f/f^*Cd19*-Cre mice, permits maturation beyond the pre-B-cell stage, resulting predominantly in immature, innate-like B cells, GC-like B cells, and PCs with reduced Roquin expression that drive normal (IgG_1_) or enhanced (IgM and IgG_3_) Ig production upon immunization. Roquin1/2, RNA-binding proteins with E3 ligase activity, shape T- and B cell responses by destabilizing mRNAs (e.g., *Ifng*, *Icos*) and controlling mTor activity [33]. Roquin loss expands the B-cell pool and triggers spontaneous GC activation [56], as observed in *Roquin*^*san/san*^ (sanroque) mice, which develop spontaneous GCs and autoimmunity [57]. Preventing GC formation in these mice did not abolish autoimmune disease, but suggested that the expanded B1 B-cell population and extrafollicular PCs contributed to IgM autoantibody production [58]. Thus, loss of Roquin may contribute to the heightened activation potential of USP8-depleted splenic B cells, including increased JNK activation. Gene expression data further suggest altered Rho-GTPase signaling upon USP8 depletion, although the underlying mechanisms likely depend on the in vivo microenvironment and altered B cell composition. Notably, while Cd19-Cre-mediated recombination reduces Usp8 expression across mature B-cell subsets at the transcript level, residual USP8 protein likely reflects delayed depletion kinetics after genomic deletion. Together with the phenotypic loss of mature B-cell populations observed in vivo, these findings support the conclusion that sustained USP8 expression is required for mature B-cell survival.

Proteotoxic-stress-induced B-cell defects in the absence of USP8 were first indicated in K46Lµm B cells expressing catalytically inactive USP8(C748A), where endogenous USP8 prevents cell death. USP8(C748A) traps substrates modified by mixed ubiquitin/NEDD8 chains [37], which are linked to aggresome-like body formation and autophagy [59], and nuclear protein aggregation under proteotoxic stress [44]. Consistent with prior studies [42, 43, 60], ubiquitin(K63) NEDDylation emerges as a proteotoxic stress signature, and we identify K48 ubiquitylation of NEDD8 as a USP8 target.

Our data indicate that disturbed proteostasis drives cell death in proliferating USP8-deficient B cells, explaining the developmental defects. Using MM as a model, we show that enhancing proteotoxic stress via DUB-IN-2 or USP8 knockdown has translational potential. The limited accumulation of mixed NEDD8/ubiquitin chains upon USP8 depletion appears cell type-specific and correlates with the increased sensitivity of primary B cells and MM cells to proteotoxic stress. A possible explanation is that endogenous mixed-chain intermediates are highly transient or efficiently cleared in these cells, thereby being preferentially captured under trapping conditions rather than accumulating upon genetic deletion alone.

BTZ-resistant MM cells were effectively targeted by combined DUB-IN-2/BTZ treatment, whereas DUB-IN-2 alone had minimal impact on viability. Interpretation of patient data is limited, as most “BTZ-resistant” cases received combination therapies, making true BTZ resistance difficult to define. However, given that BTZ/dexamethasone are typically used with modern agents, our cohort reflects current clinical practice.

MM cells rely on unfolded protein response (UPR) signaling to cope with high protein load [51], and both BTZ [49] and DUB-IN-2 synergistically induce apoptotic ER stress by targeting the UPS independently of USP8. In contrast, USP8 depletion impairs lysosomal function and reduces viability via a distinct mechanism. Emerging evidence suggests that severe disturbances in proteostasis can promote immunogenic cell death and enhance anti-tumor immune responses through the release of DAMPs and activation of type I interferon signaling [61, 62]. In MM, where plasma cells are highly dependent on proteostasis pathways, this raises the possibility that USP8 inhibition may not only induce tumor cell-intrinsic cytotoxicity but could also enhance tumor immunogenicity and thereby sensitize cells to T-cell–based immunotherapies. Recent work further supports a link between dysregulated deubiquitination pathways and interferon/ICD-like programs in MM [63]. However, multiple assays in this study document that DUB-IN-2 does not directly inhibit USP8, the proteasome, or other DUBs. However, overlapping effects previously reported upon USP8 depletion and DUB-IN-2 treatment may point to converging or partially shared pathways affected by these interventions. Thus, prior and future studies using DUB-IN-2 should be interpreted with caution, and conclusions drawn from its use may warrant reconsideration.

Despite these limitations, in vivo and patient-derived data shown here support further exploration of DUB-IN-2–based combination therapies. Identifying its molecular target(s) will be critical for further drug development. However, the current in vivo data indicate that anti-tumor efficacy and systemic tolerability are not yet fully aligned across model systems, highlighting that a defined therapeutic window will require further optimization in clinically relevant settings. Likewise, while targeting USP8 remains a promising strategy due to its role in proteostasis, cancer progression, and immune regulation, its essential functions in development and lymphocyte homeostasis warrant careful consideration of potential safety risks. Nevertheless, the accessibility of the hematopoietic system to small molecules and the potential for selective pharmacological modulation position USP8 as a highly promising therapeutic target.

## Supplementary information

Suppl Figures Tables Material and Methods

Related Manuscript File

Source data file - Western blots

## Data Availability

All MS proteomics data have been deposited to the ProteomeXchange Consortium via the PRIDE partner repository. The Shotgun analysis and the anlysis of GlyGly-K modification sites shown in Fig. 3B were registered under the data set identifier PXD024395 (Username: reviewer_pxd024395@ebi.ac.uk; Password: A6X1HcXc). The pull down-MS analysis shown in Fig. 3E was registered under the data set identifier PXD024755 (Username: reviewer_pxd024755@ebi.ac.uk; Password: Qgnx23Ev). The pull down-MS analysis shown in Fig. 3H was registered under the data set identifier PXD024429 (Username: reviewer_pxd024429@ebi.ac.uk; Password: FSW5Cl8e). Single cell RNA-seq analysis data are available at https://www.ncbi.nlm.nih.gov/geo/query/acc.cgi?acc=GSE244334 (access token: sxsdsecavdyxbqj). Materials generated in this study are available upon request.
