# Supplementary material for: USP8 controls proteostasis pathways in B cells and multiple myeloma
Source: Leukemia. 2026 Aug 11;40(11):2441–54. doi: 10.1038/s41375-026-03086-y (PMC13713586; doi:10.1038/s41375-026-03086-y)
Supplement: Supplementary file 1 [file 41375_2026_3086_MOESM1_ESM.pdf]

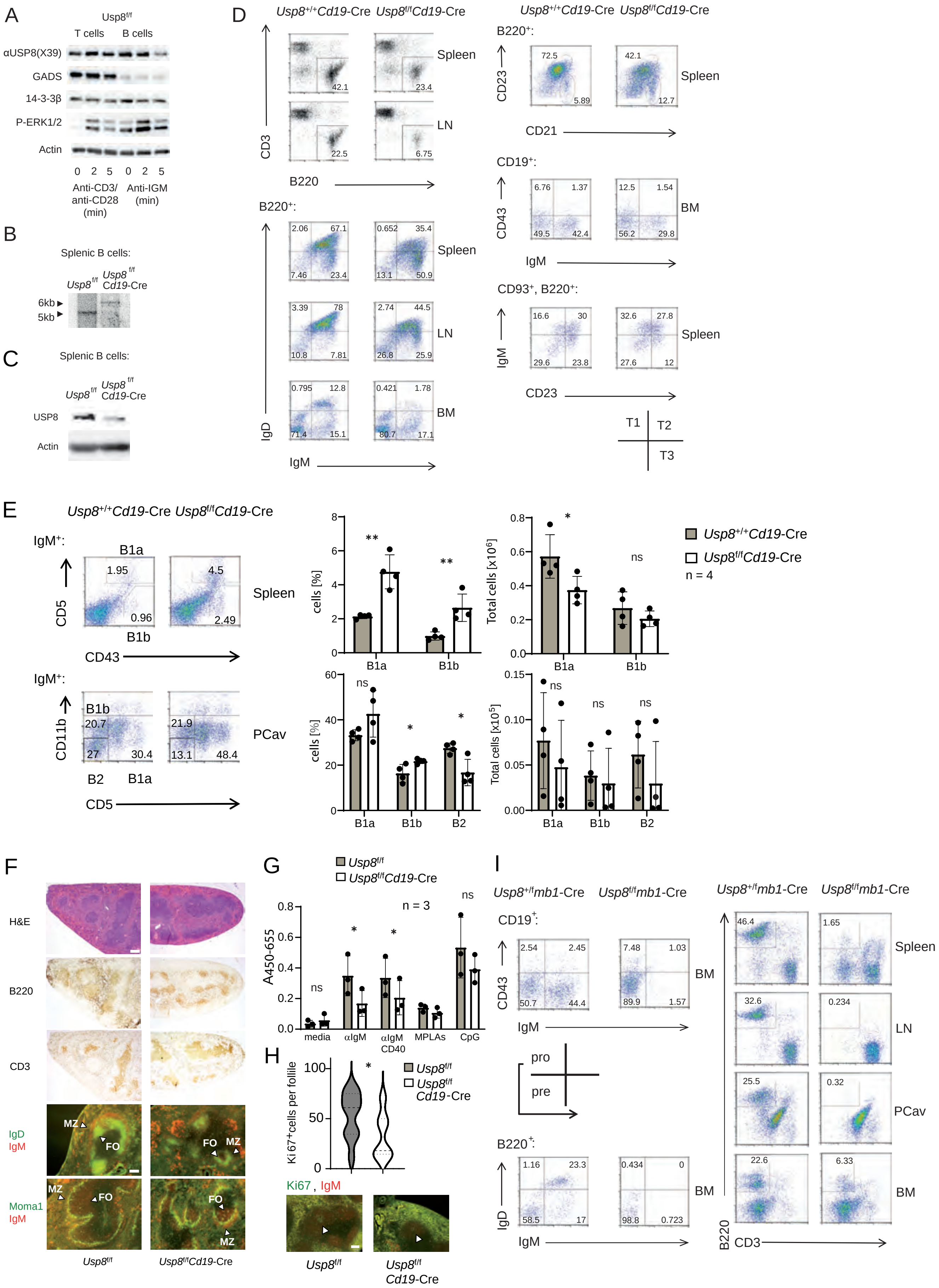

Dufner et al., Figure S1

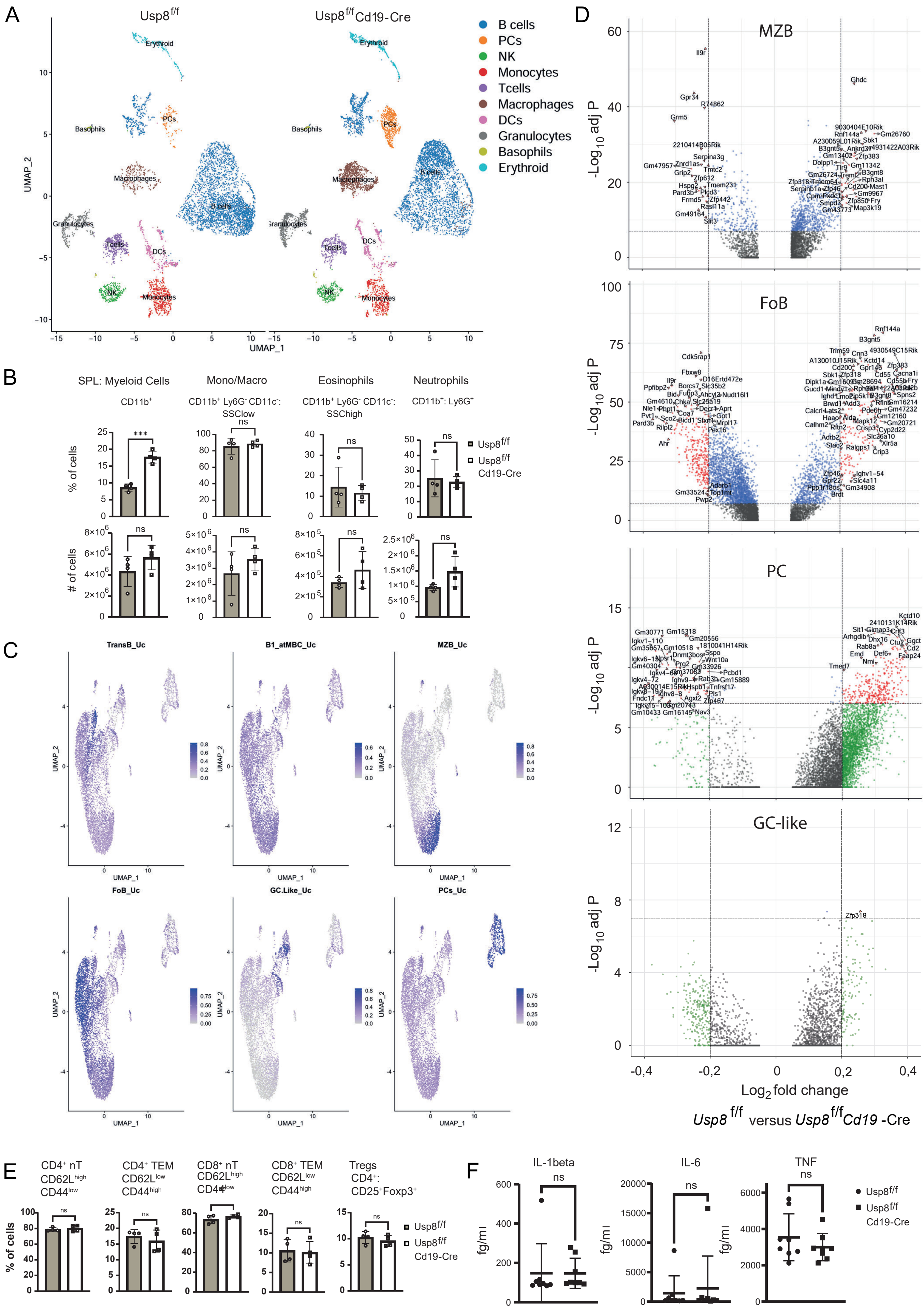

Dufner et al., Figure S2

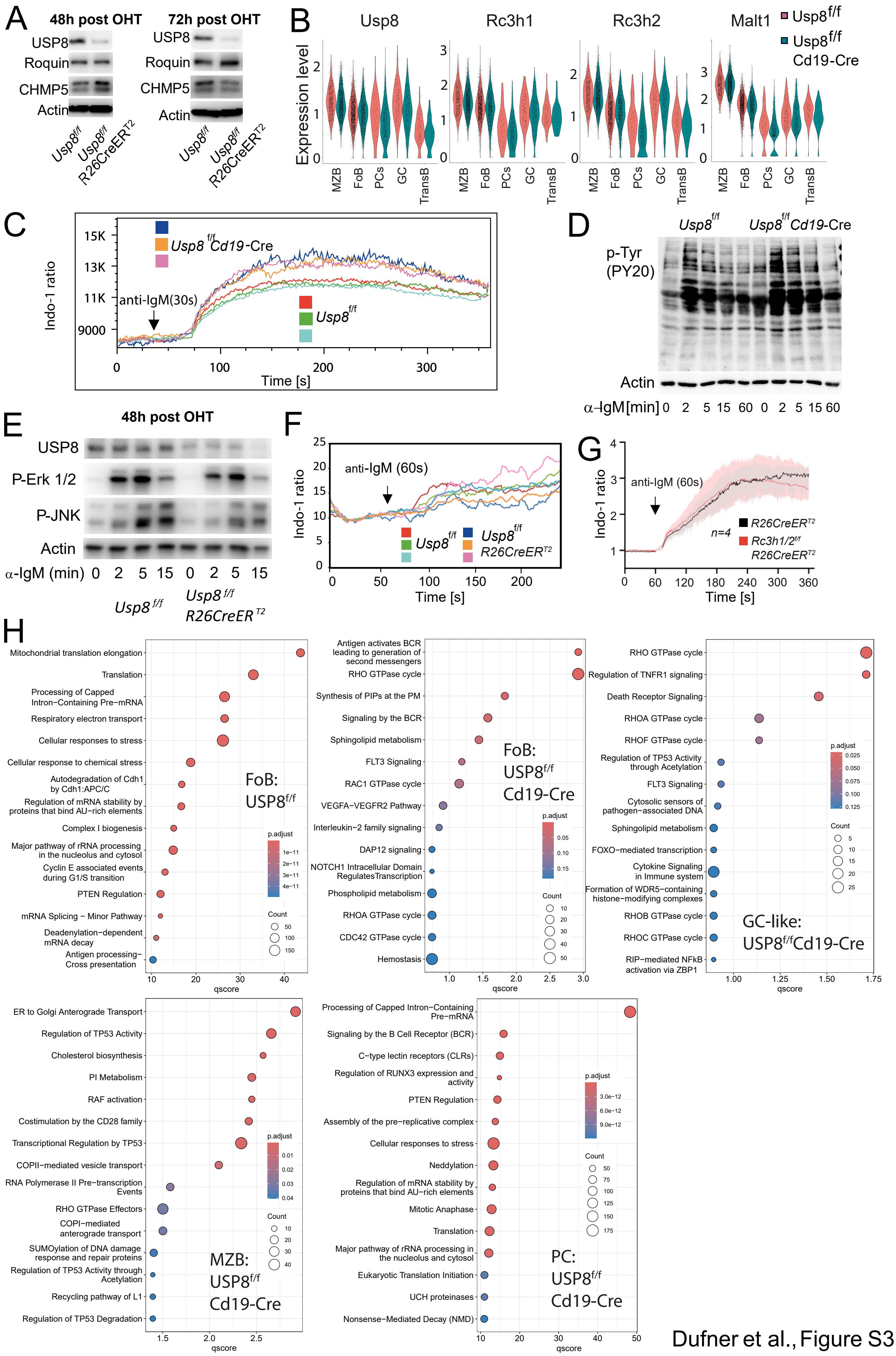

Dufner et al., Figure S3

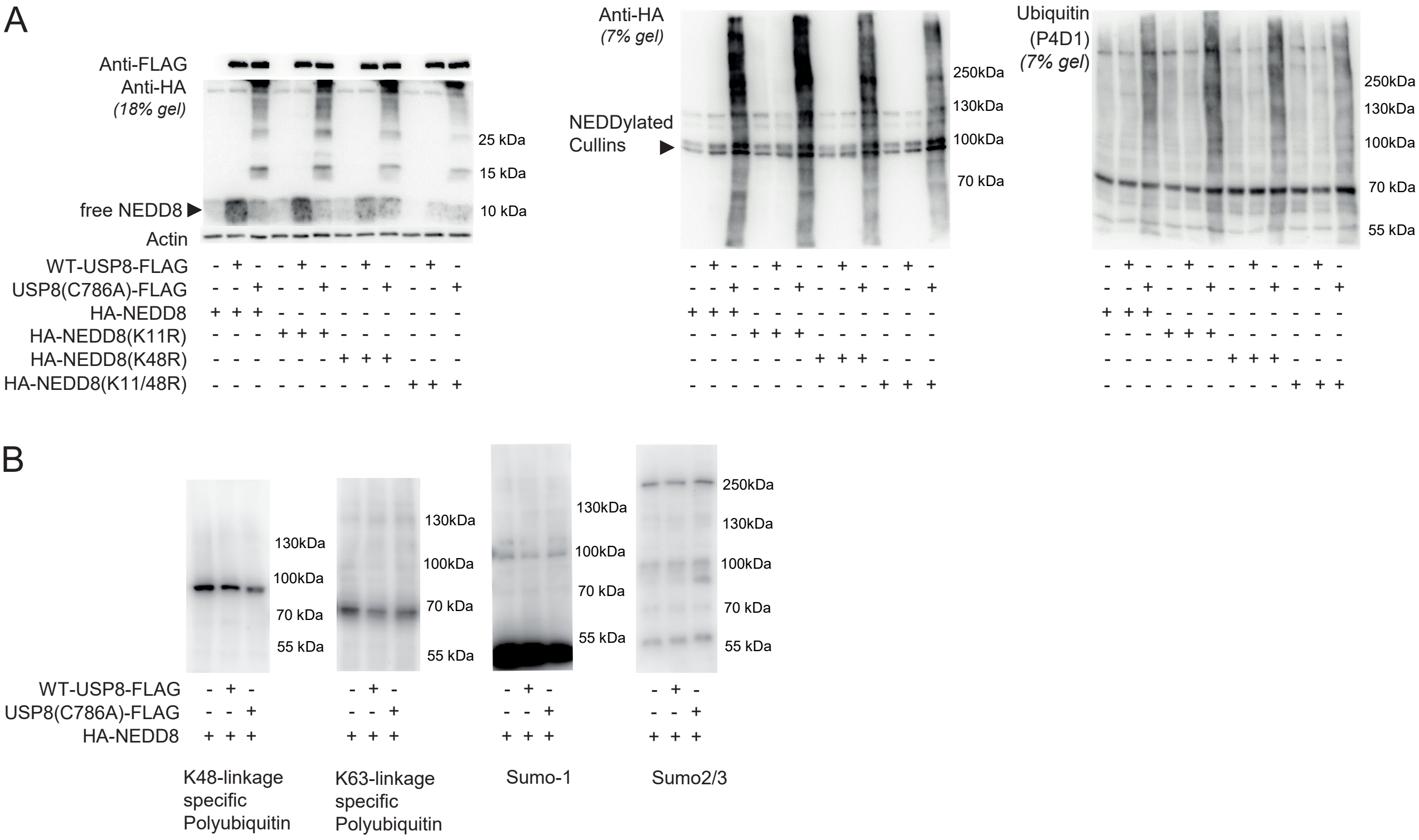

Dufner et al. Figure S4

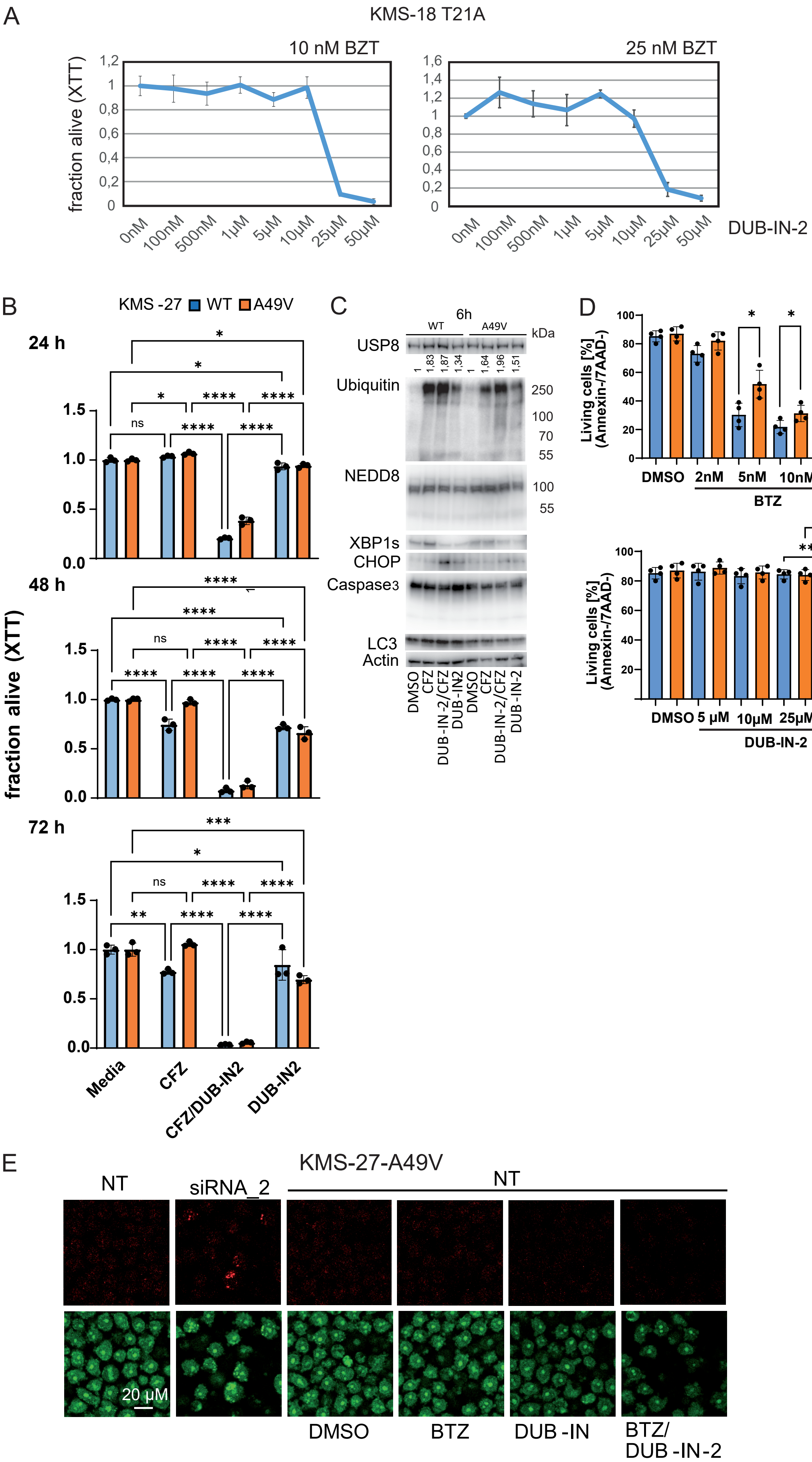

Dufner et al., Figure S5

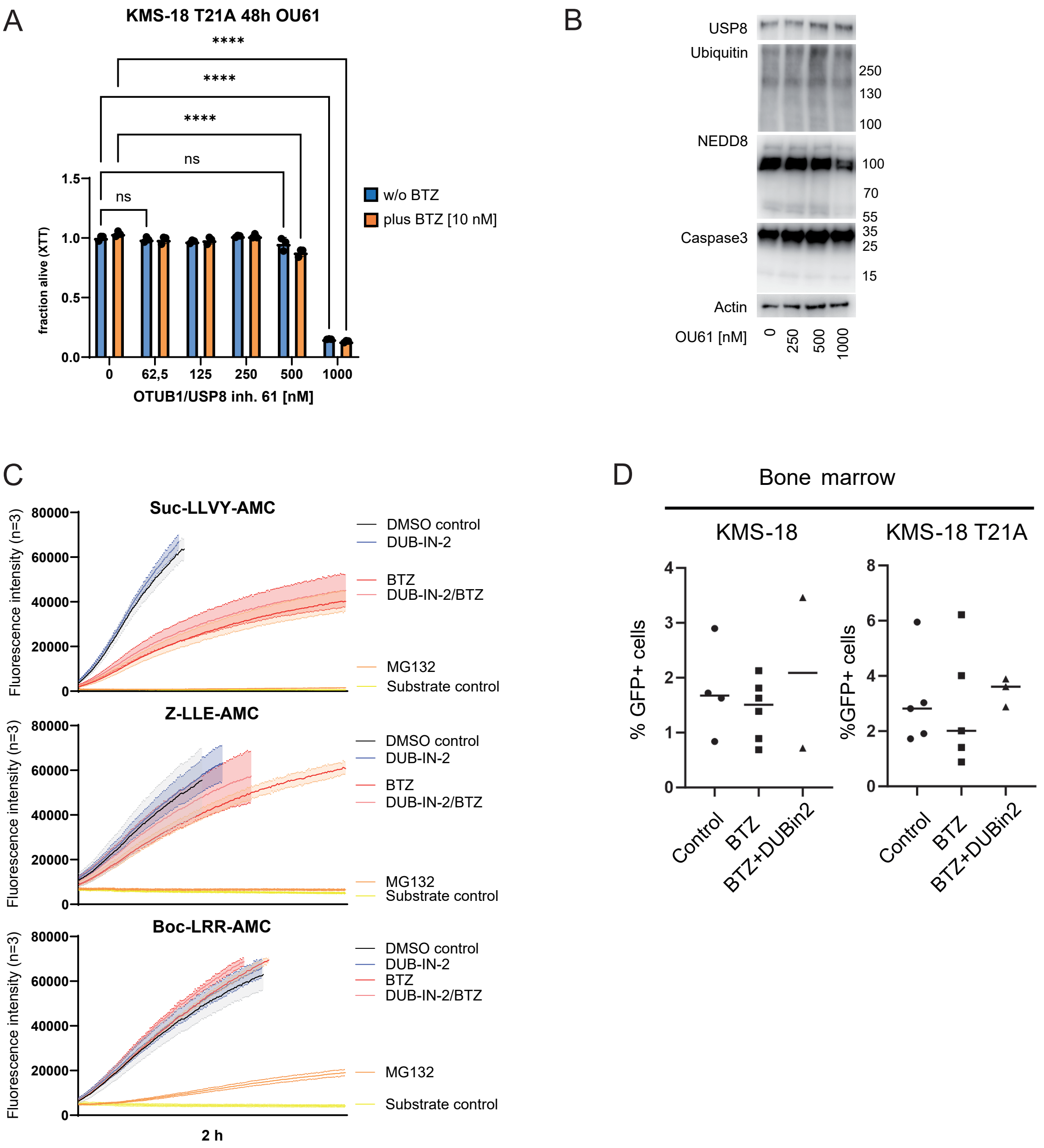

Dufner et al., Fig. S6

A

B

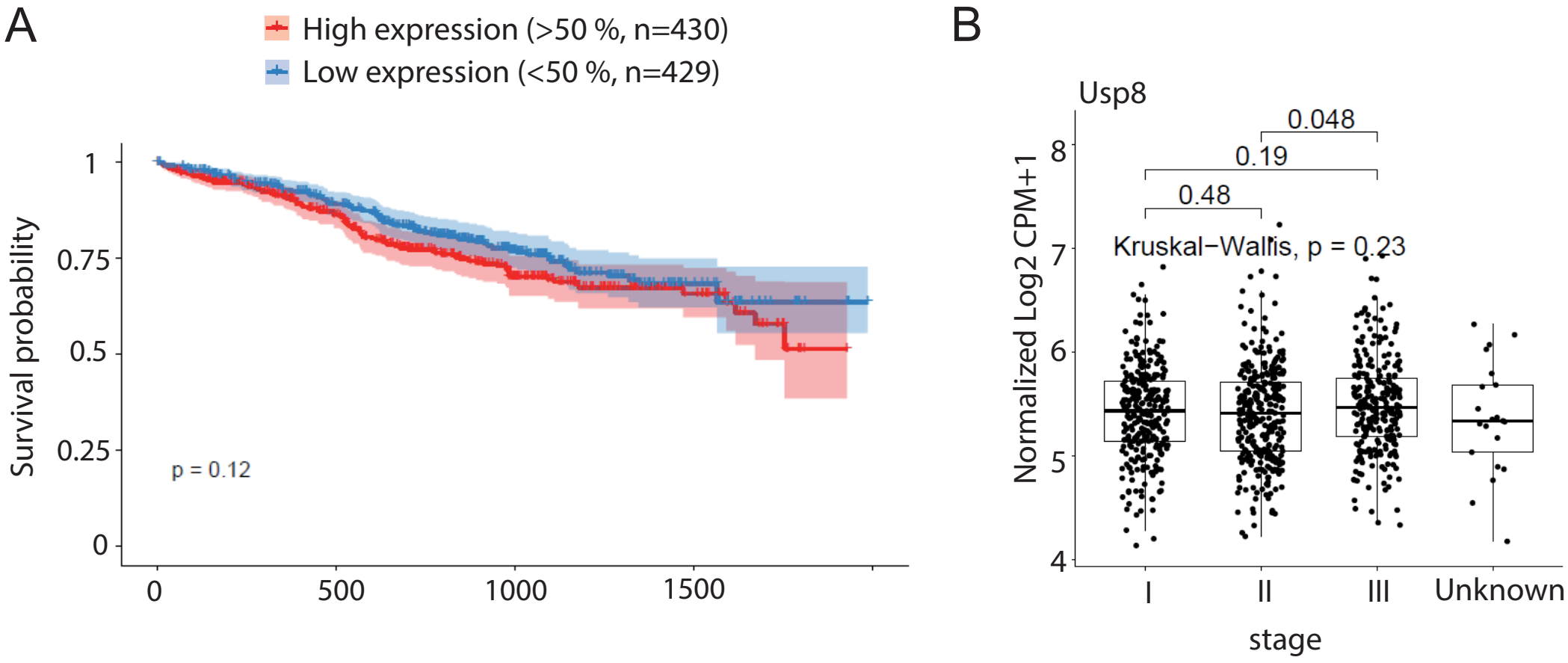

Dufner et al., Fig. S7

Supplemental Figure Legends

Fig. S1. Defect in B-cell development in *Usp8*$^{f/f}$*Cd19*-Cre mice. (A) Marginal GADS expression in B cells as compared to T cells. Splenic T and B cells were enriched and stimulated for the times indicated with anti-CD3/CD28 or anti-IgM F(ab')$_2$ fragment antibody, respectively. Lysates were analyzed by WB. (B) Southern blot analysis of NcoI-digested genomic DNA from *Usp8*$^{f/f}$ control and *Usp8*$^{f/f}$*Cd19*-Cre B cells reveals efficient *Usp8* gene recombination. Deletion is indicated by a 6kb band representing the deleted allele. The 5kb band represents the floxed allele. (C) USP8 and actin expression in splenic B cells were analyzed by WB. (D) Flow cytometry analysis of B-cell subsets in spleen, LN, and BM of *Usp8*$^{+/+}$*Cd19*-Cre mice and *Usp8*$^{f/f}$*Cd19*-Cre mice. Pre-gating is indicated at the upper left of the FACS plots. Results are representative of 4 independent experiments. (E) Flow cytometry analysis of B1a- and B1b-cell subsets in spleen and peritoneal cavity (PCav), and B2 cell subsets in PCav of *Usp8*$^{+/+}$*Cd19*-Cre and *Usp8*$^{f/f}$*Cd19*-Cre mice. Pre-gating is indicated at the upper left of the FACS plots. The percentages and total numbers of B1 and B2 cell subsets are shown on the right (n=4, unpaired 2-tailed t-test, mean+/-sd). (F) Immunohistochemistry staining of paraffin-embedded spleen sections with hematoxylin/eosin (H&E), anti-B220, and anti-CD3 (brown) (scale bar 200 µm), and co-staining of frozen spleen sections as indicated (scale bar 100 µm). (G) Proliferation (BRDU incorporation) of purified splenic CD19$^+$ *Usp8*$^{f/f}$ and *Usp8*$^{f/f}$*Cd19*-Cre B cells stimulated with anti-IgM in the absence or presence of anti-CD40, or with the TLR4 and TLR9 agonists "synthetic Monophosphoryl Lipid A" (MPLA) and CpG, respectively (paired, 2-tailed t-test representing n=3 sex-matched littermates, mean+/-sd). (H) Quantification of Ki67$^+$ *Usp8*$^{f/f}$ and *Usp8*$^{f/f}$*Cd19*-Cre B cells in proliferating Ki67$^+$ B-cell areas ($n \geq 10$, unpaired 2-tailed t-test, mean+/-sd). Scale bar, 100µm. (I) B-cell subsets in BM, spleen, LN, and PCav of *Usp8*$^{+/f}$mb1-Cre mice and *Usp8*$^{f/f}$*mb1*-Cre mice were analyzed by

flow cytometry. Pre-gating is indicated at the upper left of the respective FACS plots. Results are representative of 3 independent experiments. Source data are provided as source data file.

Fig. S2.

Altered B-cell subsets, but normal myeloid and T-cell distribution, and inflammatory blood cytokine levels in B cell-specific Usp8 KO mice. (A) UMAP projection of B cell enriched splenocytes colored by graph-based clusters. (B) Flow cytometry analysis of splenic myeloid cells derived from *Usp8*$^{f/f}$ and *Usp8*$^{f/f}$*Cd19*-Cre mice (n=4, unpaired 2-tailed t-test, mean+/-sd). (C) Expression scores based on Mann-Whitney U statistic of 10 genes specific for the cell type reported [64] in UMAP projection. (D) Volcano plots of differential gene expression analysis of MZB, FoB, PC, and GC-like B cells. Red dots indicate significance for adjusted P value and $\log_2$ fold change. (E) Flow cytometry analysis of splenic Tcell subsets derived from *Usp8*$^{f/f}$ and *Usp8*$^{f/f}$*Cd19*-Cre mice. nT, naïve T cells; TEM, effector memory T cells; Treg, regulatory T cells (n=4, unpaired 2-tailed t-test, mean+-s/d). (F) Inflammatory cytokine levels in serum of *Usp8*$^{f/f}$ and *Usp8*$^{f/f}$*Cd19*-Cre mice (n=8, unpaired 2-tailed t-test, mean+-s/d). Source data are provided as source data file.

Fig. S3. *Cd19*-Cre-mediated deletion of USP8 alters splenic B-cell compartment signaling. (A) *Usp8*$^{f/f}$ and *Usp8*$^{f/f}$*Rosa26*-CreERT2 splenic B cells were expanded in medium containing IL-4 and CD40 for 24 h followed by OHT addition and further expansion for 48 h or 72 h. Lysates were subjected to WB analysis. Results are representative of at least two independent experiments. (B) Gene expression of USP8, Roquin and Malt1 genes in splenic *Usp8*$^{f/f}$ and *Usp8*$^{f/f}$*Cd19*-Cre B-cell subsets. Black dots represent a single cell each. (C) Ca^{2+} influx in anti-IgM-stimulated B cells. Splenic *Usp8*$^{f/f}$ and *Usp8*$^{f/f}$*Cd19*-Cre B cells were purified with biotinylated anti-CD19 antibodies and IMag Streptavidin Particles plus-DM and starved for 2 hours. B cells were loaded with indo-

1-AM (5µg/ml) and activated with anti-IgM F(ab')$_2$ fragment antibody (10µg/ml). The graph shows the ratio of bound to unbound indo-1-AM as a measure of Ca^{2+} influx (n=3). (D) Splenic B cells were purified, starved for 2 h, stimulated for the indicated times with anti-IgM F(ab')$_2$ fragment antibody, and analyzed by WB. (E) *Usp8*$^{f/f}$ and *Usp8*$^{f/f}$*Rosa26*-CreERT2 splenic B cells were expanded in medium containing IL-4 and CD40 for 24 h followed by OHT addition and further expansion for 48 h. Cells were stimulated as in (D) and lysates were subjected to WB analysis. Results in D and E are representative of at least two independent experiments. (F) Cells were expanded as in (E) for 72 h after OHT addition and Ca^{2+} influx was analyzed as in (C) (n=3). (G) Upon oral gavage of tamoxifen, Roquin1/2 depleted *Rc3h1/2*$^{f/f}$ *Rosa26*-CreERT2 B cells and *Rosa26*-CreERT2 control cells were enriched from spleens and Ca^{2+} influx was analyzed as in (C) (n=4). (H) Pathway enrichment analysis on genes upregulated in B-cell subsets of the indicated genotype. Source data are provided as source data file.

Fig. S4. Role of K11- and K48-linked NEDD8 modifications in the formation of mixed ubiquitin/NEDD8 chains induced by catalytically inactive USP8. (A) HEK293T cells were transfected with WT-USP8-FLAG or USP8(C786A)-FLAG in the presence of the indicated HA-NEDD8 variants and lysates were analyzed by WB. (B) Selected samples shown in (A) were analyzed by WB as indicated. Results are representative of 2 independent experiments. Source data are provided as source data file.

Fig. S5. (A) Viability assay of BTZ resistant KMS-18-T21A MM cells upon treatment with 10nM (left panel) or 25 nM (right panel) BTZ in the presence of increasing DUB-IN-2 concentrations (n=3, mean+/-sd). (B) Cell viability of KMS-27 WT and BTZ-resistant mutant MM cells (A49V) treated with CFZ (3 nM), DUB-IN-2 (25 µM), or a combination of both for the times indicated

(n=3, 2-way ANOVA, Tukey's post hoc test, mean+/-sd). (C) WB analysis of KMS-27/KMS-27-A49V MM cells after exposure to CFZ and/or DUB-IN-2 for 6 h. Quantification of ubiquitin modification shown on top the blot represents the average of 2 independent experiments normalized to actin and DMSO controls. Source data are provided as source data file. (D) BM cells from selected MM patients who either insufficiently responded to BTZ-containing induction therapy or lost their response during BTZ-containing treatment (defined as "BTZ-resistant", n=4) and patients responding well to BTZ-containing induction therapy (defined as "BTZ non-resistant", n=4) were incubated with solvent (DMSO), or increasing concentrations of BTZ, or DUB-IN-2 for 24 hours and viability was measured by AnnexinV/7AAD staining. Unpaired 2-tailed t-test, mean+/- sd. (E) KMS-27-A49V cells transfected with pooled NT siRNA or USP8 siRNA_2 for 96 h, and NT siRNA transfected cells treated with BTZ (25 nM) and/or DUB-IN-2 (25µM) for 24 h were analyzed by AO staining. Results are representative of 5 pictures taken from 2 independent experiments, respectively.

Fig. S6. (A) Cell viability of KMS-18-T21A MM cells treated with the indicated concentrations of OTUB1/USP8 inhibitor 61 (OUI61) in the absence or presence of BTZ (10 nM) (n=3, 2-way ANOVA, Tukey's post hoc test, mean+/-sd). (B) OUI61 exerts a caspase 3-independent toxic effect on cell viability. KMS-18-T21A cells were treated with the indicated concentrations of OUI61 for 24 h and subsequently analyzed by WB. (C) Proteasome activity of KMS-18 MM cell lysates treated with MG132 (positive control), DUB-IN-2 (12.5 µM), and/or BTZ (7.5 nM) was assessed using fluorogenic substrates specific for chymotrypsin-like (Suc-LLVY-AMC), trypsin-like (Boc-LRR-AMC), and caspase-like (Z-LLE-AMC) activities. (D) Lack of BM infiltration of KMS-18/KMS-18-T21A MM cells in NSG mice that had been treated with either control (0.9NaCl/PEG300 1:1 (v/v)), BTZ (0.6mg/kg, twice a week), or BTZ combined with DUB-IN-2

(n=6) using 5mg/kg 5 times a week over a period of 13 days, followed by 2mg/kg twice a week (mean+/-sd).

Fig. S7. (A) Kaplan Meier survival analysis of MM patients included in the MMRF CoMMpass study according to USP8 expression at time of diagnosis (Log rank test). (B) Box plot of USP8 expression in patients of different MM disease stages (Kruskal-Wallis test).

Table S1. Neddylation-related genes upregulated in *Usp8*$^{f/f}$*Cd19*-Cre PCs.

**Commd8**	**Psmb1**	**Psma1**	**Psmc2**	**Uba3**
**Ube2d3**	**Psme2**	**Nedd8**	**Dda1**	**Klhl42**
**Psmb8**	**Ube2d2a**	**Fem1a**	**Psmb7**	**Rbbp7**
**Fbxl8**	**Psmd13**	**Keap1**	**Ercc8**	**Btbd1**
**Psmd4**	**Lrrc41**	**Psma4**	**Dcun1d1**	**Fbxo22**
**Psmb9**	**Psmc5**	**Uba52**	**Gan**	**Cul3**
**Asb13**	**Psmf1**	**Uchl3**	**Asb6**	**Fbxo7**
**Psmd5**	**Psma2**	**Cops5**	**Psma6**	**Fbxl14**
**Psme2b**	**Nub1**	**Commd6**	**Psmb4**	**Tulp4**
**Ufd1**	**Wsb2**	**Psme1**	**Wsb1**	**Nploc4**
**Dcaf11**	**Dcaf17**	**Ube2d1**	**Psmd12**	**Commd10**
**Cops7b**	**Psma3**	**Dcaf7**	**Commd9**	**Psmd6**
**Psmd10**	**Cul4b**	**Psmc1**	**Hif1a**	**Fbxw4**
**Psmd14**	**Dcaf13**	**Cand1**	**Mul1**	**Klhl20**
**Dcun1d5**	**Commd4**	**Ube2m**	**Ddb2**	**Ubxn7**
**Dcun1d2**	**Psmc3**	**Psma5**	**Spsb3**	**Psmd7**
**Psmb10**	**Psmd2**	**Ddb1**	**Cops4**	**Psmd11**
**Ube2f**	**Fbxo21**	**Commd2**	**Psmd1**	**Cul2**
**Fem1b**	**Psmc4**	**Cul5**	**Psmb2**	**Fbxo4**
**Psmc6**	**Fbxw9**	**Psma7**	**Cops2**	**Rnf7**
**Psmd8**	**Rbbp5**	**Commd7**	**Eloc**	**Rps27a**

Table S2

block_id	drug1	drug2	cell_line_name	conc1	conc2	response	conc_un
1	DUB-IN-2	Bortezomib	KMS-18	0	0	100	uM
1	DUB-IN-2	Bortezomib	KMS-18	6,25	0	101,9337487	uM
1	DUB-IN-2	Bortezomib	KMS-18	12,5	0	97,46706159	uM
1	DUB-IN-2	Bortezomib	KMS-18	25	0	96,46614238	uM
1	DUB-IN-2	Bortezomib	KMS-18	0	0,005	100,0544718	uM
1	DUB-IN-2	Bortezomib	KMS-18	6,25	0,005	98,86630579	uM
1	DUB-IN-2	Bortezomib	KMS-18	12,5	0,005	96,93936608	uM
1	DUB-IN-2	Bortezomib	KMS-18	25	0,005	89,78994315	uM
1	DUB-IN-2	Bortezomib	KMS-18	0	0,01	91,15173799	uM
1	DUB-IN-2	Bortezomib	KMS-18	6,25	0,01	81,91536445	uM
1	DUB-IN-2	Bortezomib	KMS-18	12,5	0,01	80,38674974	uM
1	DUB-IN-2	Bortezomib	KMS-18	25	0,01	42,38246008	uM
1	DUB-IN-2	Bortezomib	KMS-18	0	0,02	34,87216151	uM
1	DUB-IN-2	Bortezomib	KMS-18	6,25	0,02	26,03411296	uM
1	DUB-IN-2	Bortezomib	KMS-18	12,5	0,02	18,2037926	uM
1	DUB-IN-2	Bortezomib	KMS-18	25	0,02	5,484628741	uM
2	DUB-IN-2	Bortezomib	KMS-18 T21A	0	0	100	uM
2	DUB-IN-2	Bortezomib	KMS-18 T21A	6,25	0	103,2342692	uM
2	DUB-IN-2	Bortezomib	KMS-18 T21A	12,5	0	101,4996672	uM
2	DUB-IN-2	Bortezomib	KMS-18 T21A	25	0	96,42507537	uM
2	DUB-IN-2	Bortezomib	KMS-18 T21A	0	0,005	101,5309918	uM
2	DUB-IN-2	Bortezomib	KMS-18 T21A	6,25	0,005	101,9068875	uM
2	DUB-IN-2	Bortezomib	KMS-18 T21A	12,5	0,005	102,3845883	uM
2	DUB-IN-2	Bortezomib	KMS-18 T21A	25	0,005	94,2754219	uM
2	DUB-IN-2	Bortezomib	KMS-18 T21A	0	0,01	105,278202	uM
2	DUB-IN-2	Bortezomib	KMS-18 T21A	6,25	0,01	101,3626219	uM
2	DUB-IN-2	Bortezomib	KMS-18 T21A	12,5	0,01	100,3328243	uM
2	DUB-IN-2	Bortezomib	KMS-18 T21A	25	0,01	90,35592623	uM

2	DUB-IN-2	Bortezomib	KMS-18 T21A	0	0,02	100,4933631	uM
2	DUB-IN-2	Bortezomib	KMS-18 T21A	6,25	0,02	99,38133835	uM
2	DUB-IN-2	Bortezomib	KMS-18 T21A	12,5	0,02	94,94890168	uM
2	DUB-IN-2	Bortezomib	KMS-18 T21A	25	0,02	73,58549669	uM
3	DUB-IN-2	Bortezomib	KMS-27	0	0	100	uM
3	DUB-IN-2	Bortezomib	KMS-27	6,25	0	96,52957578	uM
3	DUB-IN-2	Bortezomib	KMS-27	12,5	0	93,75622386	uM
3	DUB-IN-2	Bortezomib	KMS-27	25	0	93,25831508	uM
3	DUB-IN-2	Bortezomib	KMS-27	0	0,005	102,2605059	uM
3	DUB-IN-2	Bortezomib	KMS-27	6,25	0,005	101,1302529	uM
3	DUB-IN-2	Bortezomib	KMS-27	12,5	0,005	99,26807409	uM
3	DUB-IN-2	Bortezomib	KMS-27	25	0,005	81,5475005	uM
3	DUB-IN-2	Bortezomib	KMS-27	0	0,01	90,05178251	uM
3	DUB-IN-2	Bortezomib	KMS-27	6,25	0,01	82,08524198	uM
3	DUB-IN-2	Bortezomib	KMS-27	12,5	0,01	70,61342362	uM
3	DUB-IN-2	Bortezomib	KMS-27	25	0,01	26,03067118	uM
3	DUB-IN-2	Bortezomib	KMS-27	0	0,02	41,61521609	uM
3	DUB-IN-2	Bortezomib	KMS-27	6,25	0,02	31,70683131	uM
3	DUB-IN-2	Bortezomib	KMS-27	12,5	0,02	22,10714997	uM
3	DUB-IN-2	Bortezomib	KMS-27	25	0,02	6,846245768	uM
4	DUB-IN-2	Bortezomib	KMS-27 A49V	0	0	100	uM
4	DUB-IN-2	Bortezomib	KMS-27 A49V	6,25	0	98,53880549	uM
4	DUB-IN-2	Bortezomib	KMS-27 A49V	12,5	0	97,9758863	uM
4	DUB-IN-2	Bortezomib	KMS-27 A49V	25	0	94,08735228	uM
4	DUB-IN-2	Bortezomib	KMS-27 A49V	0	0,005	108,6154583	uM
4	DUB-IN-2	Bortezomib	KMS-27 A49V	6,25	0,005	99,97205366	uM
4	DUB-IN-2	Bortezomib	KMS-27 A49V	12,5	0,005	96,86202491	uM
4	DUB-IN-2	Bortezomib	KMS-27 A49V	25	0,005	83,38390291	uM
4	DUB-IN-2	Bortezomib	KMS-27 A49V	0	0,01	106,9905781	uM

4	DUB-IN-2	Bortezomib	KMS-27 A49V	6,25	0,01	97,78425423	uM
4	DUB-IN-2	Bortezomib	KMS-27 A49V	12,5	0,01	93,1691153	uM
4	DUB-IN-2	Bortezomib	KMS-27 A49V	25	0,01	49,04183967	uM
4	DUB-IN-2	Bortezomib	KMS-27 A49V	0	0,02	87,48003833	uM
4	DUB-IN-2	Bortezomib	KMS-27 A49V	6,25	0,02	77,09198339	uM
4	DUB-IN-2	Bortezomib	KMS-27 A49V	12,5	0,02	64,2246886	uM
4	DUB-IN-2	Bortezomib	KMS-27 A49V	25	0,02	24,14963271	uM

Table S3

block_id	drug1	drug2	cell_line_name	conc1	conc2	response	conc
1	DUB-IN-2	Carfilzomib	KMS-18	0	0	100	uM
1	DUB-IN-2	Carfilzomib	KMS-18	6,25	0	101,9337487	uM
1	DUB-IN-2	Carfilzomib	KMS-18	12,5	0	97,46706159	uM
1	DUB-IN-2	Carfilzomib	KMS-18	25	0	96,46614238	uM
1	DUB-IN-2	Carfilzomib	KMS-18	0	0,003	79,51179655	uM
1	DUB-IN-2	Carfilzomib	KMS-18	6,25	0,003	75,79069213	uM
1	DUB-IN-2	Carfilzomib	KMS-18	12,5	0,003	70,11200763	uM
1	DUB-IN-2	Carfilzomib	KMS-18	25	0,003	32,29156028	uM
1	DUB-IN-2	Carfilzomib	KMS-18	0	0,006	31,42341606	uM
1	DUB-IN-2	Carfilzomib	KMS-18	6,25	0,006	26,69458346	uM
1	DUB-IN-2	Carfilzomib	KMS-18	12,5	0,006	20,32819256	uM
1	DUB-IN-2	Carfilzomib	KMS-18	25	0,006	3,380655704	uM
1	DUB-IN-2	Carfilzomib	KMS-18	0	0,012	5,232696694	uM
1	DUB-IN-2	Carfilzomib	KMS-18	6,25	0,012	4,456473632	uM
1	DUB-IN-2	Carfilzomib	KMS-18	12,5	0,012	3,285330065	uM
1	DUB-IN-2	Carfilzomib	KMS-18	25	0,012	2,073332652	uM
2	DUB-IN-2	Carfilzomib	KMS-18 T21A	0	0	100	uM
2	DUB-IN-2	Carfilzomib	KMS-18 T21A	6,25	0	103,2342692	uM
2	DUB-IN-2	Carfilzomib	KMS-18 T21A	12,5	0	101,4996672	uM
2	DUB-IN-2	Carfilzomib	KMS-18 T21A	25	0	96,42507537	uM
2	DUB-IN-2	Carfilzomib	KMS-18 T21A	0	0,003	84,30635499	uM
2	DUB-IN-2	Carfilzomib	KMS-18 T21A	6,25	0,003	75,79388386	uM
2	DUB-IN-2	Carfilzomib	KMS-18 T21A	12,5	0,003	66,3260112	uM
2	DUB-IN-2	Carfilzomib	KMS-18 T21A	25	0,003	18,55984964	uM
2	DUB-IN-2	Carfilzomib	KMS-18 T21A	0	0,006	33,56826814	uM
2	DUB-IN-2	Carfilzomib	KMS-18 T21A	6,25	0,006	27,69881358	uM
2	DUB-IN-2	Carfilzomib	KMS-18 T21A	12,5	0,006	18,77520655	uM
2	DUB-IN-2	Carfilzomib	KMS-18 T21A	25	0,006	5,47006539	uM

2	DUB-IN-2	Carfilzomib	KMS-18 T21A	0	0,012	4,506832687	uM
2	DUB-IN-2	Carfilzomib	KMS-18 T21A	6,25	0,012	3,492697443	uM
2	DUB-IN-2	Carfilzomib	KMS-18 T21A	12,5	0,012	2,623438662	uM
2	DUB-IN-2	Carfilzomib	KMS-18 T21A	25	0,012	2,627354243	uM
3	DUB-IN-2	Carfilzomib	KMS-27	0	0	100	uM
3	DUB-IN-2	Carfilzomib	KMS-27	6,25	0	96,52957578	uM
3	DUB-IN-2	Carfilzomib	KMS-27	12,5	0	93,75622386	uM
3	DUB-IN-2	Carfilzomib	KMS-27	25	0	93,25831508	uM
3	DUB-IN-2	Carfilzomib	KMS-27	0	0,003	15,25094603	uM
3	DUB-IN-2	Carfilzomib	KMS-27	6,25	0,003	9,300936069	uM
3	DUB-IN-2	Carfilzomib	KMS-27	12,5	0,003	6,851224856	uM
3	DUB-IN-2	Carfilzomib	KMS-27	25	0,003	2,315275841	uM
3	DUB-IN-2	Carfilzomib	KMS-27	0	0,006	4,057956582	uM
3	DUB-IN-2	Carfilzomib	KMS-27	6,25	0,006	3,370842462	uM
3	DUB-IN-2	Carfilzomib	KMS-27	12,5	0,006	2,843059152	uM
3	DUB-IN-2	Carfilzomib	KMS-27	25	0,006	2,29535949	uM
3	DUB-IN-2	Carfilzomib	KMS-27	0	0,012	3,694483171	uM
3	DUB-IN-2	Carfilzomib	KMS-27	6,25	0,012	2,574188409	uM
3	DUB-IN-2	Carfilzomib	KMS-27	12,5	0,012	2,190798646	uM
3	DUB-IN-2	Carfilzomib	KMS-27	25	0,012	2,325234017	uM
4	DUB-IN-2	Carfilzomib	KMS-27 A49V	0	0	100	uM
4	DUB-IN-2	Carfilzomib	KMS-27 A49V	6,25	0	98,53880549	uM
4	DUB-IN-2	Carfilzomib	KMS-27 A49V	12,5	0	97,9758863	uM
4	DUB-IN-2	Carfilzomib	KMS-27 A49V	25	0	94,08735228	uM
4	DUB-IN-2	Carfilzomib	KMS-27 A49V	0	0,003	74,50894283	uM
4	DUB-IN-2	Carfilzomib	KMS-27 A49V	6,25	0,003	50,16767806	uM
4	DUB-IN-2	Carfilzomib	KMS-27 A49V	12,5	0,003	37,04487384	uM
4	DUB-IN-2	Carfilzomib	KMS-27 A49V	25	0,003	12,13669754	uM
4	DUB-IN-2	Carfilzomib	KMS-27 A49V	0	0,006	19,39076972	uM

4	DUB-IN-2	Carfilzomib	KMS-27 A49V	6,25	0,006	12,1087512	uM
4	DUB-IN-2	Carfilzomib	KMS-27 A49V	12,5	0,006	9,0386458	uM
4	DUB-IN-2	Carfilzomib	KMS-27 A49V	25	0,006	3,245768125	uM
4	DUB-IN-2	Carfilzomib	KMS-27 A49V	0	0,012	5,621207282	uM
4	DUB-IN-2	Carfilzomib	KMS-27 A49V	6,25	0,012	4,687000958	uM
4	DUB-IN-2	Carfilzomib	KMS-27 A49V	12,5	0,012	3,046151389	uM
4	DUB-IN-2	Carfilzomib	KMS-27 A49V	25	0,012	2,175822421	uM

Table S4

Cell line	block_id	drug1	drug2	ZIP_synergy	P_synergy_p_valu	HSA_synergy	HSA_synergy_p_valu	Loewe_synergy	oewe_synergy_p_valu	Bliss_synergy	synergy_p_value
KMS-18	1	DUB-IN-2	Bortezomib	13,89815229	1.97e-02	14,67296119	2.15e-02	13,88622894	3.03e-02	14,32094046	1.63e-02
KMS-18T21A	2	DUB-IN-2	Bortezomib	6,195956995	2.56e-02	4,388060091	1.09e-01	3,73436073	1.82e-01	7,437259602	1.03e-02
KMS-27	3	DUB-IN-2	Bortezomib	15,90375521	3.51e-02	17,46774658	4.02e-02	11,73508108	2.69e-02	15,77247559	3.83e-02
KMS-27A49A	4	DUB-IN-2	Bortezomib	19,93198583	1.82e-02	17,5516342	4.76e-02	13,62974598	8.28e-02	21,67720368	1.04e-02

Table S5

Cell Line	block_id	drug1	drug2	ZIP_synergy	ZIP_synergy_p_value	HSA_synergy	HSA_synergy_p_value	Loewe_synergy	Loewe_synergy_p_value	Bliss_synergy	Bliss_synergy_p_value
KMS-18	1	DUB-IN-2	Carfilzomib	11,50474663	4.64e-02	12,2323222	4.58e-02	12,35271278	4.45e-02	11,69884723	4.25e-02
KMS-18T21A	2	DUB-IN-2	Carfilzomib	16,05555914	3.96e-02	16,19744965	4.64e-02	16,09420062	4.81e-02	16,35505136	3.59e-02
KMS-27	3	DUB-IN-2	Carfilzomib	-0,328283744	8.74e-01	3,882582044	2.68e-02	2,492288378	2.87e-02	3,461980847	3.08e-02
KMS-27A49V	4	DUB-IN-2	Carfilzomib	17,29320695	2.60e-02	18,32348557	2.69e-02	13,29233472	4.08e-02	17,28427089	2.60e-02

Table S6. Clinical characteristics of MM patients from whom samples were derived.

patient ID	bortezomib resistant/ refractory*	gender	age	BM sampling	number of prior therapy lines	prior therapy regimens**	details
MM_01	yes	female	40	at initial diagnosis	0	n.a.	insufficient response upon VCD induction therapy, switch to VRD/ Dara-Rd
MM_02	yes	male	62	at initial diagnosis	0	n.a.	insufficient response upon VCD induction therapy, switch to Dara-VTD
MM_03	yes	female	53	at initial diagnosis	0	n.a.	insufficient response upon BAD induction therapy, switch to Dara-Rd
MM_04	yes	male	67	at initial diagnosis	0	n.a.	insufficient response upon BAD induction therapy, switch to Dara-Rd
MM_05	yes	male	82	at initial diagnosis	0	n.a.	progressive disease upon 4 cycles of VD
MM_06	yes	male	52	during treatment	1	VRD	insufficient response upon VCD induction therapy, switch to VRD
MM_07	yes	female	50	upon alloSCT	2	VCD + autoSCT; Dara-VD + alloSCT	recurrent myeloma d+315 upon alloSCT, received prior bortezomib-containing treatment regimens
MM_08	yes	female	66	upon alloSCT	2	VCD + autoSCT; Len/Pom-AD + alloSCT	myeloma d+45 upon alloSCT, received prior bortezomib-containing treatment regimens
MM_09	yes	male	67	during treatment	6	Z-Dex + autoSCT; VD; Rd +	refractory myeloma upon 30 cycles of Isa-

						alloSCT; Dara-Rd; KD; Isa-Pom-Dex	Pom-Dex, received prior bortezomib-containing treatment regimens
MM_10	no	female	71	at initial diagnosis	0	n.a.	achieved PR before auto-SCT upon 4 cycles VCD and mobilization chemotherapy with CE
MM_11	no	male	72	at initial diagnosis	0	n.a.	achieved PR before auto-SCT upon 4 cycles VCD and mobilization chemotherapy with CE
MM_12	no	male	66	at initial diagnosis	0	n.a.	achieved PR before auto-SCT upon 4 cycles VCD and mobilization chemotherapy with CE
MM_13	no	male	62	at initial diagnosis	0	n.a.	achieved PR before auto-SCT upon 4 cycles VCD and mobilization chemotherapy with IEV
MM_14	no	male	74	at initial diagnosis	0	n.a.	achieved a VGPR upon 6 cycles VCD, subsequently de-escalation to VD
MM_15	no	female	63	at initial diagnosis	0	n.a.	achieved a VGPR upon 7 cycles VCD/BAD, subsequently de-escalation to VD

** Patients were either treatment-naive and had an insufficient response to bortezomib-containing induction treatment regimens (resistant) or their myeloma progressed upon bortezomib-containing treatment regimens (refractory)*

*** Treatment regimens: VCD – Bortezomib/ Cyclophosphamide/ Dexamethasone; BAD - Bortezomib/ Adriamycin/ Dexamethasone; Z-Dex – Idarubicin/ Dexamethasone; VRD – Bortezomib/ Lenalidomide/ Dexamethasone; (Dara)-Rd – (Daratumumab)/ Lenalidomide/ Dexamethasone; Dara-VTD – Daratumumab/ Bortezomib/ Thalidomide/ Dexamethasone; (Dara)-VD – Daratumumab/ Bortezomib/ Dexamethasone; Len/Pom-AD – Lenalidomide/ Pomalidomide/ Adriamycin/ Dexamethasone; KD – Carfilzomib/ Dexamethasone; Isa-Pom-Dex – Isatuximab/ Pomalidomide/ Dexamethasone; CE – Cyclophosphamide/ Etoposide; IEV –Iifosfamide/ Epirubicin/ Etoposide; autoSCT – autologous stem cell transplantation; alloSCT – allogenic stem cell transplantation*

Table S7 Bliss Score (CI)

	KMS-18	KMS-18T21A	KM-S27	KMS-27A49V
24 h	0,99396991	0,71384125	0,99757824	0,95268967
48 h	0,98379361	0,65171124	0,94665343	0,95085692

$CI = (E_A+E_B-E_A*E_B)/E_{AB}$

E_A= Effect of drug A

E_B=Effect of drug B

E_{AB}=Effect of combination of Drug A and B

(0,9<CI<1,1 is considered additive; 0,7<CI<0,9 is considered mildly synergistic)

Table S8. Antibodies/target-specific binding molecule list.

Experiment	Marker/target-specific binding molecule	Clone ID	Source
**Histology**	B220	RA3-6B2	BD Pharmingen
	CD3	CD3-12	Serotec
	IgD (biotinylated)	11.26.c.2a	BioLegend
	IgM	Polyclonal	Chemicon
	Moma1	MCA947	AbD Serotec
	Biotinylated peanut agglutinin (PNA)	# B-1075	Vector Laboratories
	Ki67	TEC-3	DAKO
**FACS**	Anti-CD16/CD32 (Fc Block™)	2.4G2	BD Pharmingen
	CD3ε	500A2	eBioscience
	CD19	MB19-1	eBioscience
	B220	RA3-6B2	BD Pharmingen
	IgD	11.26.c.2a	BD Pharmingen
	IgM	II/41	eBioscience
	CD5	53-7.3	BD Pharmingen
	CD21	7G6	BD Pharmingen
	CD23	B3B4	BD Pharmingen
	CD43	S7	BD Pharmingen
	CD93	AA4.1	eBioscience
	Ki67	B56	BD Pharmingen
	T- and B-cell activation antigen	GL7	BD Pharmingen
	Fas	Jo2	BD Pharmingen
	CD138	281-2	BioLegend
	IRF4	REA201	Milteny
	IgG1	RMG1-1	BioLegend
	Blimp1	5E7	BioLegend
	IgM	RMM-1	BioLegend
	CD16/32	93	BioLegend
	CD19	6D5	BioLegend
	CD21/CD35	7E9	BioLegend
	CD25	3C7	BioLegend
	CD3	17A2	BioLegend
	CD335	29A1.4	BioLegend
	CD4	GK1.5	BD Pharmingen
	CD44	IM7	BioLegend
	CD62L	MEL-14	BioLegend
	CD8a	53-6.7	BioLegend
	F4/80	BM8	BioLegend
	FOXP3	150D	BioLegend
	I-A/I-E	M5/114.15.2	BioLegend
	IgM	RMM-1	BioLegend

	Ly-6C	HK1.4	BioLegend
	Ly6G	1A8	BD Pharmingen
	TCRbeta	H57-597	BioLegend
**WB**	USP8(13aa)	Polyclonal	[37]
	USP8 (X39)	Polyclonal	[87]
	α,β,γ Actin	I-19	Santa Cruz Biotechnology
	FLAG	M2	Sigma
	HA	3F10	Roche
	Ubiquitin	P4D1	Santa Cruz Biotechnology
	NEDD8	Polyclonal, #2745	Cell Signaling Technology
	NEDD8	Y297	Abcam
	phospho-p44/42	197G2	Cell Signaling Technology
	phospho-JNK	81E11	Cell Signaling Technology
	phospho-p38	3D7	Cell Signaling Technology
	phospho-Akt	Polyclonal, #9271	Cell Signaling Technology
	phospho-IκBα	14D4	Cell Signaling Technology
	Roquin1/2	3F12	[76]
	IκBα	Polyclonal, #9242	Cell Signaling Technology
	phosphotyrosine	PY20	BD Biosciences
	DR5	E9D7D	Cell Signaling Technology
	CHOP	L63F7	Cell Signaling Technology
	LC3A/B	Polyclonal, #4108	Cell Signaling Technology
	Caspase3	Polyclonal, #9662	Cell Signaling Technology
	Caspase8	D35G2	Cell Signaling Technology
	Caspase9	C9	Cell Signaling Technology
	XBP1s	E9V3E	Cell Signaling Technology
	SUMO-1	Polyclonal, #4930	Cell Signaling Technology
	SUMO-2/3	18H8	Cell Signaling Technology
	Cullin-1	EPR3103Y	Abcam
	Cullin-3	Polyclonal, #11107-1-AP	Proteintech
	Cullin-4b	Polyclonal, #12916-1-AP	Proteintech
	K48-linkage specific Polyubiquitin	Polyclonal, #4289	Cell Signaling Technology
	K63-linkage specific Polyubiquitin	D7A11	Cell Signaling Technology

Methods

Mice. *Usp8*$^{f/f}$ [1], *Cd19*-Cre (B6.129P2(C)-*Cd19*$^{tm1(cre)Cgn}$/J) [2], *mb1*-Cre (B6.C(Cg)-*Cd79a*$^{tm1(cre)Reth}$/EhobJ) [3], *Rosa26*-CreERT2 (Gt(ROSA)26Sor$^{tm2(cre/ERT2)Brn}$) [4,5], *Rc3h1*$^{fl/fl}$ [6] and *Rc3h1*$^{fl/fl}$ [7] transgenic, and NSG (NOD.Cg-*Prkdc*^{scid} *Il2rg*^{tm1Wjl}/SzJ) [8] mice have been described previously. Mice used for experiments were 8-12 weeks old and on the C57BL/6 background. For inducible deletion, *Rosa26*-Cre-ERt2 mice [5], with or without *Rc3h1/2*$^{fl/fl}$ alleles (encoding Roquin-1 [6] and Roquin-2 [7]), were treated with tamoxifen (Sigma) by oral gavage (5 mg per dose in corn oil) twice daily for two consecutive days (total dose: 20 mg per mouse) and analyzed 3 days after the final treatment. For histology, B-cell enrichment, and paired analyses, sex-matched littermates were used. Animals were housed under specific pathogen-free conditions **Mice.** *Usp8*$^{f/f}$ [1], *Cd19*-Cre (B6.129P2(C)-*Cd19*$^{tm1(cre)Cgn}$/J) [2], *mb1*-Cre (B6.C(Cg)-*Cd79a*$^{tm1(cre)Reth}$/EhobJ) [3], *Rosa26*-CreERT2 (Gt(ROSA)26Sor$^{tm2(cre/ERT2)Brn}$) [4,5], *Rc3h1*$^{fl/fl}$ [6] and *Rc3h1*$^{fl/fl}$ [7] transgenic, and NSG (NOD.Cg-*Prkdc*^{scid} *Il2rg*^{tm1Wjl}/SzJ) [8] mice have been described. Mice used for experiments were 8-12 weeks old and on the C57BL/6 background. *Rosa26*-Cre-ERt2 mice [5] in combination with/without *Rc3h1/2*$^{fl/fl}$ alleles which are transgenes for Roquin-1 [6] and Roquin-2 [7], respectively, were fed by oral gavage with 5 mg tamoxifen (Sigma) in corn oil per dose on two consecutive days with two doses of tamoxifen each day (20 mg total tamoxifen dose per mouse) and mice were killed 3 d after the last gavage. For histology, B-cell enrichment, and if paired T-tests were performed, sex-matched littermates were used. Animals were housed in a specific pathogen-free barrier facility according to the protocols of the Center for Experimental Models and Transgenic Service at the University Clinic in Freiburg, the LMU Munich, or at the RWTH University Aachen. All experiments were approved by national authorities and the ethics review board for animal studies at the University of Freiburg, the LMU Munich, or RWTH University Aachen.

Repeated dose toxicity study of DUB-IN-2 and BTZ. Evaluation of the general toxicity of two compounds, DUB-IN-2 (5 mg/kg, 5x per week IP) alone, and in combination with BTZ (0.6 mg/kg, 2x per week IP) was carried out by BIENTA, Enamine Biology Services, Ukraine. 11-12 weeks old male and female C57BL/6J mice were divided into three groups per sex consisting of five C57BL/6J mice each. The mice were repeatedly IP dosed with vehicle (PEG300 – saline, 50%:50%, v/v), test compound DUB-IN-2 (5x per week) in vehicle or combination of the test compounds DUB-IN-2 (5x per week) and BTZ (2x per week) for 22 consecutive days. Animals were weighed on the day of randomization and every day during the study (from 9 am to 10 am). Observations for mortality were performed once a day during the dosing period. The toxic effects of compounds were evaluated during the study (22 days) by visual observations according to the standard procedures and FELASA recommendations. Autopsy with gross pathology examination was performed on Day 22 of the study. All experimental animal procedures performed were approved by the local Institutional Animal Care and Use Committee of BIENTA LTD (BACUC, Approval number: EN-TOX-AP-211124) and in adherence with the European Convention for the Protection of Vertebrate Animals used for Experimental and other Scientific Purposes.

Southern blot analysis. LoxP-flanked and deleted *Usp8* alleles were identified by Southern blot analysis of NcoI fragments of genomic DNA as described[1].

Cell culture, proliferation, activation, and viability. For the proliferation experiments, purified B cells ($5x10^4$ per well) were stimulated by incubation with 5 µg ml^{-1} anti-IgM $F(ab')_2$ fragment antibody (115-006-020; Jackson Immuno Research) with or without 5 µg ml^{-1} anti-CD40 antibody (553787, BD Pharmingen), with 100 ng/ml MPL-A (Invivogen) or 100 nM CpG (ODN 1668, Invivogen). B-cell proliferation was quantified after 36 h using the Cell Proliferation ELISA, BrdU (colorimetric) from Roche. For short-term stimulation, B cells were

starved for 2 h in RPMI 1640 medium containing 0.2% bovine serum albumin without FCS and activated by preincubation with 10 µg ml^{-1} anti-IgM for 30 min on ice and stimulation for the times indicated at 37°C. T cells were starved for 2 h in RPMI 1640 medium without FCS. T cells were activated by pre-incubation on ice for 30 min with soluble anti-mouse CD3ε antibody (10µg ml^{-1}; 145-2C11; eBioscience) and anti-mouse CD28 antibody (2µg ml^{-1}; 37.51, eBioscience), followed by stimulation for the appropriate time at 37°C with pre-warmed anti-hamster IgG1 antibody (5µg ml^{-1}; G94-56, BD Pharmingen). *Ex vivo* expansion of B cells was performed using recombinant murine IL-4 (100 u ml^{-1}, Peprotech) and anti-mouse CD40 antibody (5 µg ml^{-1}; HM40-3, Biolegend), or anti-IgM F(ab')$_2$ fragment (5 µg ml^{-1}) and anti-CD40 antibody (2.5 µg ml^{-1}). OHT (1 µM, Sigma) was added 24 h post B-cell isolation and stimulation. DUB-IN-2 (25 µM, MedChemExpress), BTZ (5 to 25 nM, Selleckchem), CFZ (3 nM, MedChemExpress), and OTUB1/USP8 inhibitor 61 (Aobious) were added for the times indicated before analysis. Cell viability was analyzed using the XTT cell proliferation kit II (Roche Diagnostics). Absorbance was measured as indicated and normalized to the respective controls.

Effect of inhibitors on MM cells of patients.

BM cells from MM patients were prepared from the pelvic crest. CD138+ MM cells were isolated from the BM using CD138 microbeads (Miltenyi Biotech, Bergisch Gladbach, Germany). The MM cell purity was >90%. Isolated MM cells were co-incubated with solvent (DMSO), Bortezomib 5nM;. DUB-IN-2 25µM or Bortezomib + DUB-IN-2 for 24 hours. DMSO concentrations were adjusted to match across all samples. Negative control incubations contained 0.1% (v/v) DMSO in the respective incubation buffer. Viability of MM cells was measured by AnnexinV and 7AAD staining (BD) according to the manufacturer’s instructions (FACS Canto II, BD).

In some experiments, isolated MM cells were stained with CytoLight Rapid Green (Sartorius, Göttingen, Germany) and seeded in a 96 well plate. Cells were treated with the inhibitors described above and the culture medium was supplemented with Annexin V Red Dye (Sartorius, Göttingen, Germany) to facilitate ongoing staining of apoptotic cells. Green and red fluorescence channels were recorded once every 120 min over a period of 48 hours. The aggregated area of red cells (μm^2/image) as a measure of cell death was automatically quantified by Incucyte Base Software (Sartorius, Göttingen, Germany).

BM cells were collected and used from left-over material of myeloma patients upon diagnostic bone marrow aspiration, which was approved by the local ethics committee of the University of Erlangen (219_14B, 19-56D).

Single cell RNA-seq analysis. Single cell RNA-seq analysis was performed with splenic B cells enriched from *Usp8*$^{f/f}$ and *Usp8*$^{f/f}$*Cd19*-Cre mice using the BD IMag™ Biotinylated Mouse B Lymphocyte Enrichment Cocktail and BD IMAG™ Streptavidin Particles Plus (n=3). A total of 13.303 cells were analyzed. For single cell library preparation, cells were loaded onto a Chromium Single Cell 3′ G Chip (10X Genomics) according to the manufacturer's instructions without modifications using Chromium Next GEM Single Cell 3' Reagents Kit v3.1 (10x Genomics). The 6 samples were multiplexed using Cell Multiplex Oligos (CMOs) for CellPlex reagents. Dual Index Plate NN Set A (10x Genomics) was used for Cell Multiplexing library construction, Dual Index Plate TT Set A was used for Gene Expression library construction according to the manufacturer's instructions. The cDNA content and size of post-sample index PCR samples was analyzed using a 2100 BioAnalyzer (Agilent). Library quantification was done using NEB Next® Library Quant Kit for Illumina® (New England Biolabs) following manufacturer's instructions. Sequencing libraries were loaded onto an Illumina NextSeq 1000 P2 flow cell, with sequencing settings according to the recommendations of 10x Genomics (read 1: 28 cycles, read 2: 90 cycles, i7 index: 10 cycles, i5

index: 10 cycles). Cell Ranger v6.1.2 software was used to align the sequencing data to the mouse genome mm10. The indexed mouse genome used for alignment was downloaded from 10x genomics (refdata-gex-mm10-2020-A).

The single cell data were loaded into R and into a Seurat object (v.4) for subsequent bioinformatics analysis. Cells with a high mitochondrial content (> 20%) or low number of genes detected (<200) were discarded. SoupX[9] was used to estimate and remove cells contaminated from ambient mRNA and ALRA[10] was used for data imputation.

The data were normalized with the "LogNormalize" method and scaled. The top 10.000 variable genes were used for the Principal Component Analysis and the UMAP was generated using the top 20 principal components. The Louvain algorithm with resolution=0.8 from the FindClusters function was used to assign clusters to cells in an unbiased way. Cell type annotation was performed using the markers reported by Nguyen et al.[11] and clusters were merged when no relevant difference in marker expression was detected. Other bioinformatics analyses and data visualization were performed with the other Seurat or ggplot2 functions. The data are available at https://www.ncbi.nlm.nih.gov/geo/query/acc.cgi?acc=GSE244334 (access token for reviewers: **sxsdsecavdyxbqj**). Pathway enrichment analysis was performed with ReactomePA v1.46.0 (10.1039/c5mb00663e). Genes with adjusted p value < 0.1 and log2FC > 0 or <0 were selected to perform the enrichment analysis for pathways upregulated in the *Usp8*$^{f/f}$*Cd19* B cell/PC and control samples, respectively. Pathways that had 70% of overlapping genes were removed from the final results in order to remove redundancy.

Immunoblot analysis and immunoprecipitation. Cells were lysed in DDM lysis buffer (50 mM Tris-HCl, pH 7.4, 150 mM NaCl, 1 mM EDTA, 50 mM NaF, 1mM sodium vanadate, 1x Roche Complete Protease Inhibitor Cocktail, and 0.2% n-dodecyl-β-D-maltoside), or directly in 4x Laemmli buffer supplemented with 6 M Urea. Immunoprecipitation was performed in DDM lysis buffer with anti-FLAG M2 Affinity Gel (Sigma) or rat monoclonal antibody to

hemagglutinin (HA) affinity matrix (3F10; Roche). Immunoblot analysis was performed with antibodies listed in Supplementary Table S8. Quantification of WB band intensities was performed by measuring the integrated density using Adobe Photoshop software.

Conjugation and deconjugation assays. For the PRG probe conjugation assay, protein lysate was diluted 1:8 in 25 µl assay buffer (50 mM Na_2HPO_4, pH7.9, 500 mM NaCl, 10mM DTT) containing 2 µg NEDD8-PRG or 5µM Bt-Ub-PRG and incubated for 2 h at 37°C. To test the deconjugation capacity of USP8 towards mixed ubiquitin/NEDD8 chains, 5µl protein lysate generated with DDM lysis buffer w/o proteinase inhibitors was incubated with 365 ng recombinant USP8 in PBS containing 5mM $MgCl_2$ and 2mM DTT for 1 h at 37°C.

DUB activity analysis, inhibitor testing, and production of recombinant USP8. The selectivity of DUB-IN-2 was evaluated in the DUB*profiler* assay (Ubiquigent) against 48 individual deubiquitinase enzymes, at concentrations of 0.03, 0.1, 0.3, 1, 3, 10, 30, and 100 μM. Values in the presence of compound were compared with those of DMSO controls to give the percentage of remaining activity. DUB-IN-2 did not show any significant autofluorescent properties in the ubiquitin-rhodamine assay up to 100 μM.

Bacterially eypressed full length USP8 was a gift from Emma Murphy (Alzheimer's Research UK Oxford Drug Discovery Institute, University of Oxford). WT and catalytically inactive His6-tagged USP8 were expressed in Sf21 cells using the MultiBac system[12]. In brief, a His6-tag followed by the HRV 3C recognition site was amplified from the vector pKL-His-3C-USP18[13] (BamHI-His-fwd: AAAGGGATCCATGGGTCACCATCACCACC; His-EcoRI-rev: AAAGAATTCACCCGGACCCTGGAACA) and ligated into pFL vector[14] yielding vector pFL-His-3C. Human USP8 cDNA was amplified (USP8_SalI_FL_fwd: AAAGTCGACGAATGCCTGCTGTGG; USP8_HindIII_FL_rev: AAAAAGCTTTTATGTGGCTACATCAGTTACT) and ligated into the pFL-His3C vector.

Constructs for USP8C786R expression were generated using QuikChange site directed mutagenesis (Agilent; USP8C786R_f: CTTAGGAAATACTCGTTATATGAACTC; USP8C786R_rev: GAGTTCA TATAACGAGTATTTCCTAAG). Constructs were transformed into competent E. coli harboring the MultiBac bacmid[14] via electroporation. Sf21 insect cells were transfected with transposed bacmid DNA using X-tremeGene HP DNA transfection reagent (Roche). Primary virus (V0) was harvested after 2–3 days and used to generate secondary virus (V1) by infecting cells at a density of 0.5×10^6 cells/mL. Infected cells were monitored for fluorescence, harvested after 5–7 days, and protein expression was confirmed by SDS-PAGE and WB analysis.

For batch purification of USP8 expressed in insect cells, cell pellets were thawed on ice and resuspended in lysis buffer (20 mM Tris, pH 8.0, 1 M NaCl, 1% Nonidet P-40, 10 mM DTT, and 25 mM imidazole). The suspension was incubated on ice for 10 minutes and subsequently sonicated to facilitate cell lysis. The lysate was centrifuged at 10,000 × g for 10 minutes at 4°C to separate cellular debris and DNA. The cleared lysate was then combined with pre-washed INDIGO-Ni agarose resin (Cube Biotech) and incubated with gentle rotation at 4°C for 3 hours to allow binding of His-tagged proteins. The flow-through was discarded, and the resin was washed sequentially with buffers containing increasing concentrations of imidazole (20 mM Tris, pH 8.0, 1 M NaCl, 10 mM DTT, 40–100 mM imidazole) to remove non-specifically bound proteins. Finally, the His-tagged proteins were eluted by incubating the resin with elution buffer (20 mM Tris, pH 8.0, 300 mM NaCl, 10 mM DTT, and 500 mM imidazole) at room temperature for 10 minutes. DUB activity assays with bacterially expressed human FL USP8 (30 nM) or human USP8 expressed in insect cells (75 nM) were performed in assay buffer (20 mM HEPES pH 7.4, 150 mM KCl, 10 mM NaCl, 5% glycerol, 0.2 mg/ml BSA, 5mM DTT (freshly added)) using ubiquitin-rhodamine-morpholine (100 nM) as a substrate. Fluorescence intensity was

measured over a period of 1 hour using a Spark® TECAN Reader with an excitation wavelength of 480 nm and an emission wavelength of 520 nm, controlled by Spark Control software v3.2.

Proteasome activity testing. Proteasome activity was evaluated using fluorogenic substrates specific for chymotrypsin-like (Suc-LLVY-AMC), trypsin-like (Boc-LRR-AMC), and caspase-like (Z-LLE-AMC) activities. The assay was performed with the Proteasome Activity Fluorometric Assay Kit (UBPBio) following the manufacturer's instructions.

ELISA, T cell-dependent and -independent Ig responses, cytokine bead array. Basal immunoglobulin levels in mouse plasma were analyzed by Aushon Biosystems, Billerica, MA. *Usp8*$^{f/f}$ and *Usp8*$^{f/f}$*Cd19*-Cre mice have been i.p. injected with 50 µg TNP-KLH (Biosearch Technologies) precipitated in alum (TD response) or with 25 µg TNP-Ficoll (Biosearch Technologies, TI response) and blood was collected at day 0 and days 7, 14, and 28 post injection. TNP-specific IgM, IgG1 and IgG3 levels in the plasma were quantified using the SBA Clonotyping™ System/AP from SouthernBiotech. Detection of serum cytokine levels was performed using Mouse Enhanced Sensitivity Master buffer Kit (562246, Becton Dickinson) along with Flex Kits for TNF (562336), IL-6 (562236) and IL-1beta (562278) according to manufacturer's recommendations.

Ca^{2+} flux. B cells were purified, starved of serum as described above and loaded for 30 min at 37°C with Indo-1-AM (5µg/ml) in RPMI 1640 medium without FCS. Stimulation with anti-IgM (5µg/ml; identified above) was carried out in RPMI-1640 medium with 10% FCS. The emission wavelength ratios of intracellular Ca^{2+}-bound and unbound Indo-1-AM were analyzed on a LSRII or a BD Fortessa SORP flow cytometer with FACSDiva software (BD Biosciences).

Immunohistochemistry and acridine orange (AO) staining. Hematoxilin and eosin staining and immune histological staining of splenic paraffin or cryosections with antibodies was carried out with the appropriate antibodies (dilution 1:500, except for CD3: 1:100; anti-B220: 1:200; Ki67: 1:50; PNA: 1:250) (Supplementary Table S8) as described previously[15,16]. In brief, spleens were removed and fixed in 4 % buffered formalin. Paraffin-embedded 3 μm sections were deparaffinized and were incubated in 3 % H_2O_2 in methanol and subsequently in 70 % ethanol. Cryosections were fixed for 5 min in ice-cold acetone, followed by incubation with 10% FCS in PBS. Spleen sections were incubated with the primary antibodies overnight. Secondary antibodies and fluorescence labelled streptavidin (1:500) were added for 2 h at room temperature. Images were taken using a conventional fluorescence microscope (Olympus BX-61). Cells were stained in AO (100 ng/ml, Invitrogen) according to manufacturer's instructions. Live cell imaging was performed at 37°C in a 5% CO2 humidified atmosphere with a Leica SP8 confocal microscope equipped with a 63×/1.40 and 40×/1.25 oil objective (Leica Microsystems) or a ZEISS Celldiscoverer 7 confocal microscope (Lighthouse Core Facility). Excitation was performed using the 488 nm laser; emission was recorded simultaneously in both the GFP range (for nucleic acids) and the Cy5/Alexa647 range (for protonated AO trapped in organelles). Laser settings were kept constant for each experiment to allow direct comparison of signal intensities between images of the same channel. LAS X software (version 3.7.3.23245) was used for confocal imaging with the Leica TCS SP8 X. ZEN 3.4 (Blue Edition) software was used for confocal imaging with the ZEISS Celldiscoverer 7 confocal microscope.

Generation of stable GFP expressing cell lines

KMS-18 and KMS-18-T21A cell lines were seeded at $1x10^5$ in 200µl medium (RPMI 1640 supplemented with 10% Fetal Bovine Serum, without antibiotics) and mixed with polybrene (16µg/ml, Sigma-Aldrich). Cells were then placed in a 48-well plate (Thermo Fisher Scientific) together with the virus particles (Firefly luciferase-GFP lentivirus, System Biosciences, stock:

21,9 TU/ml, MOI 10). The plate was centrifuged for 2h at 1200g and 32°C. After centrifugation, cells were expanded for 5 days (37 °C and 5% CO2) in the presence of puromycin (2 µg/ml) and the GFP positive cells were isolated by flow cytometry.

Transplantation of NSG mice

$1x10^6$ MM KMS-18 or KMS-18-T21A cells, combined with $5x10^5$ HS27a human stromal cells were transplanted into 9-week-old NOD/Scid/IL2Rγ-/- (NSG) mice (Charles River) after sublethal irradiation with 240 cGy (2,4Gy) using MultiRad 225 (Cu 0,3mm filter). To prevent infections, cotrimoxazol (100 µg/ml) was added to the drinking water for 2 weeks after transplantation. Bortezomib was administered twice per week (i.p.) at a concentration of 0,6mg/kg and DUB-IN-2 solved in 0.9% NaCl/PEG300 1:1 (v/v) was administered 2-5 times per week (i.p.) at concentrations of 2-5 mg/kg. As control we administered 0.9% NaCl/PEG300 1:1 (v/v).

Chemical synthesis of K48-ubiquitinated-NEDD8 and K48-diubiquitin. K48-ubiquitinated-NEDD8 and K48-diubiquitin were chemically synthesised as described by El Oualid et al.[17]. In brief, $Ubiquitin_{1-76}$-thioester and K48-γ-thiolysine- Ub or NEDD8 were prepared using automated solid phase peptide synthesis. The StBu protected K48γ-thiolysine-Ub or NEDD8 (5 mg) was dissolved in 50 µL DMSO and added to 600 µL 8 M Gdn.HCl/100 mM phosphate buffer at pH 7.6 supplemented with 50 µL 1 M TCEP and reacted at 37 ^{0}C. After LC-MS analysis revealed complete deprotection of the thiolysine, 1.25 equivalent of Ub-thioester dissolved in 50 µL DMSO and 50 uL 1M MPAA were added. The pH was re-adjusted to 7.6 and the reaction was performed for 2 hours at 37 ^{0}C. Following RP-HPLC purification the appropriate fractions were lyophilized. The lyophylised diUb(l) (1.5 mg) was desulfurized by dissolving in 25 µL DMSO and subsequent dilution into 600 µL 8 M Gdn.HCl/100 mM phosphate buffer at pH 7.6 and 150 µL 1M TCEP. VA044 (25 mg/mL) and GSH (25 mg/mL)

were added, the pH was adjusted to 7.0 and the reaction was agitated for 16 hours at 37 ^{0}C. Following RP-HPLC purification the appropriate fractions were lyophilized. The lyophilized diUb(l) was dissolved in 25 µL DMSO and diluted into 750 µL 20 mM TRIS, 150 mM NaCl at pH 7.6 and purified on a S75 10/300 sephadex size exclusion column (GE Healthcare). Appropriate fractions were collected and pooled. Spin-filtration (Amicon 3 kD MWCO) was used to concentrate the sample to 3.4 mg/mL, based on nanodrop analysis (M.W.: 17089 Da, ε_{280}: 4470 $M^{-1}cm^{-1)}$. The aliquots were snap frozen and stored at -80 ^{0}C until further use.

HRMS-measurements. High resolution mass spectra were recorded on a Waters Acquity H-class UPLC with XEVO-G2 XS Q-TOF mass spectrometer equipped with an electrospray ion source in positive mode (source voltage 3.0 kV, desolvation gas flow 900 L/hr, temperature 250°C) with resolution R = 22000 (mass range m/z = 50-2000) and 200 pg/uL Leu-Enk (m/z = 556.2771) as a lock mass. Data processing was performed using Waters MassLynx MS Software 4.1 (deconvolution with MaxEnt1 function).

K48-ubiquitinated-NEDD8: HR-MS analysis for $C_{757}H_{1266}N_{208}O_{234}S_{2}$: Deconvoluted ESI MS^{+} (amu) calcd: 17089.5, found 17089.0, rt: 1.35 min.

K48-diubiquitin: HR-MS analysis for $C_{757}H_{1258}N_{210}O_{235}S$: Deconvoluted ESI MS^{+} (amu) calcd: 17093.7, found 17095.0, rt: 1.36 min.

DUB cleavage assays. USP8 (human, full length, Ubiquigent 64-0053-050) was diluted to a final concentration of 40 nM in buffer containing 20 mM Tris-HCl, 150 mM NaCl, pH 7.6 and 5 mM DTT. Subsequently 5 µM synthetic K48-ubiquitinated-NEDD8 or K48-diubiquitin were added. The samples were incubated at 37°C for 30, 60 and 120 minutes. At the indicated time the reaction mixture was diluted with sample buffer (3x) (containing 75% NUPAGE® LDS

sample buffer (4x, Invitrogen), 7.5% β-mercaptoethanol and 17.5% water), and loaded on 12% NUPAGE® Novex® Bis-Tris Mini Gels (Invitrogen) using MES-SDS as running buffer. SeeBlue Pre-stained Standard (Invitrogen, LC5925) was used as molecular weight marker. Gels were stained using InstantBlue™ (Expedeon) and scanned using a GE Healthcare Amersham Imager 600.

Ubiquitylation proteomics.

Sample preparation for pull down-MS experiments.

In the pull down-MS experiments, after immunoprecipitation beads were washed 2 times with DDM lysis buffer and three times with trypsin buffer containing 20 mM Tris HCl, pH 8.0; 2 mM $CaCl_2$. The proteins were on-bead digested with 1 µg trypsin (Promega) for 4 hours at 37 ˚C under agitation. Then, the beads were precipitated and the supernatants were mixed with another 1 µg of trypsin and further digested overnight at 37°C under agitation. Peptides were desalted on reversed phase C18 OMIX tips (Agilent), dried under vacuum in HPLC inserts and stored at −20 °C until LC-MS/MS analysis.

Sample preparation for GlyGly(K) sites identification by MS.

200 x 10^6 K46Lµm cells were homogenized in 10 mL urea lysis buffer containing 9 M urea; 20 mM HEPES, pH 8.0. Samples were sonicated by three pulses of 10 s at an amplitude of 20% and centrifuged for 15 min at 16,000 × g at room temperature to remove insoluble components. The protein concentration in the supernatants of each replicate was measured using a Bradford assay (Biorad) and equal protein amounts, each containing 10 mg total protein, were used for further analysis. Proteins in each sample were reduced by adding 5 mM DTT and incubation for 30 min at 55 °C. Alkylation of the proteins was done by addition of 10 mM chloroacetamide and incubation for 15 min at room temperature in the dark. Next, samples were diluted with 20 mM HEPES, pH 8.0 to an urea concentration of 4 M and proteins were digested with 40 µg endopeptidase LysC (Promega) (1/250, w/w) for 4 h at 37 °C. Samples were further diluted

with 20 mM HEPES pH 8.0 to a urea concentration of 2 M and proteins were digested with 50 µg trypsin (Promega) (1/200, w/w) overnight at 37 °C. Immunocapture of GG-modified peptides was then performed using the PTMScan® Ubiquitin Remnant Motif (K-ε-GG) Kit (Cell Signaling Technology) according to the manufacturer's instructions. Briefly, peptides were purified on Sep-Pak C18 cartridges (Waters), lyophilized for 2 days and re-dissolved in 1.4 ml 1x immunoprecipitation buffer supplied with the kit. At this point, aliquots corresponding to 50 µg of digested protein material were taken for shotgun proteomics analysis. Peptides were incubated with the antibody-bead slurry for 2 h on a rotator at 4 °C and after several wash steps, GG-modified peptides were eluted in 100 µl 0.15% TFA and desalted on reversed phase C18 OMIX tips (Agilent). Purified GlyGly-K modified peptides were dried under vacuum in HPLC inserts and stored at −20 °C until LC-MS/MS analysis.

LC-MS/MS.

GlyGly-K modified peptides isolated from B cells shown in Figure 3A were re-dissolved in 20 µL loading solvent A (0.1% TFA in water/ACN (98:2, v/v)) of which 10 µL were injected for LC-MS/MS analysis on an Ultimate 3000 RSLCnano system (ThermoFisher scientific) in-line connected to a Q Exactive HF mass spectrometer (Thermo). Trapping was performed at 10 µL/min for 4 min in loading solvent A on a 20 mm trapping column (100 µm internal diameter (I.D.), 5 µm beads, C18 Reprosil-HD, Dr. Maisch, Germany). Peptides were separated on a 200 cm long micro pillar array column (µPAC™, PharmaFluidics) with C18-endcapped functionality. This column consists of 300 µm wide channels that are filled with 5 µm porous-shell pillars at an inter pillar distance of 2.5 µm. With a depth of 20 µm, this column has a cross section equivalent to an 85 µm inner diameter capillary column. Peptides isolated from B cells were eluted from the analytical column by a non-linear gradient from 2 to 55% solvent B (0.1% FA in water/acetonitrile (2:8, v/v)) over 120 minutes at a constant flow rate of 300 nL/min, followed by 20 min in 99% solvent B. The column was then re-equilibrated with 98% solvent A for 10 min. For the analyses of peptides isolated from B cells, the column temperature was

kept constant at 50 °C in a column oven (CoControl 3.3.05, Sonation). The mass spectrometer was operated in positive and data-dependent mode, automatically switching between MS and MS/MS acquisition for the 8 most abundant ion peaks per MS spectrum for the analyses of the peptides isolated from B cells. Full-scan MS spectra (375–1,500 m/z) were acquired at a resolution of 60,000 (at 200 m/z) in the orbitrap analyzer after accumulation to a target value of 3E6 for a maximum of 60 ms. The most intense ions above a threshold value of 8.3E3 were isolated in the trap with an isolation window of 1.5 Da for maximum 120 ms to a target AGC value of 1E5 for the analysis of peptides isolated from B cells. Only precursor ions with a charge state from 2 to 6 were selected for MS/MS. Peptide match was set on "preferred" and isotopes were excluded. Dynamic exclusion time was set to 12 s. Fragmentation was performed at a normalized collision energy of 28%. MS/MS spectra (200-2,000 m/z) were acquired at fixed first mass 145 m/z at a resolution of 15,000 (at 200 m/z) in the Orbitrap analyzer. MS/MS spectrum data type was set to centroid. The polydimethylcyclosiloxane background ion at 445.12003 was used for internal calibration (lock mass).

Dried peptides of the shotgun aliquots were re-dissolved in loading solvent A (0.1% TFA in water/ACN (98:2, v/v)) and 3 µg peptides of each sample was injected for LC-MS/MS analysis on an Ultimate 3000 RSLC nanoLC (Thermo Scientific, Bremen, Germany) in-line connected to an LTQ-Orbitrap Elite (Thermo Fisher Scientific, Bremen, Germany) equipped with a pneu-Nimbus dual ion source (Phoenix S&T). Trapping was performed at 10 µL/min for 4 min in solvent A on a 20 mm trapping column (made in-house, 100 µm internal diameter (I.D.), 5 µm beads, C18 Reprosil-HD, Dr. Maisch, Germany) and the sample was loaded on a reverse-phase column (made in-house, 75 µm I.D. x 250 mm length, 1.9 µm beads C18 Reprosil-HD, Dr. Maisch). Peptides were eluted by a non-linear increase from 1 to 56% MS solvent B (0.1% FA in water/ACN (2:8, v/v)) over 145 minutes, at a flow rate of 250 nL/min, followed by a 15-minutes wash reaching 99% MS solvent B and re-equilibration with MS solvent A (0.1% FA in water/ACN (2:8, v/v)). The mass spectrometer was operated in data-dependent, positive

ionization mode, automatically switching between MS and MS/MS acquisition for the 20 most abundant peaks in a given MS spectrum. The source voltage was 3.1 kV, and the capillary temperature was 275°C. In the LTQ-Orbitrap Elite, full scan MS spectra were acquired in the Orbitrap (m/z 300−2,000, AGC target 3 × 10^6 ions, maximum ion injection time 100 ms) with a resolution of 60 000 (at 400 m/z). The 20 most intense ions fulfilling predefined selection criteria (AGC target 5 × 10^3 ions, maximum ion injection time 20 ms, spectrum data type: centroid, exclusion of unassigned and 1 positively charged precursors, dynamic exclusion time 20 s) were then isolated in the linear ion trap and fragmented in the high pressure cell of the ion trap. The CID collision energy was set to 35% and the polydimethylcyclosiloxane background ion at 445.120028 Da was used for internal calibration (lock mass).

For the pulldown-MS analyses shown in Figure 3E, dried peptides were re-dissolved in 20 µl loading solvent A of which 13 µL were injected for LC-MS/MS analysis as described for the analysis of peptides isolated from B cells. Peptides were eluted from the analytical column by a non-linear gradient from 2 to 56% solvent B over 150 minutes at a constant flow rate of 250 nL/min, followed by 10 min in 99% solvent B. The column was then re-equilibrated with 98% solvent A (0.1% FA in water) for 20 min. The column temperature was kept constant at 50 °C in a column oven (CoControl 3.3.05, Sonation). The mass spectrometer was operated as described above with minor modifications. It was run in positive and data-dependent mode, automatically switching between MS and MS/MS acquisition for the 16 most abundant ion peaks per MS spectrum. Full-scan MS spectra (375–1,500 m/z) were acquired at a resolution of 60,000 (at 200 m/z) in the orbitrap analyzer after accumulation to a target value of 3E6 for a maximum of 60 ms. The most intense ions above a threshold value of 1.3E4 were isolated in the trap with an isolation window of 1.5 Da for maximum 80 ms to a target AGC value of 1E5.

For the pull down-MS analyses shown in Figure 3H, dried peptides were re-dissolved in 20 µl loading solvent A of which 5 µL were injected for LC-MS/MS analysis as described above for the analysis of peptides isolated from B cells. Peptides were eluted from the analytical

column as described for the analysis of peptides isolated from B cells, but over a gradient for 90 minutes. The column temperature was kept constant at 50 °C in a column oven (CoControl 3.3.05, Sonation). The mass spectrometer was operated as described above, but only the top 12 most abundant ion peaks were fragmented per MS spectrum. Fragmentation was performed as described for the analysis of peptides isolated from B cells but the dynamic exclusion time was set to 20 s.

LC-MS/MS data analysis.

Data analysis was performed with MaxQuant (version 1.5.7.4, 1.5.8.3, 1.6.1.0 or 1.6.11.0) using the Andromeda search engine with default search settings including a false discovery rate set at 1% on both the peptide and protein level. The analysis of the B cells shown in Figure 3B was performed with two different searches to analyze the spectra generated from the analysis of the GlyGly-K enriched samples and the shotgun samples. Spectra from the pull down-MS shown in Figure 3E were searched twice: for the identification of GlyGly-K modified peptides and for the identification of proteins. Spectra from the pull down-MS shown in Figure 3H were searched once: for the identification of GlyGly-K modified peptides. Spectra were interrogated against proteins in the Uniprot/Swiss-Prot database ([www.uniprot.org]). The database released on January 2018 containing 16,946 mouse protein sequences were used for the analysis of Fig. 3B. The database released on September 2017 or January 2020 containing 20,226 or 20,595 human protein sequences was used for the pulldown-MS shown in Figure 3E and 3H, respectively. Mass tolerance for precursor ions was set to 4.5 ppm for all analyses. Mass tolerance for fragment ions was set to 20 ppm (orbitrap analyzer) for the analysis of the GlyGly-K modified peptides and the pulldown-MS, while it was set to 0.5 Da (ion trap analyzer) for the shotgun samples. Enzyme specificity was set as C-terminal to arginine and lysine (trypsin), also allowing cleavage at arginine/lysine–proline bonds with a maximum of three missed cleavages for the identification of GlyGly-K modified peptides or set to two missed cleavages for the identification of protein. Carbamidomethylation of cysteine residues was set as a fixed

modification in the analyses of B cells. In all analyses, variable modifications were set to oxidation of methionine (to sulfoxides) and acetylation of protein N-termini. To identify GlyGly-K modified peptides, GlyGly modification of lysine residues was set as an additional variable modification. In the pulldown-MS, phosphorylation of Serine, Threonine or Tyrosine was set as an additional variable modification. Score for modified peptides was set to 40 or 30 for the identification of proteins or GlyGly-K modification sites, respectively. In the pulldown-MS shown in Figure 3E, score for modified peptides was set to 35 for the identification of proteins and GlyGly-K modification sites. In all searches, matching between runs was enabled with an alignment time window of 20 min and a matching time window of 1 min. Only proteins with at least one peptide were retained to compile a list of identified proteins. In the analysis of B cells, 3,273 mouse proteins, and 7,933 GG-modification sites were identified. In the pulldown-MS shown in Figure 3E, 1,389 human proteins and 126 GG-modification sites were identified. In the pulldown-MS shown in Figure 3H, 4,417 human proteins and 554 GlyGly-K modification sites were identified. Proteins were quantified by the MaxLFQ algorithm integrated in the MaxQuant software. A minimum of two ratio counts from at least one unique peptide was required for quantification in all analyses except for the pulldown-MS for which a minimum of two ratio counts from at least one unique or razor peptide was required.

Further data analysis was performed with the Perseus software (version 1.5.5.3, 1.6.1.1 or 1.6.2.3). For the analysis of the shotgun data, the proteinGroups table from MaxQuant was loaded in Perseus, reverse database hits were removed as well as proteins only identified by sites. The LFQ intensities were log2 transformed and replicate samples were grouped as above. Proteins with less than three valid values in at least one group were removed and missing values were imputed from a normal distribution around the detection limit. To reveal the proteins that were significantly regulated, sample groups were defined based on genotype (WT-USP8 vs. $USP8_{C748A}$) and a t-test (FDR = 0.05 and S0 = 1) was performed to compare the intensity of the proteins between the two genotypes. Quantified proteins (n = 2,251) and the results of the t-

tests are shown in Figure 3B. For the analysis of GlyGly-K modification sites, the GlyGly (K) Sites table from MaxQuant was loaded and reverse database hits were removed. The site table was expanded, intensities were log2 transformed and normalized for each sample by subtracting the median intensity. Replicate samples were grouped, sites with less than three valid values in at least one group were removed, and missing values were imputed from a normal distribution around the detection limit. To reveal sites that were significantly regulated, sample groups were defined based on genotype (WT-USP8 vs. $USP8_{C748A}$) and a t-test (FDR = 0.05 and S0 = 1) was performed to compare the intensities of the sites between the two genotypes. Quantified GlyGly-K modified sites (n = 5,824) and the results of the t-tests are shown in Figure 3B. In all analyses, the same workflow as described above was applied for the analysis of GlyGly-K modification sites or for the analysis of proteins. For the pulldown-MS analysis shown in figure 3E, 845 proteins 49 GG-modification sites were evaluated. For the pulldown-MS analysis shown in Figure 3H, only identified GlyGly-K modification sites that are derived from NEDD8 or Ubiquitin proteins were displayed in the heatmap.

Statistical analysis. We ensured that our sample sizes matched those generally used in the field. T-testing, or 1-way or 2-way ANOVA and Tukey's post hoc tests were applied and differences with a P value of <0.05 were considered significant. Excel and GraphPad Prism 10 were used for statistical testing if not mentioned otherwise.

The transcriptomic data and clinical data of the Multiple Myeloma Research Foundation (MMRF) CoMMpass study was retrieved from the Genomic Data Commons (GDC) data portal. Raw Counts were normalized for counts per million (CPM). The remaining samples were sorted according to the expression of USP8 and were stratified in two groups (<median and > median). Kaplan-Meier overall survival plots on these three groups of samples were generated using the function TCGAanalyze_survival from the R package TCGAbiolinks v2.30[18].

The boxplot with patients stratified by International Staging System (ISS) was generated using ggplot2 v3.4.4 and ggpubr v0.6.

1 Niendorf S, Oksche A, Kisser A, Löhler J, Prinz M, Schorle H *et al.* Essential Role of Ubiquitin-Specific Protease 8 for Receptor Tyrosine Kinase Stability and Endocytic Trafficking In Vivo. *Mol Cell Biol* 2007; **27**: 5029.

2 Rickert RC, Roes J, Rajewsky K. B lymphocyte-specific, Cre-mediated mutagenesis in mice. *Nucleic Acids Res* 1997; **25**: 1317–1318.

3 Hobeika E, Thiemann S, Storch B, Jumaa H, Nielsen PJ, Pelanda R *et al.* Testing gene function early in the B cell lineage in mb1-cre mice. *Proc Natl Acad Sci U S A* 2006; **103**: 13789–13794.

4 Hameyer D, Loonstra A, Eshkind L, Schmitt S, Antunes C, Groen A *et al.* Toxicity of ligand-dependent Cre recombinases and generation of a conditional Cre deleter mouse allowing mosaic recombination in peripheral tissues. *Physiological Genomics* 2007; **31**: 32–41.

5 Ventura A, Kirsch DG, McLaughlin ME, Tuveson DA, Grimm J, Lintault L *et al.* Restoration of p53 function leads to tumour regression in vivo. *Nature* 2007; **445**: 661–665.

6 Bertossi A, Aichinger M, Sansonetti P, Lech M, Neff F, Pal M *et al.* Loss of Roquin induces early death and immune deregulation but not autoimmunity. *J Exp Med* 2011; **208**: 1749–1756.

7 Vogel KU, Edelmann SL, Jeltsch KM, Bertossi A, Heger K, Heinz GA *et al.* Roquin Paralogs 1 and 2 Redundantly Repress the Icos and Ox40 Costimulator mRNAs and Control Follicular Helper T Cell Differentiation. *Immunity* 2013; **38**: 655–668.

8 Shultz LD, Lyons BL, Burzenski LM, Gott B, Chen X, Chaleff S *et al.* Human lymphoid and myeloid cell development in NOD/LtSz-scid IL2R gamma null mice engrafted with mobilized human hemopoietic stem cells. *J Immunol* 2005; **174**: 6477–6489.

9 Young MD, Behjati S. SoupX removes ambient RNA contamination from droplet-based single-cell RNA sequencing data. *GigaScience* 2020; **9**: giaa151.

10 Linderman GC, Zhao J, Roulis M, Bielecki P, Flavell RA, Nadler B *et al.* Zero-preserving imputation of single-cell RNA-seq data. *Nature Communications* 2022; **13**: 192.

11 Nguyen HTT, Guevarra RB, Magez S, Radwanska M. Single-cell transcriptome profiling and the use of AID deficient mice reveal that B cell activation combined with antibody class switch recombination and somatic hypermutation do not benefit the control of experimental trypanosomosis. *PLoS Pathog* 2021; **17**: e1010026.

12 Berger I, Fitzgerald DJ, Richmond TJ. Baculovirus expression system for heterologous multiprotein complexes. *Nat Biotechnol* 2004; **22**: 1583–1587.

13 Basters A, Geurink PP, El Oualid F, Ketscher L, Casutt MS, Krause E *et al.* Molecular characterization of ubiquitin-specific protease 18 reveals substrate specificity for interferon-stimulated gene 15. *FEBS J* 2014; **281**: 1918–1928.

14 Fitzgerald DJ, Berger P, Schaffitzel C, Yamada K, Richmond TJ, Berger I. Protein complex expression by using multigene baculoviral vectors. *Nat Methods* 2006; **3**: 1021–1032.

15 Dufner A, Kisser A, Niendorf S, Basters A, Reissig S, Schönle A *et al.* The ubiquitin-specific protease USP8 is critical for the development and homeostasis of T cells. *Nature Immunology* 2015; **16**: 950.

16 Blank T, Detje CN, Spieß A, Hagemeyer N, Brendecke SM, Wolfart J *et al.* Brain Endothelial- and Epithelial-Specific Interferon Receptor Chain 1 Drives Virus-Induced Sickness Behavior and Cognitive Impairment. *Immunity* 2016; **44**: 901–912.

17 El Oualid F, Merkx R, Ekkebus R, Hameed DS, Smit JJ, de Jong A *et al*. Chemical synthesis of ubiquitin, ubiquitin-based probes, and diubiquitin. *Angew Chem Int Ed Engl* 2010; **49**: 10149–10153.
18 Colaprico A, Silva TC, Olsen C, Garofano L, Cava C, Garolini D *et al*. TCGAbiolinks: an R/Bioconductor package for integrative analysis of TCGA data. *Nucleic Acids Res* 2016; **44**: e71.
