# Supplementary material for: USP8 controls proteostasis pathways in B cells and multiple myeloma
Source: Leukemia. 2026 Aug 11;40(11):2441–54. doi: 10.1038/s41375-026-03086-y (PMC13713586; doi:10.1038/s41375-026-03086-y)

Fig. 2G (left panels)

Analysis of B cells from USP8$^{f/f}$ and USP8$^{f/f}$CD19-Cre mice
Western blots: 3 independent experiments

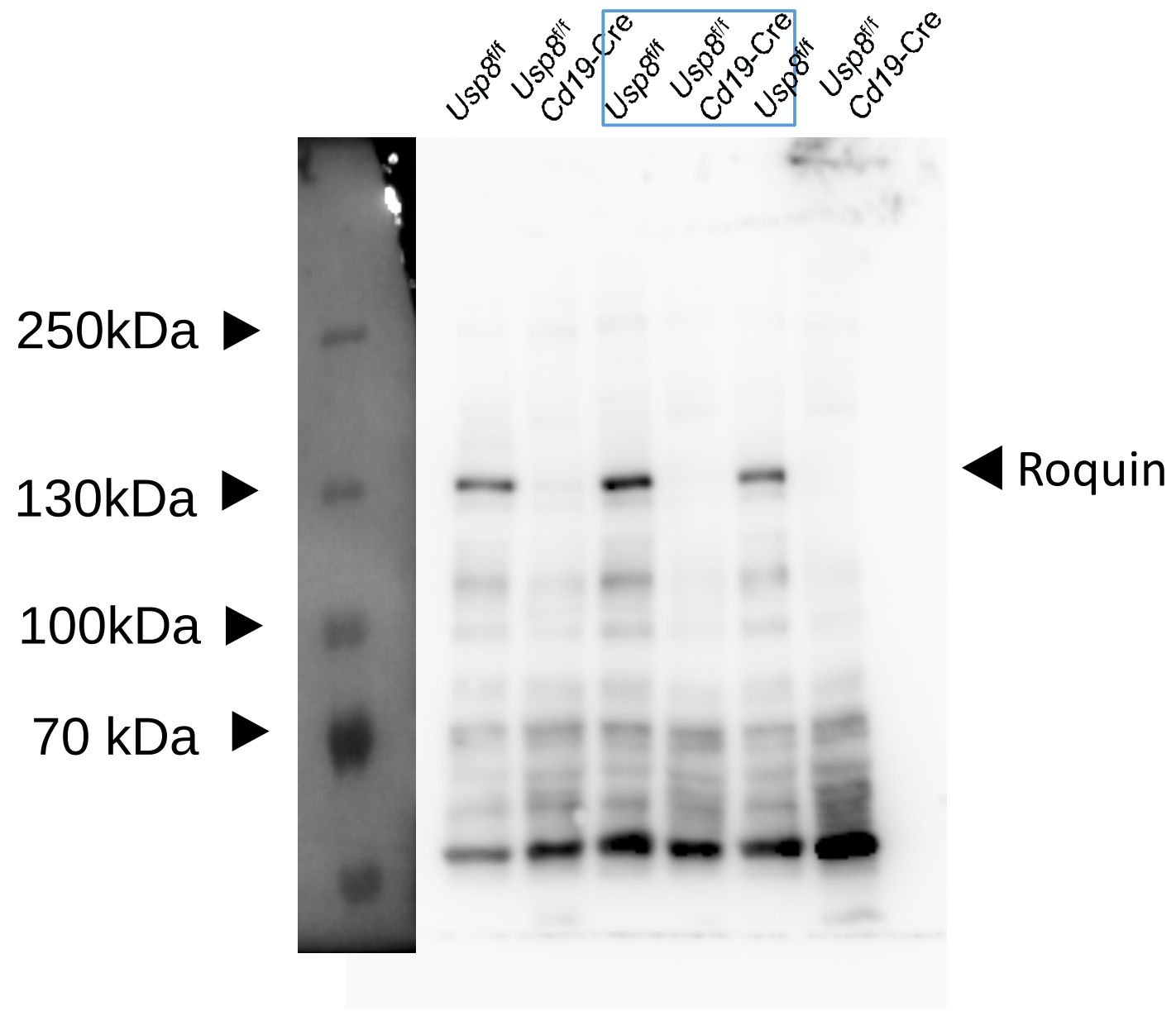

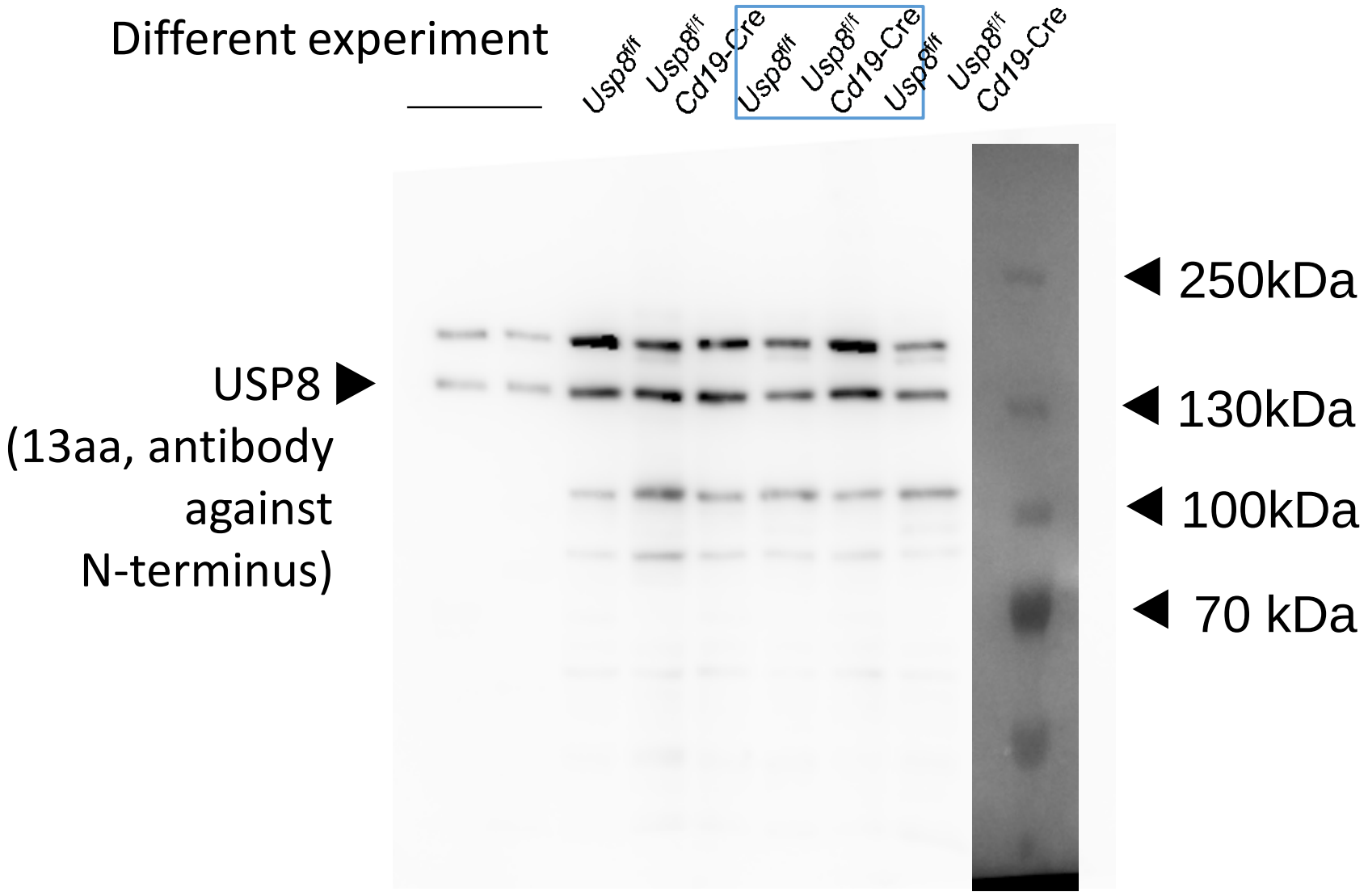

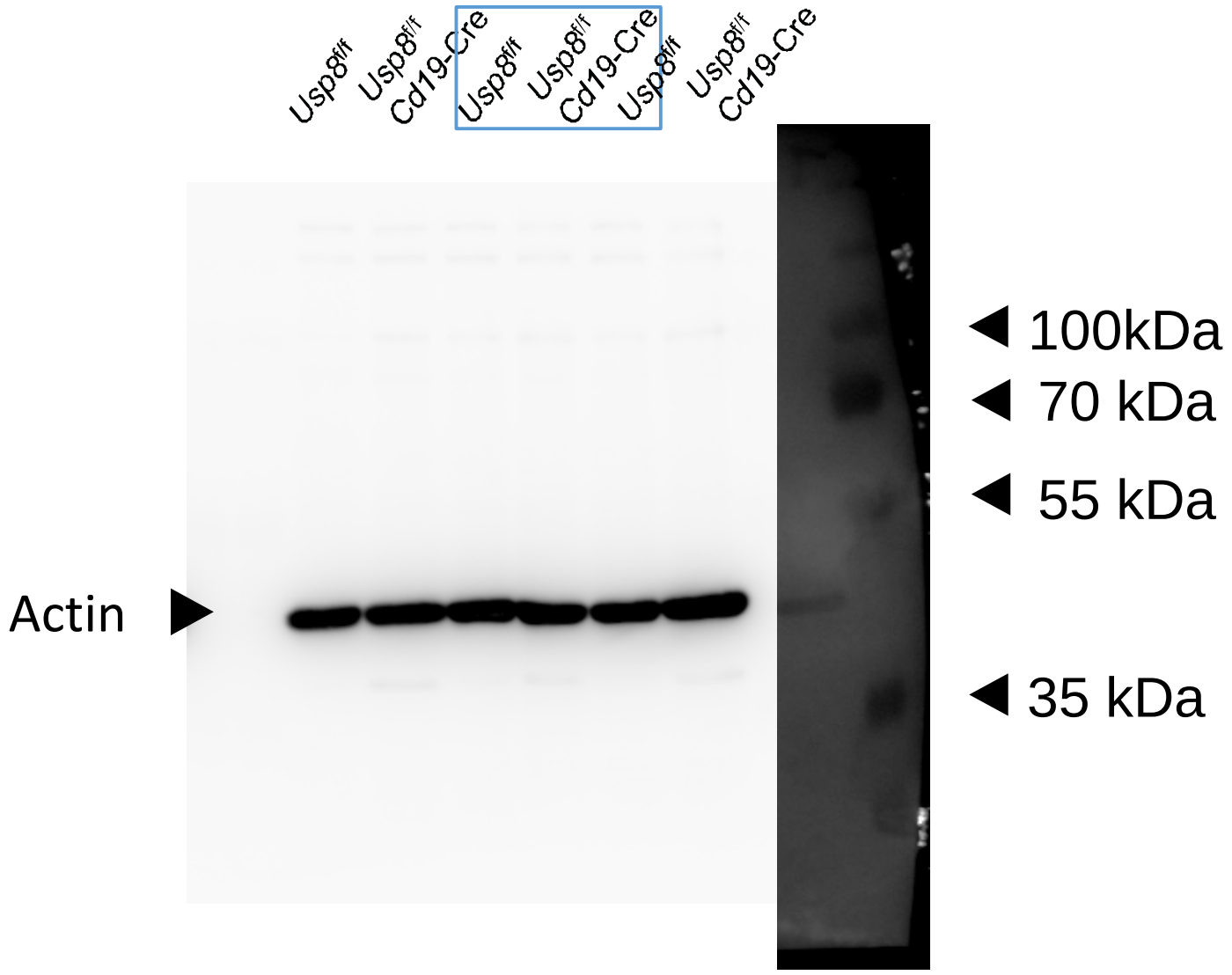

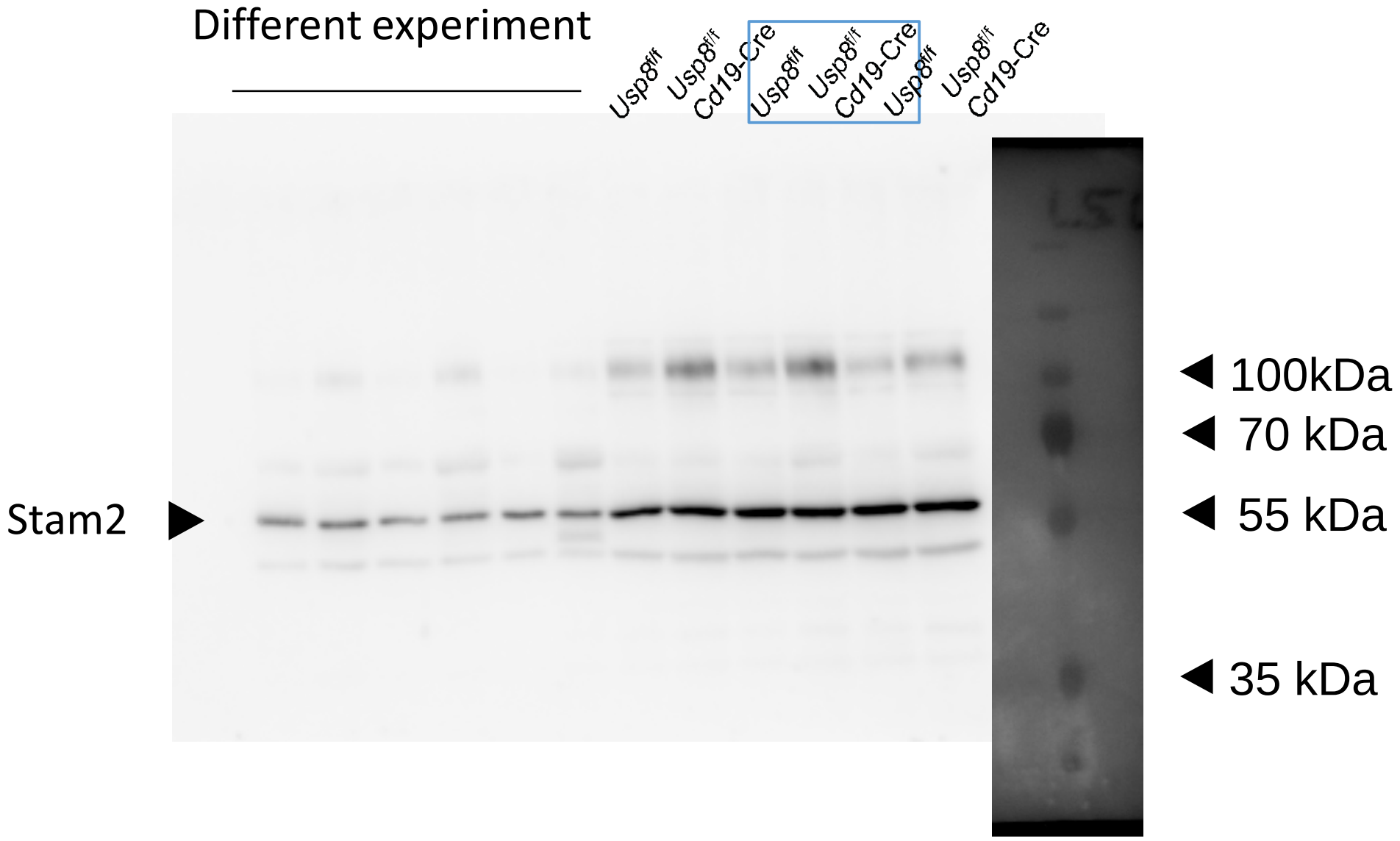

Fig. 2G (right panels) Analysis of B and T cells from USP8$^{f/f}$ and USP8$^{f/f}$CD19-Cre mice

Western blots: 2 independent experiments (8 wks old male mice, littermates: 1+2, 3+4)

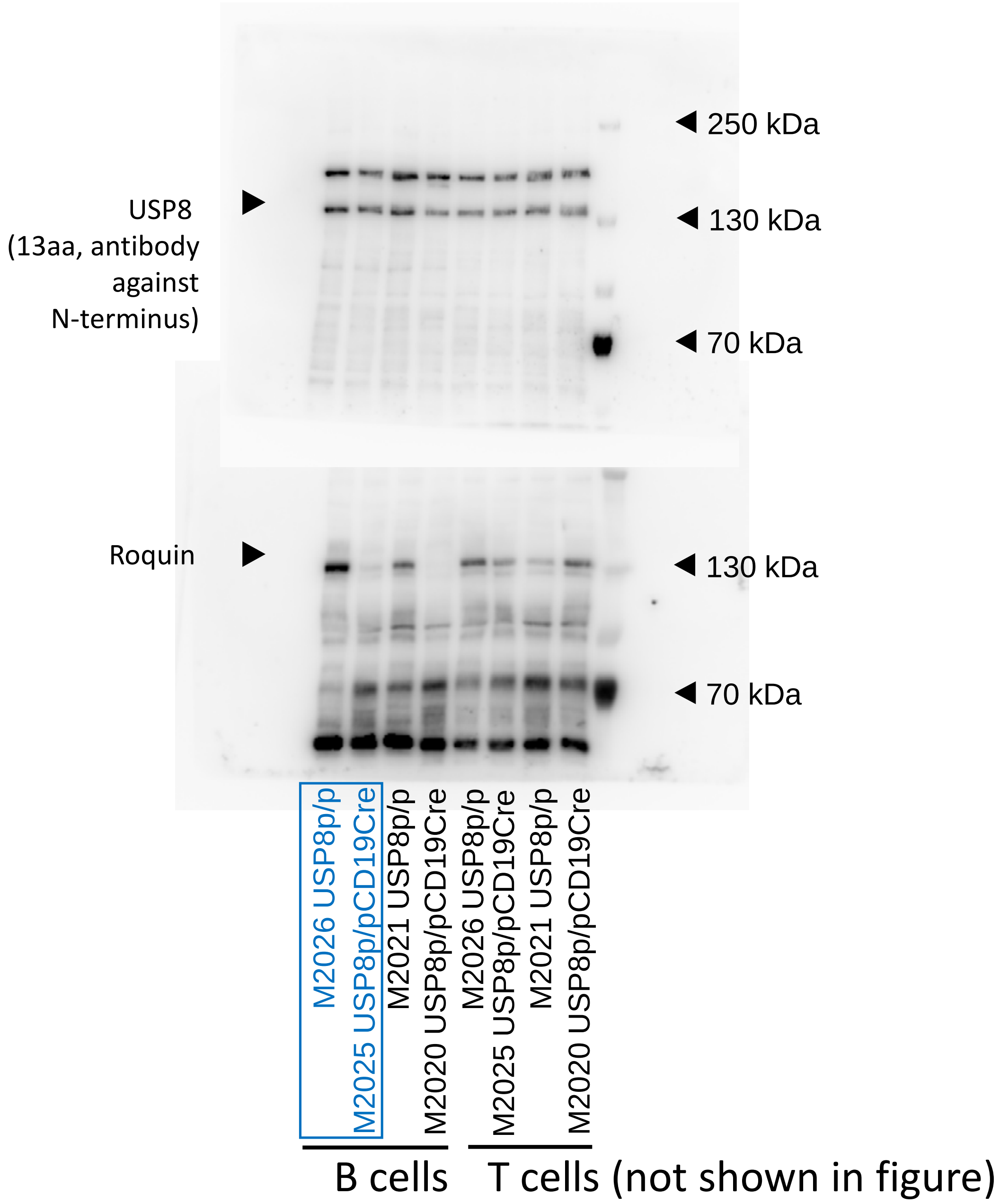

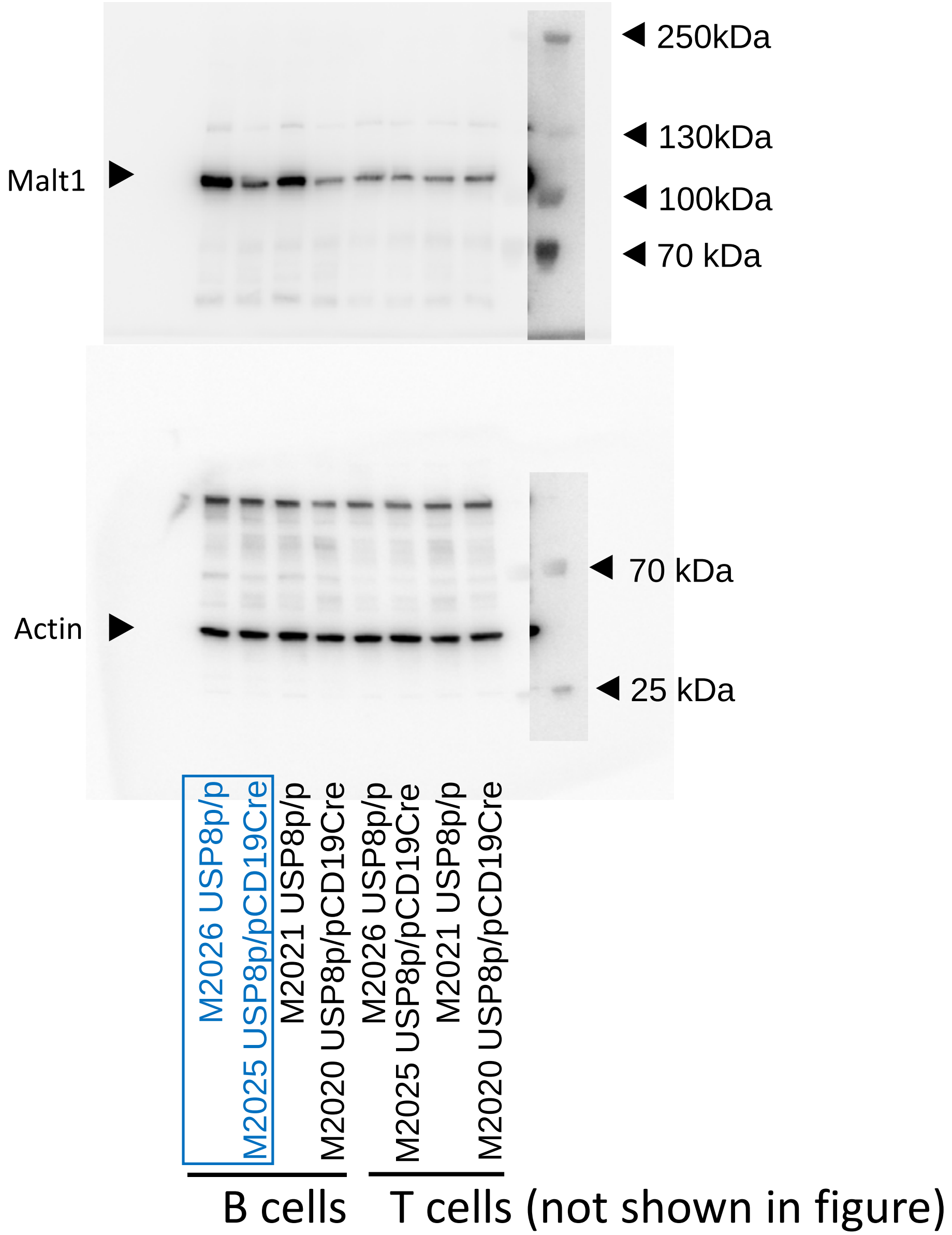

Fig. 2H

Signaling (phospho-JNK) in Usp8$^{f/f}$ and Usp8$^{f/f}$Cd19Cre B cells

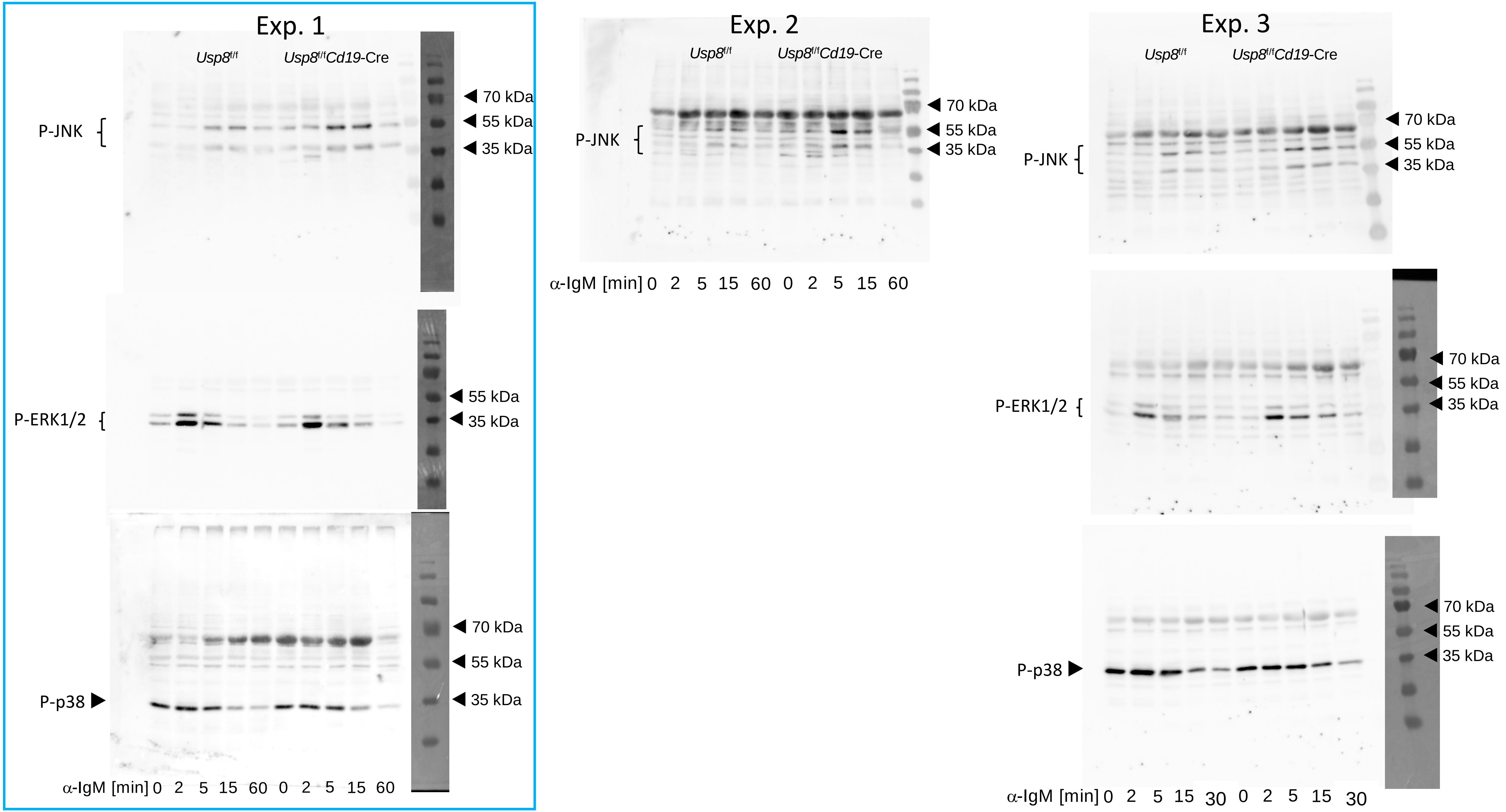

Fig. 2H

Signaling (phospho-JNK) in Usp8$^{f/f}$ and Usp8$^{f/f}$Cd19Cre B cells

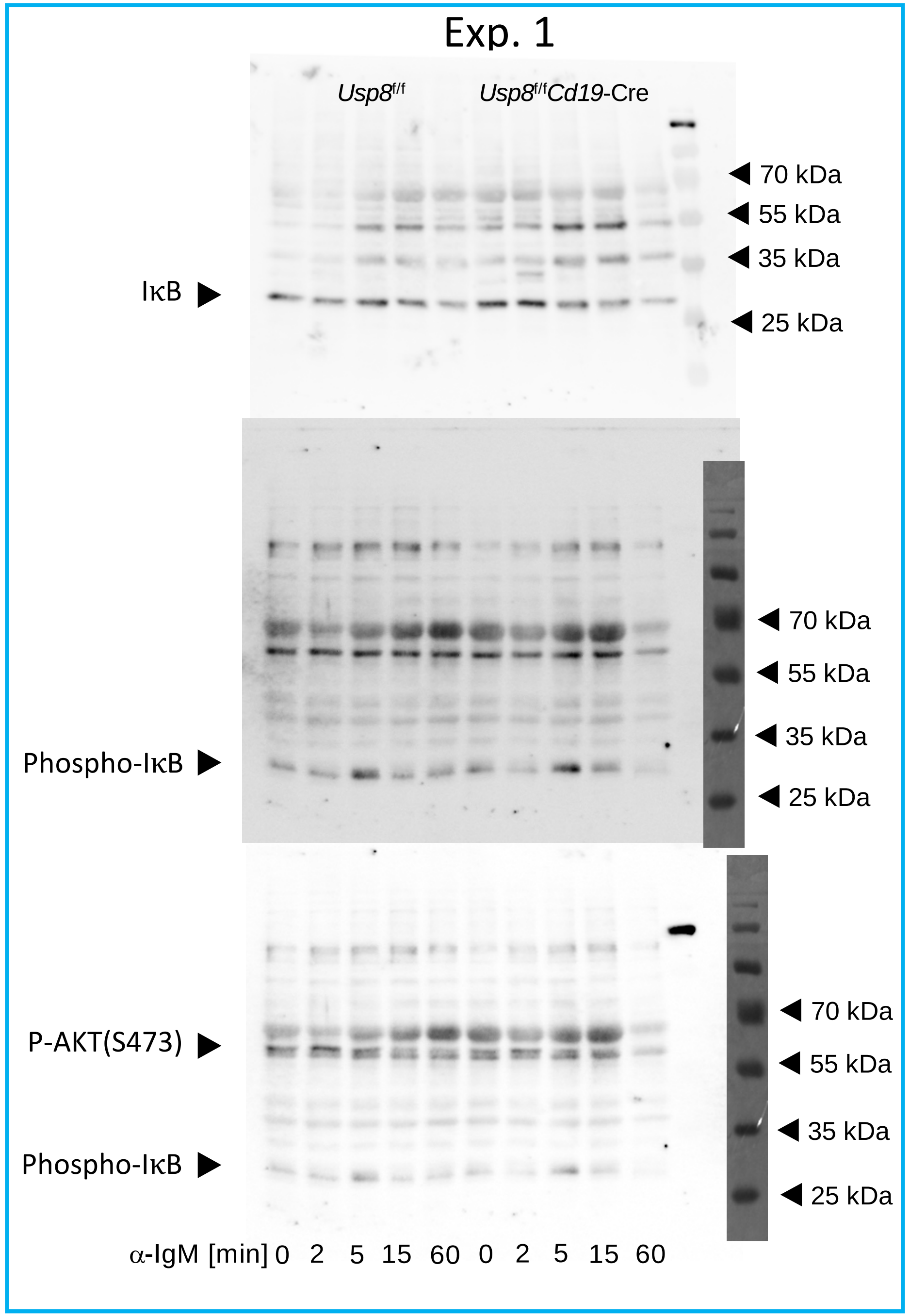

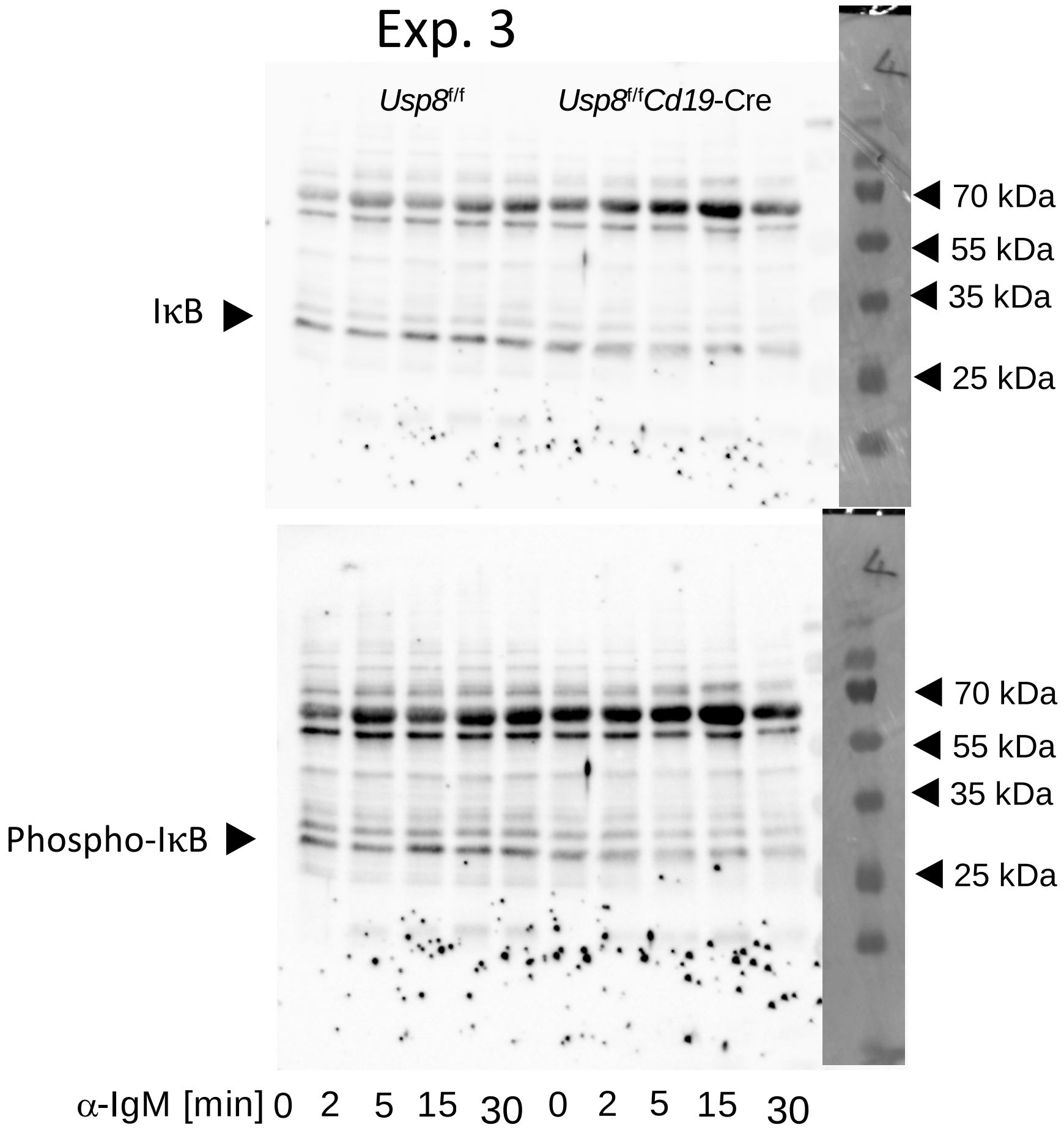

Fig. 2H

Signaling (phospho-JNK) in Usp8$^{f/f}$ and Usp8$^{f/f}$Cd19Cre B cells

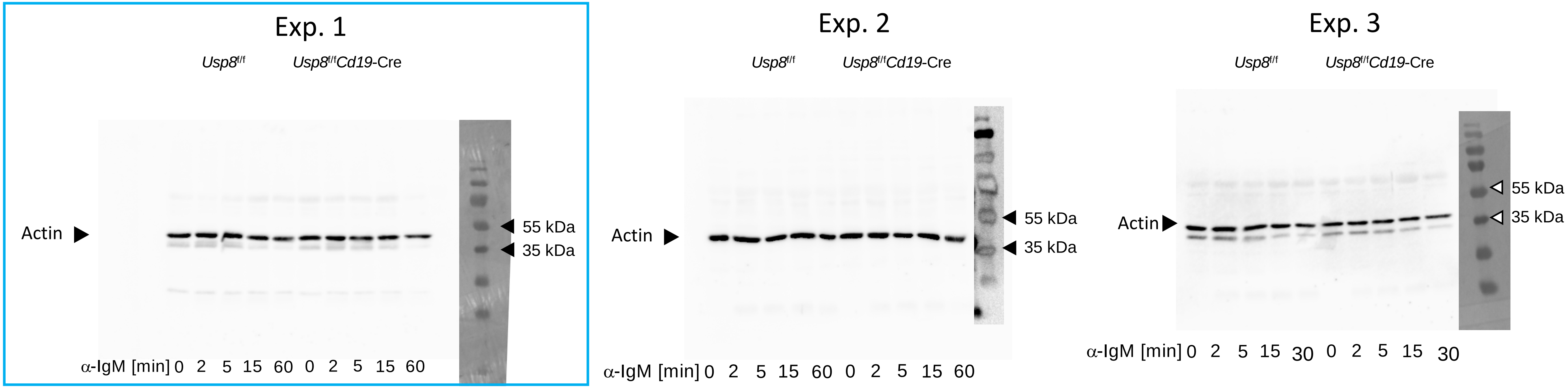

Fig. 2I JNK activation in Roquin1/2 depleted B cells: 3 independent experiments

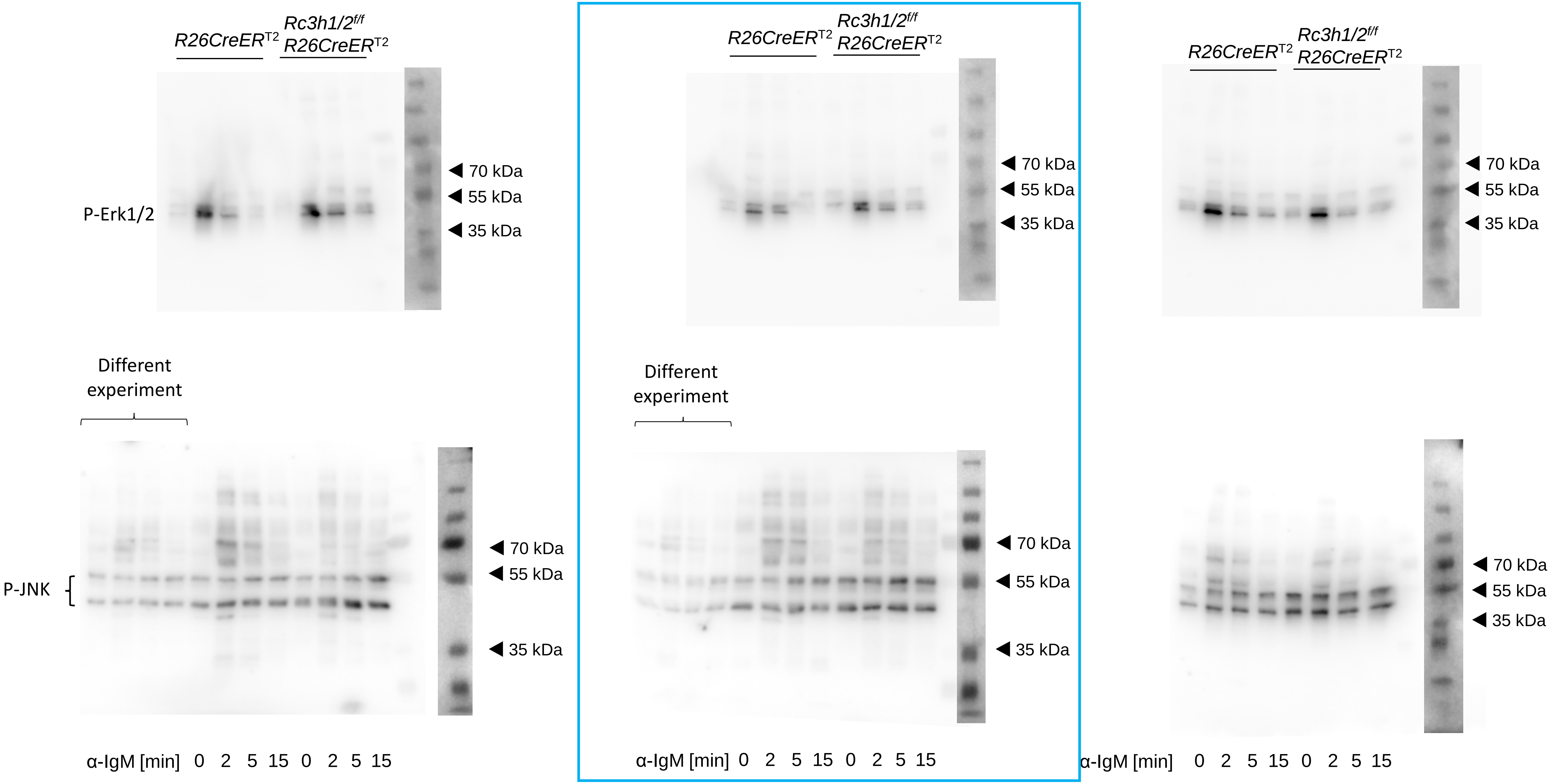

Fig. 2I JNK activation in Roquin1/2 depleted B cells: 3 independent experiments

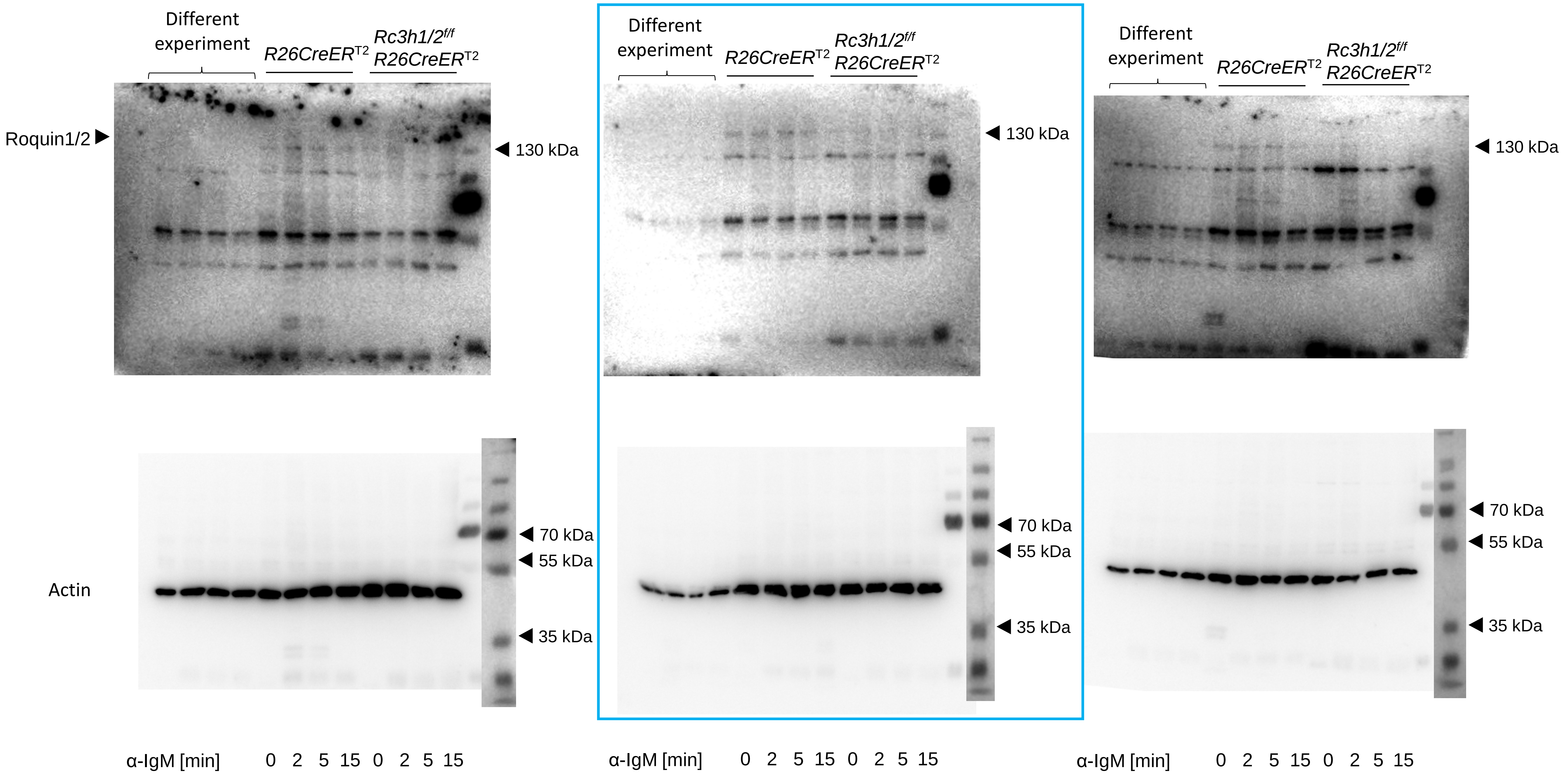

Fig. 3A

USP8 expression in K46Lµm B cells

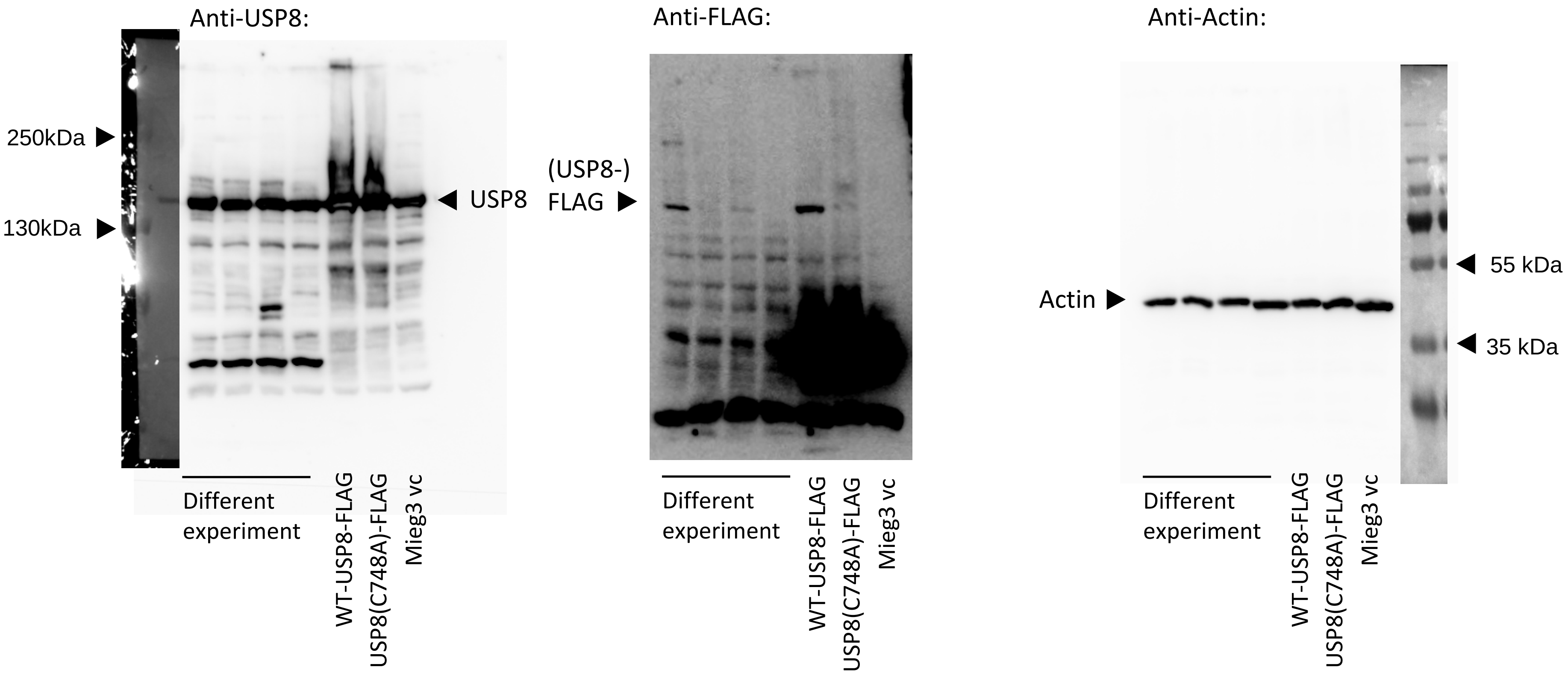

Fig. 3C/D

Anti-HA-Nedd8/FLAG-USP8 IP in 293T cells

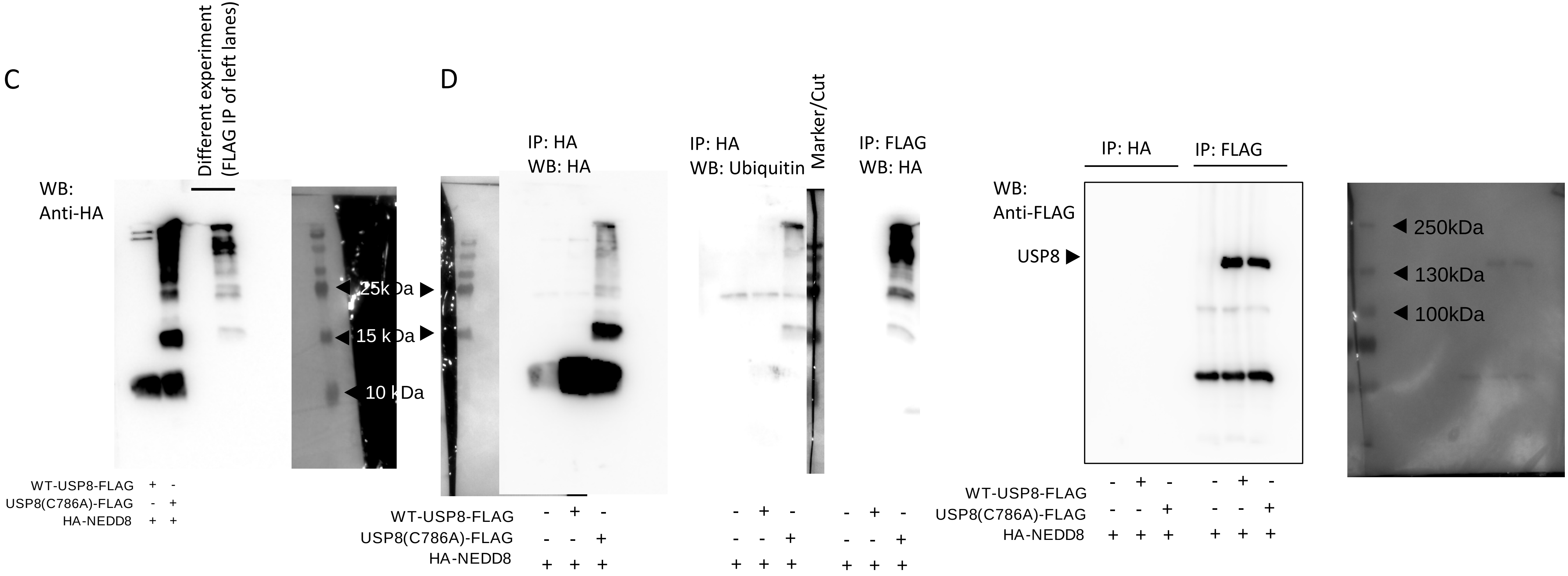

Fig. 3F

USP8 regulates non-canonical Neddylation pathway

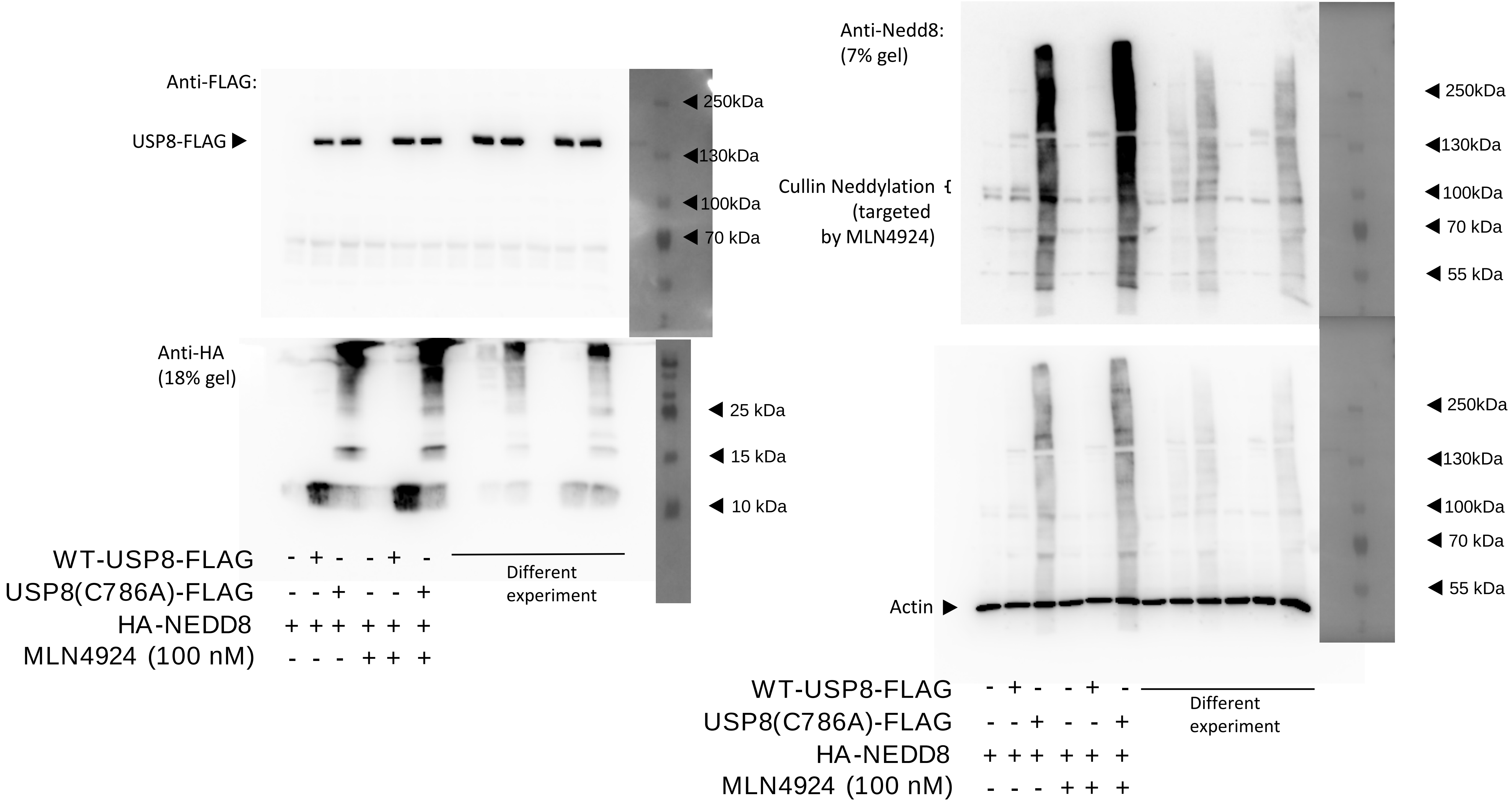

Fig. 3F

USP8 regulates non-canonical Neddylation pathway

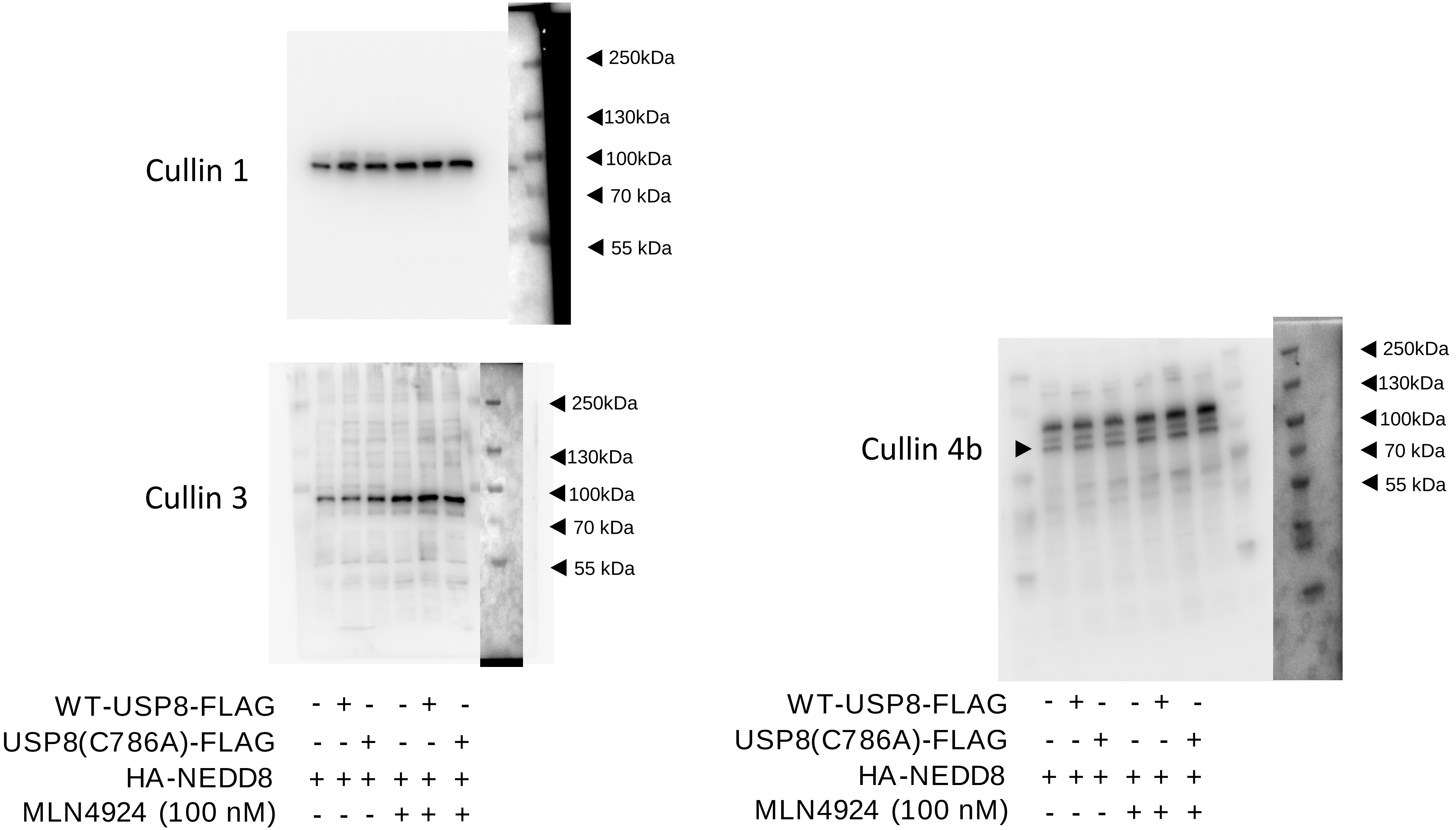

Fig. 3G USP8 does not have deNeddylation capacity: 2 independent experiments

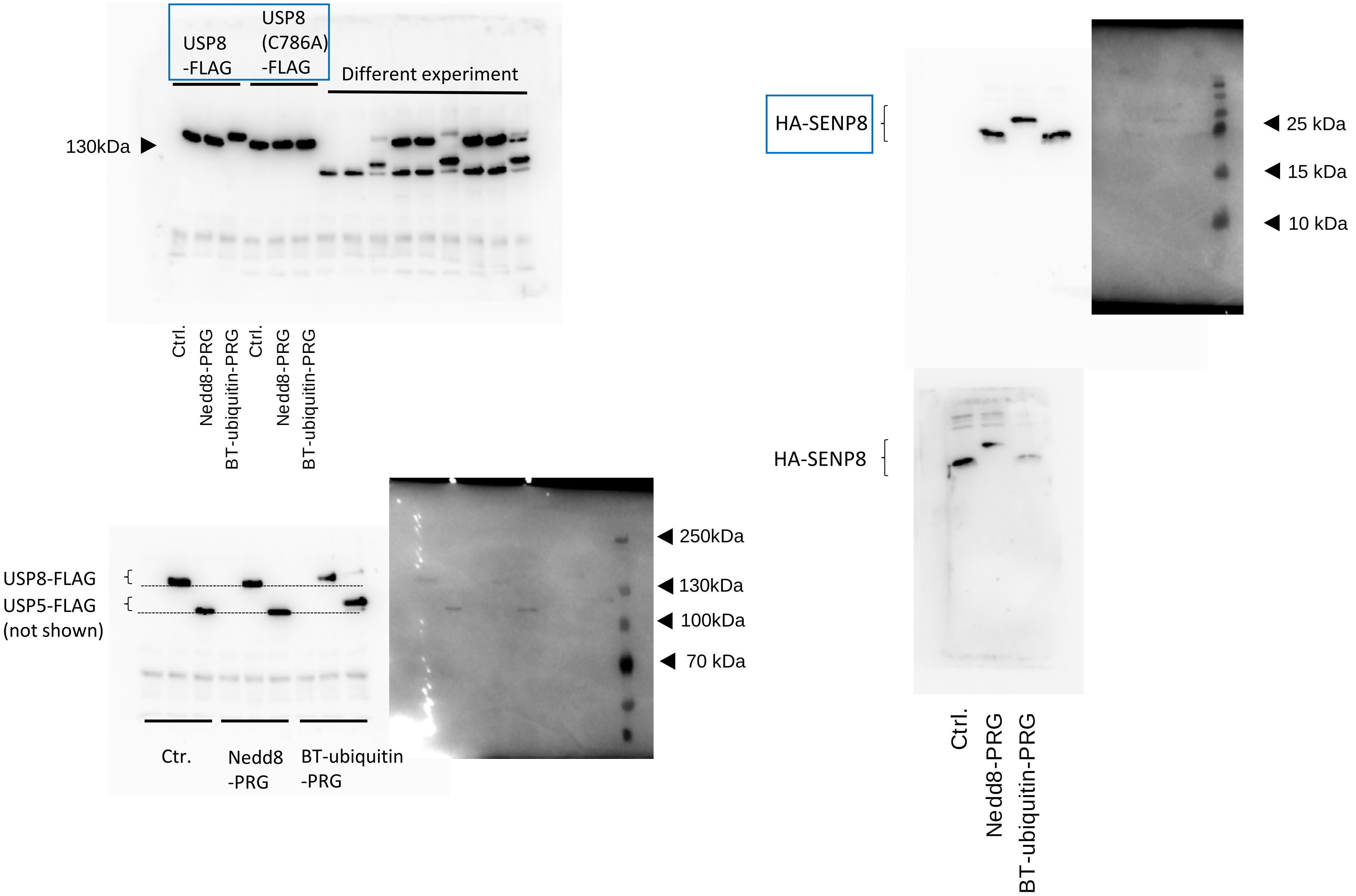

Fig. 3H NEDD8-LTGG characterization

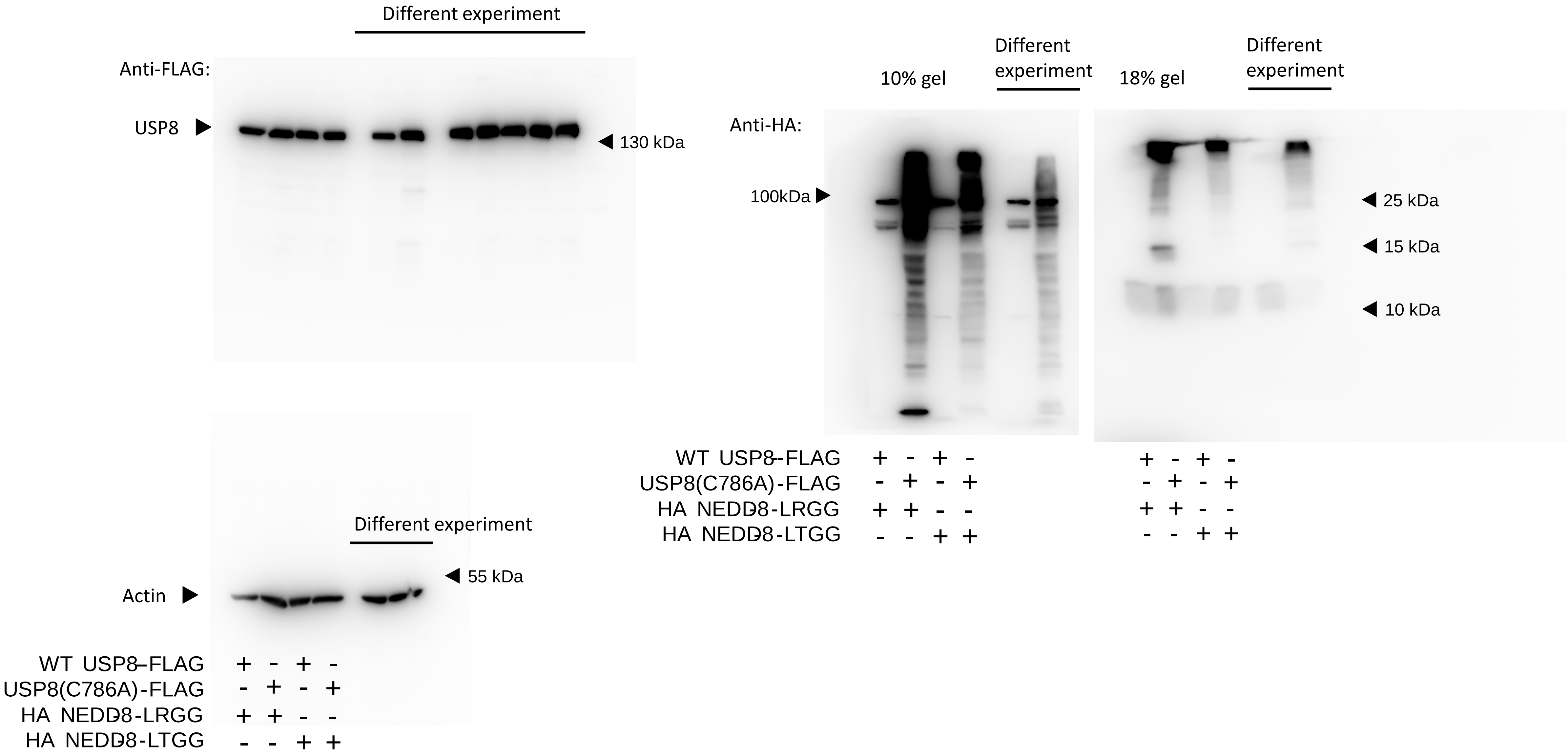

Fig. 3I USP8 targets mixed chains *in vitro*

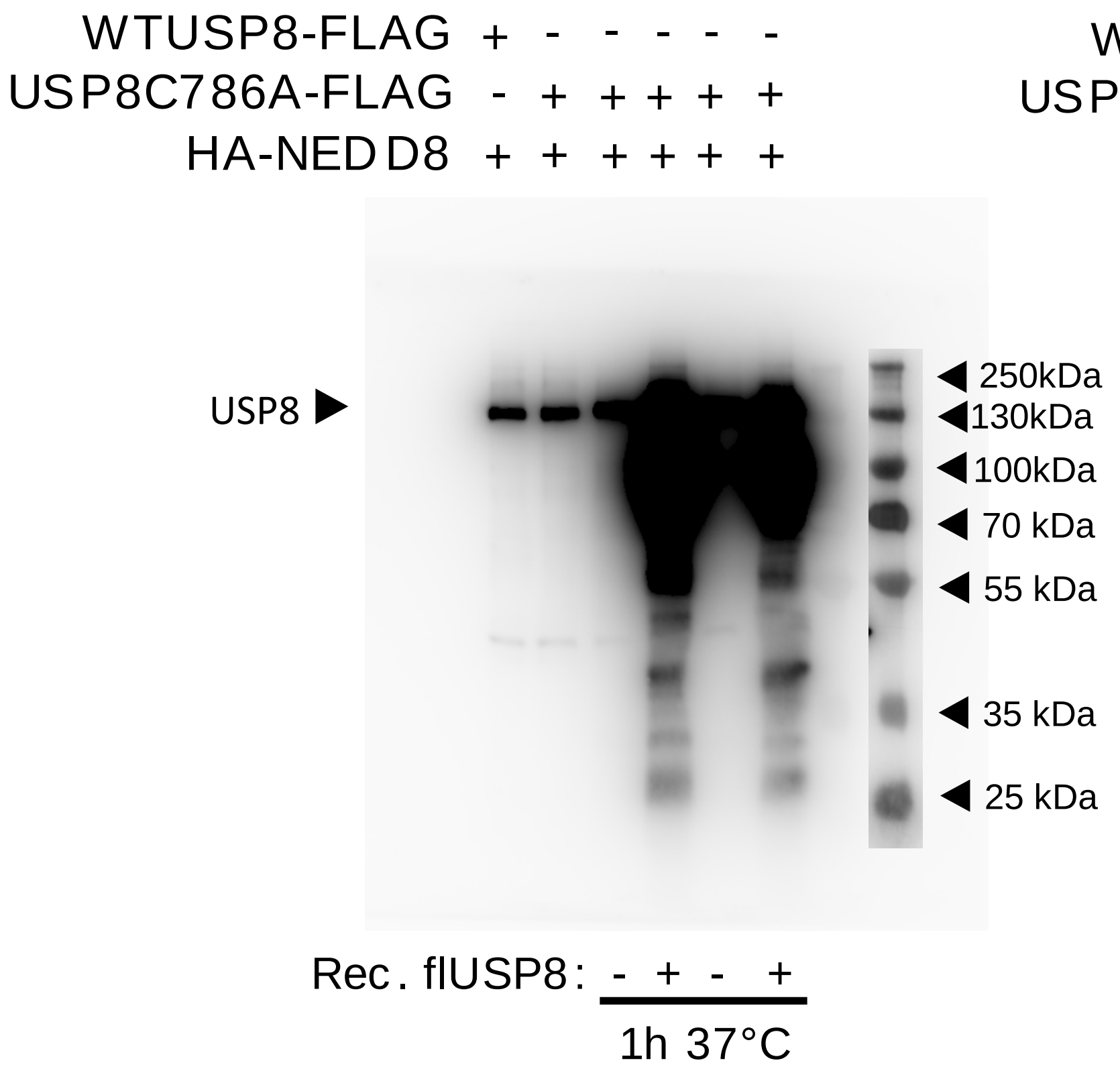

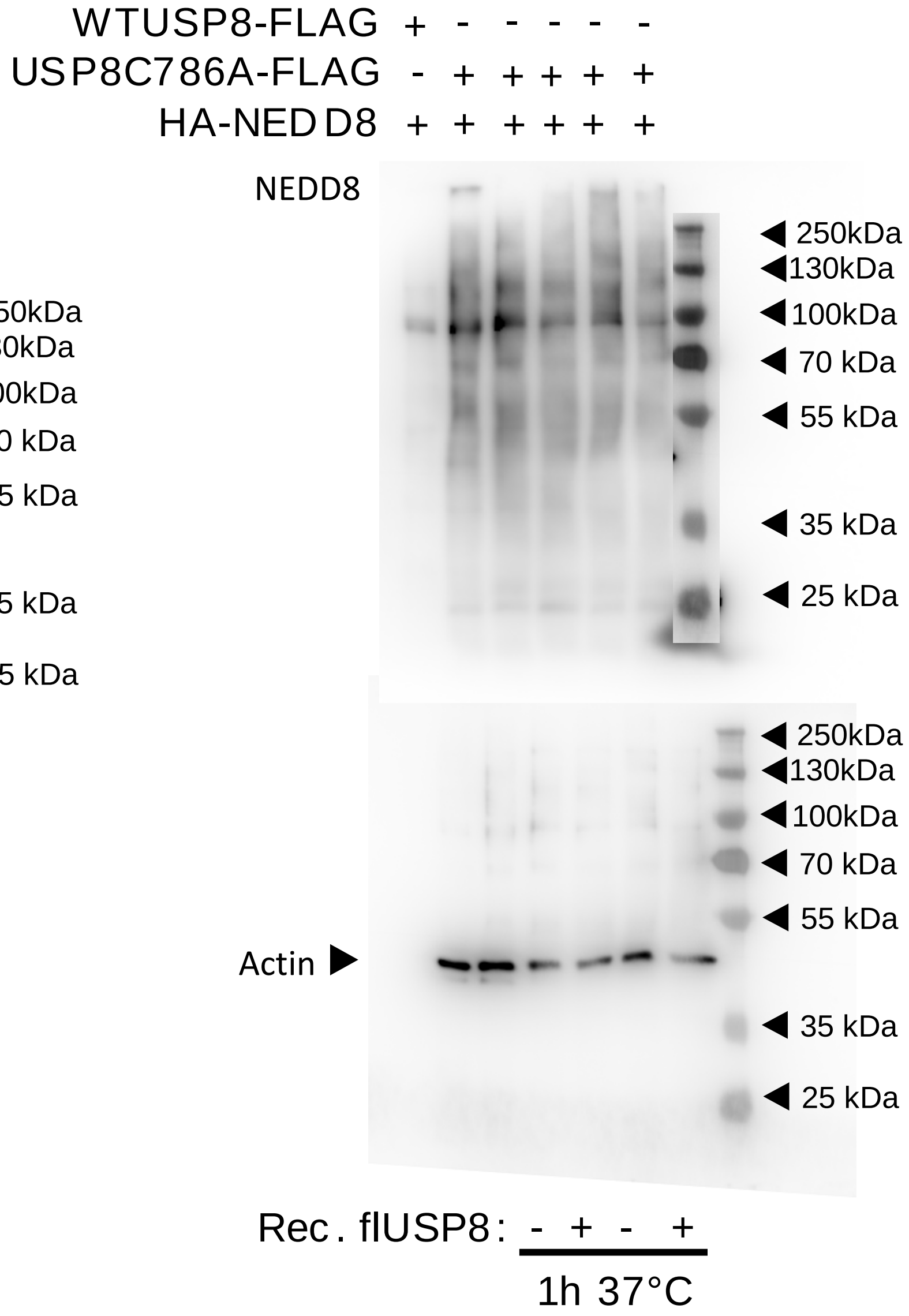

Fig. 3J

USP8 targets mixed chains *in vitro*: 3 independent experiments

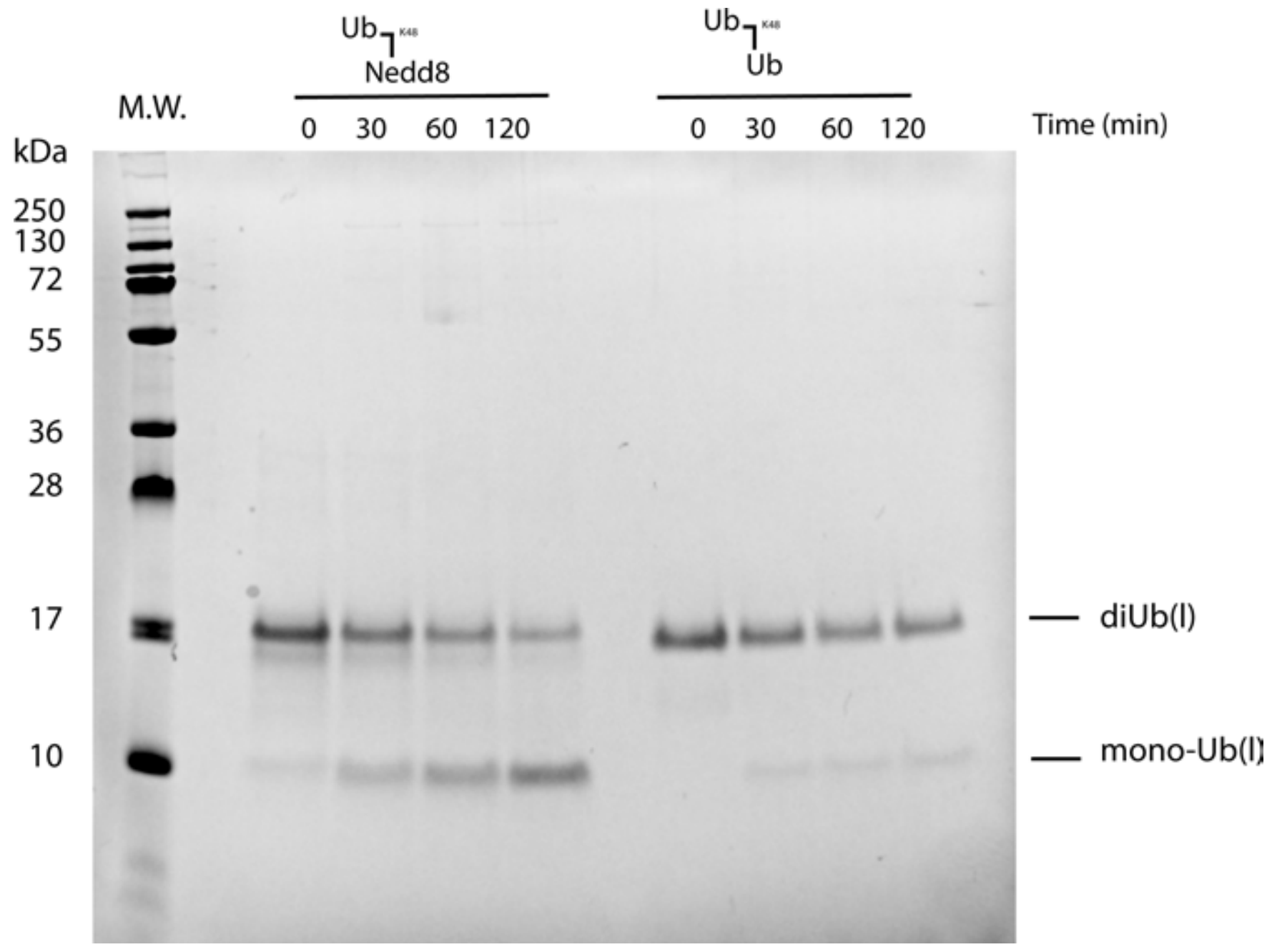

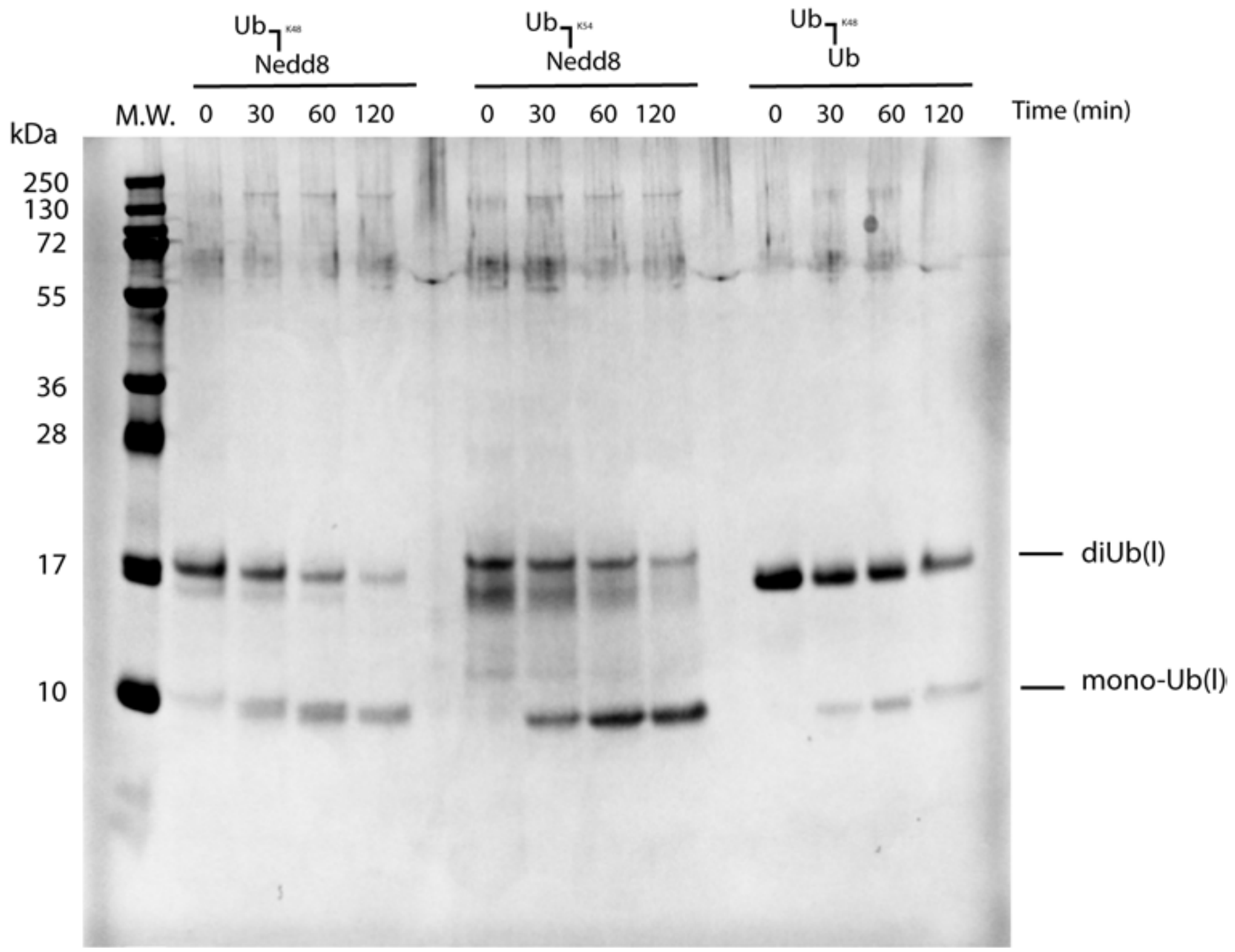

Fig. 4A Western Blots: Induced USP8 deletion in B cells: 2 independent experiments 08.02.2019, 15.04.2019

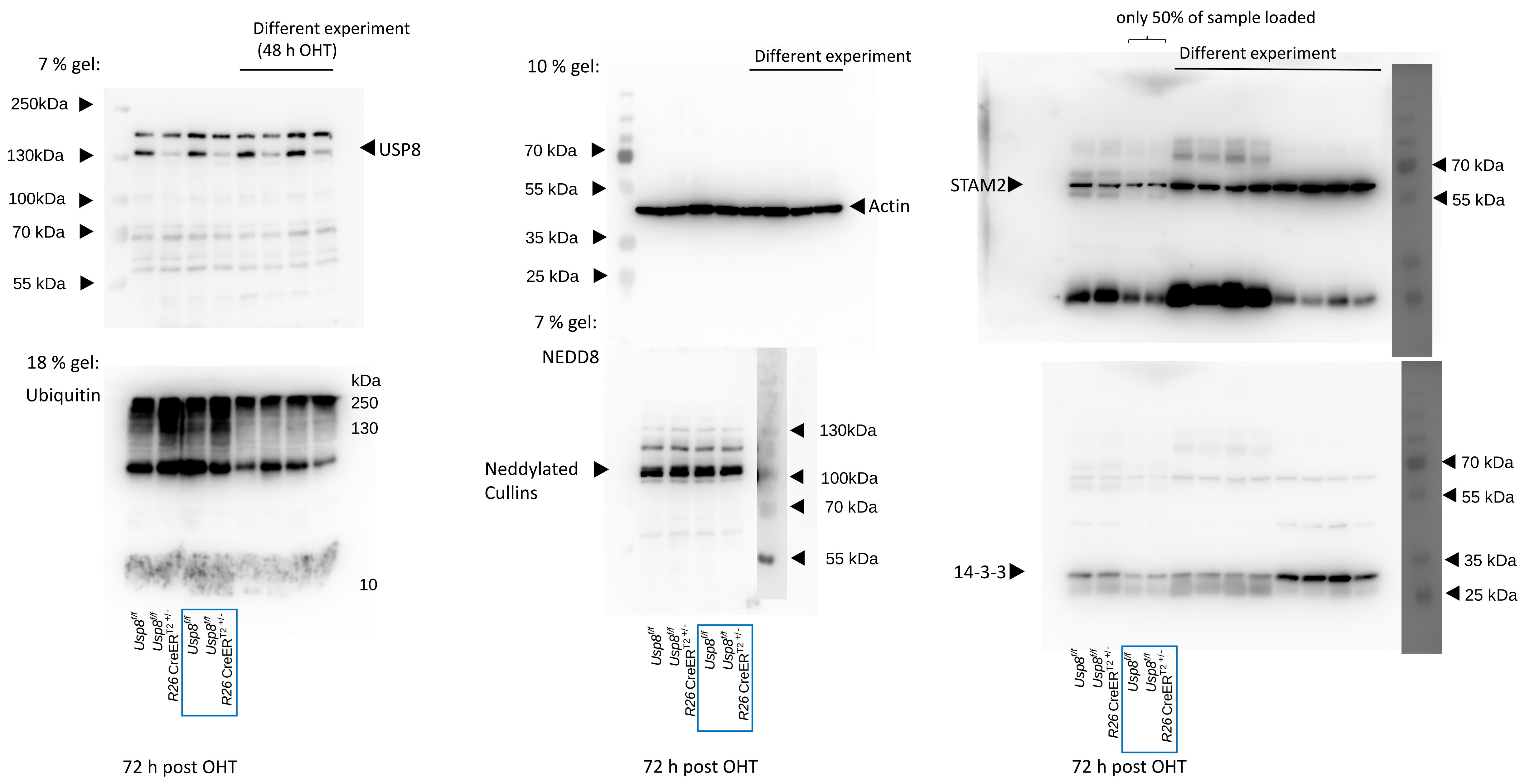

Fig. 4B

Densitometry of western blot (see source data file):
Caspase 8 (p10), Cleaved Caspase 3, Ubiquitin, JNK densitometry normalized to Actin control, normalized to USP8$^{f/f}$
3 independent experiments (6 mice, littermates: 1+2; 3+4+5+6)

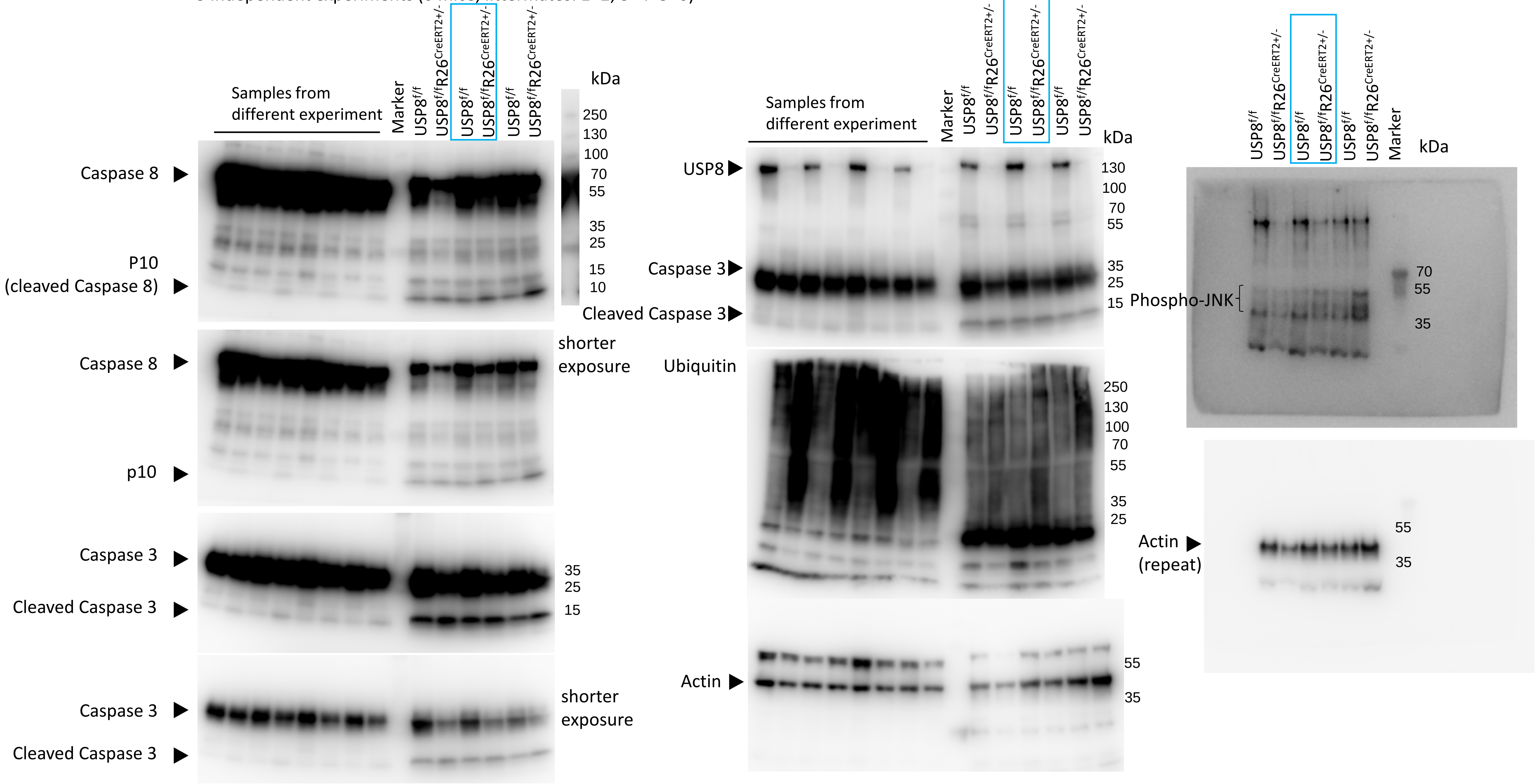

Fig. 4C

Western blots: 2 independent experiments (4 mice, littermates: 1+2; 3+4)

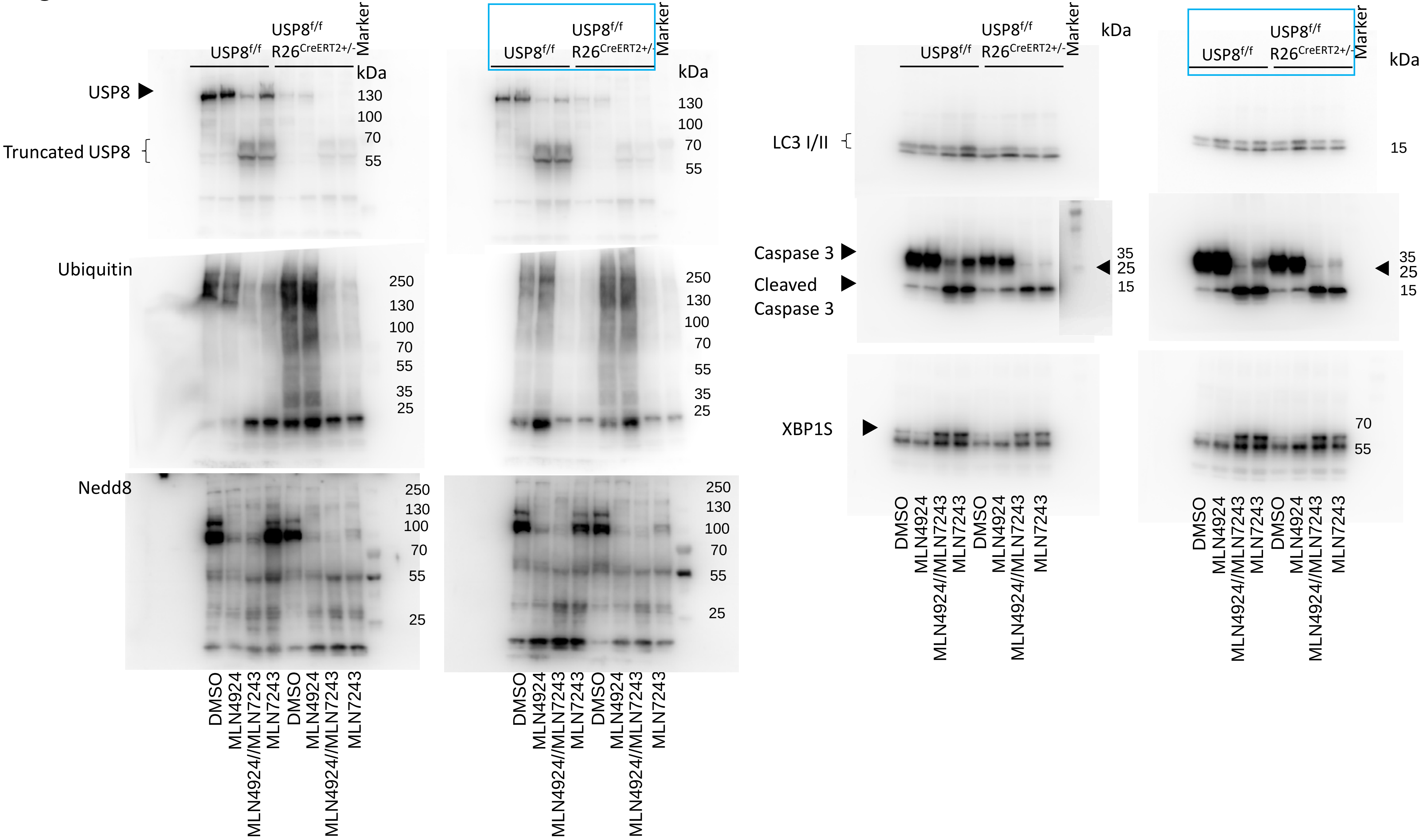

Fig. 4C

Western blots: 2 independent experiments (4 mice, littermates: 1+2; 3+4)

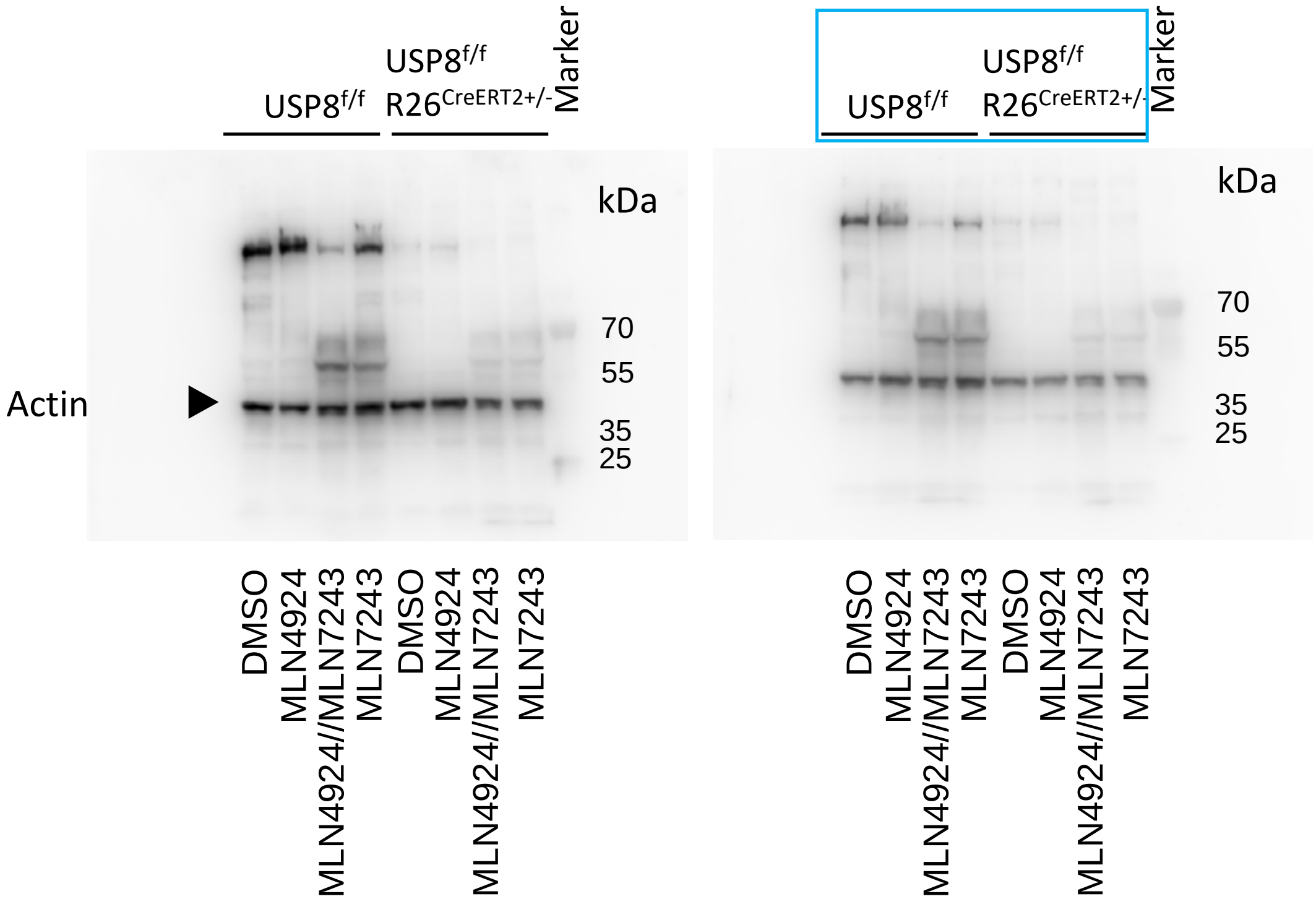

Fig. 4E KMS-18, 6h Western blots: 2 independent experiments

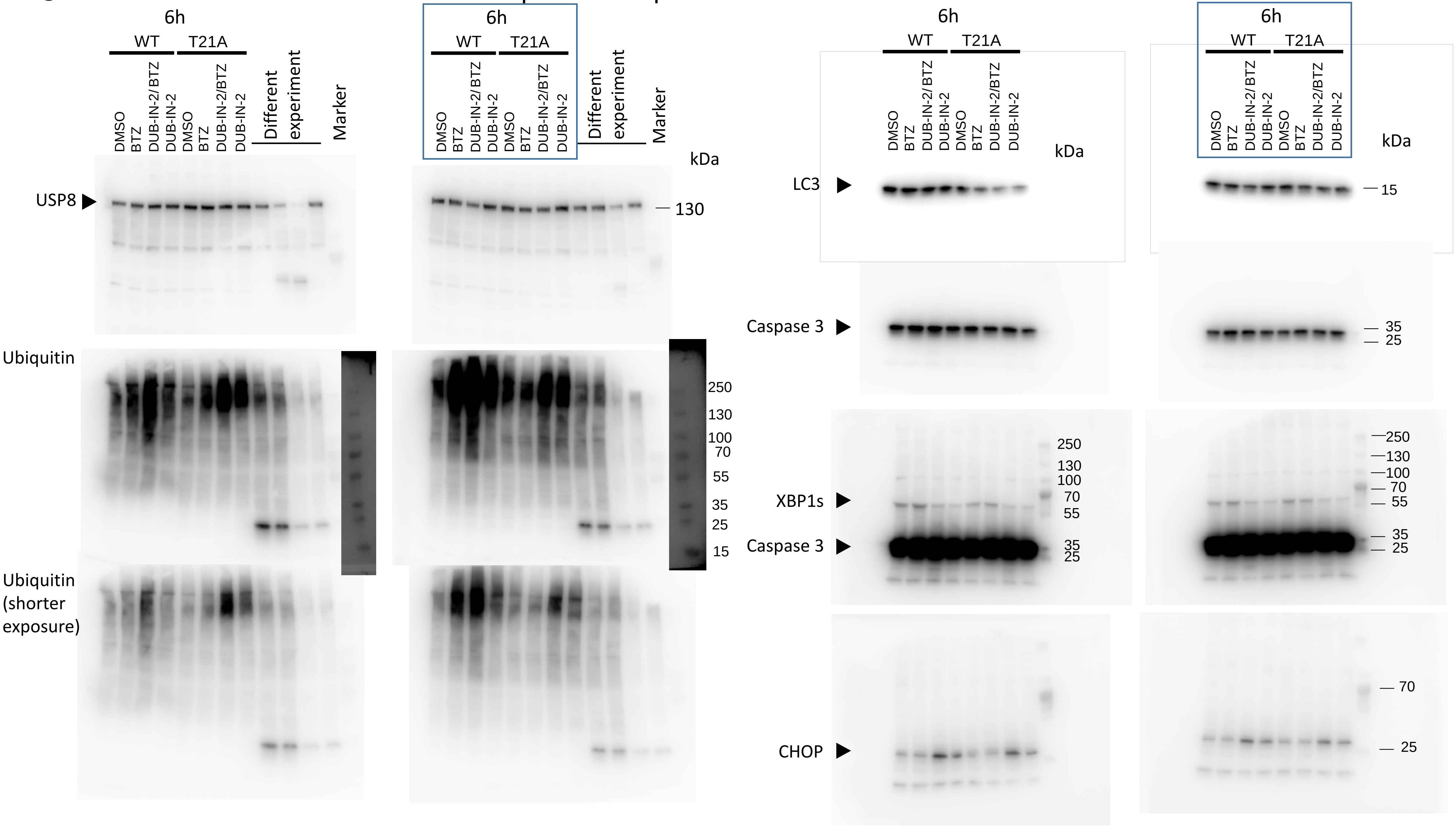

Fig. 4E KMS-18, 6h   Western blots: 2 independent experiments

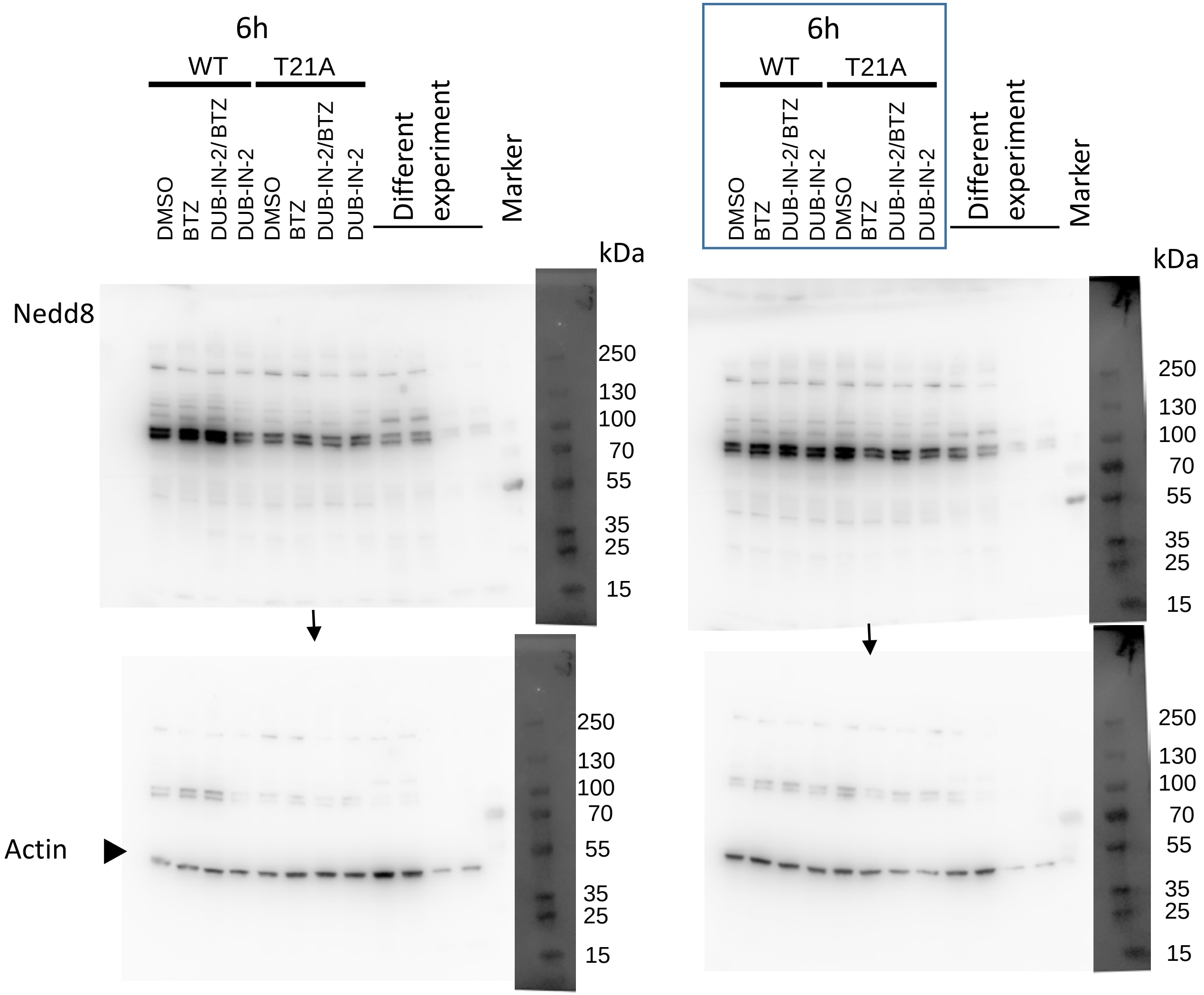

Fig. 4E KMS18, 24h   Western blots: 2 independent experiments shown for ubiquitin quantification

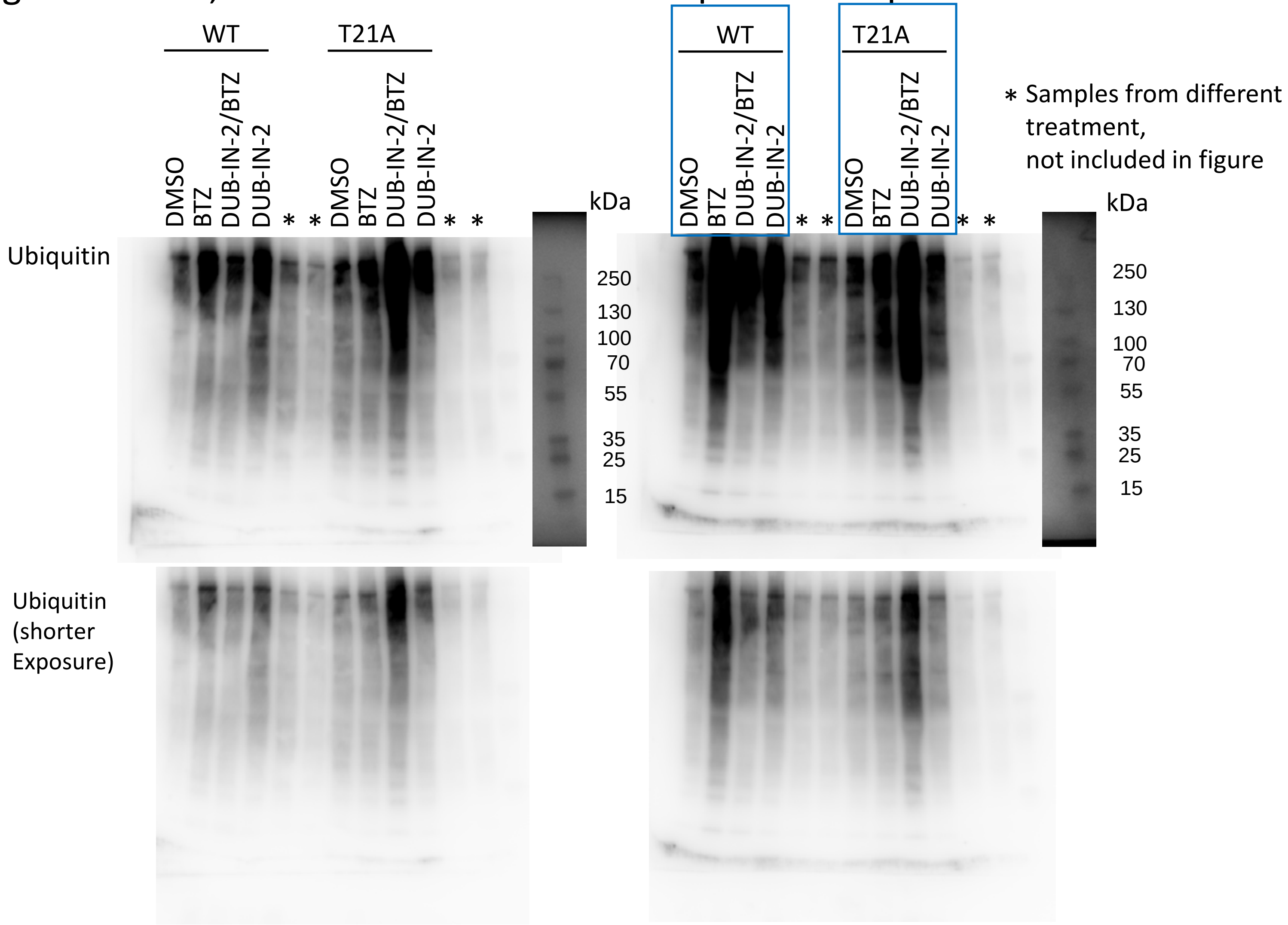

Fig. 4E KMS18, 24h Western blots: 2 independent experiments shown for ubiquitin quantification

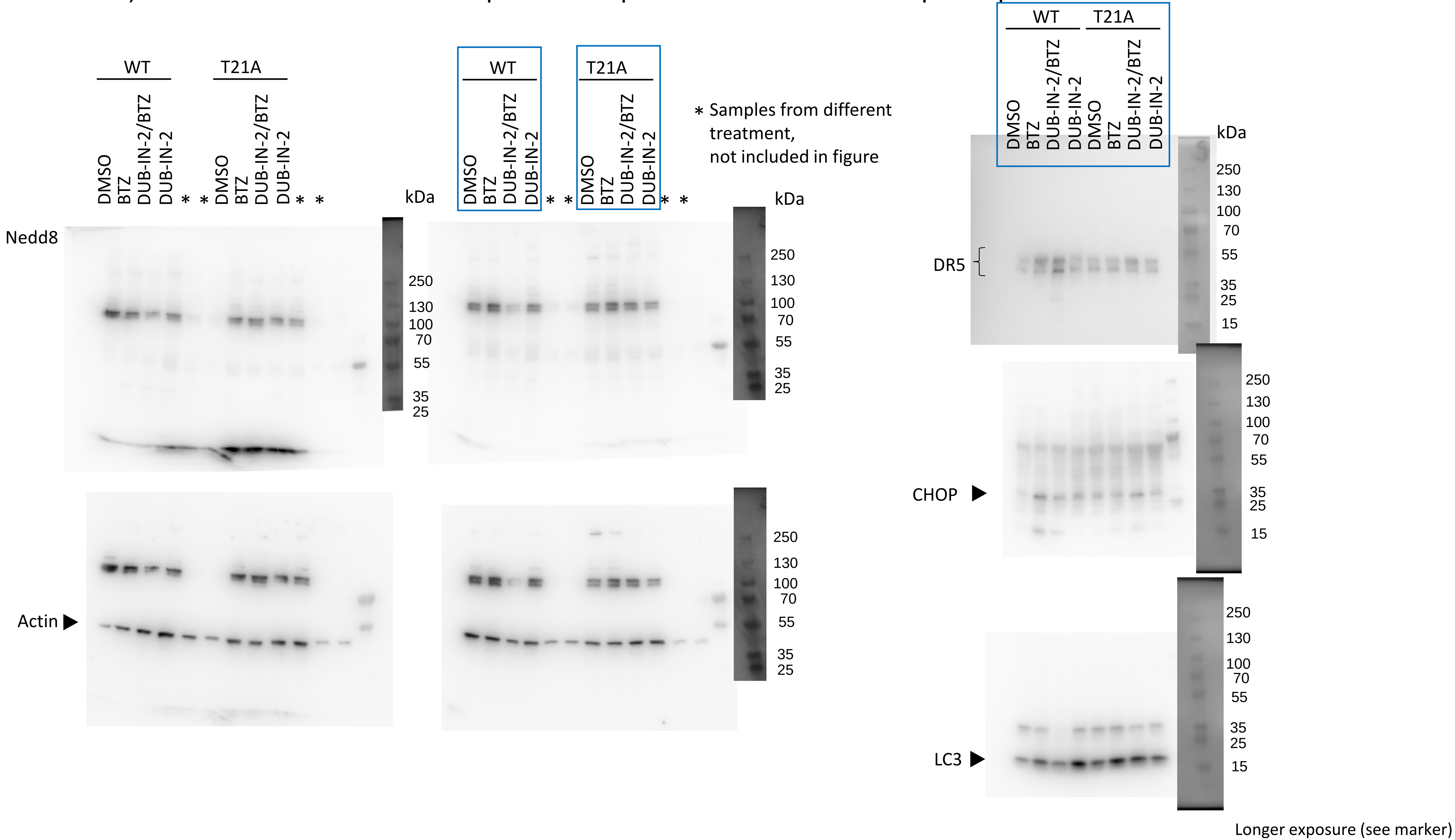

Fig. 4E KMS18, 24h Western blots: 2 independent experiments

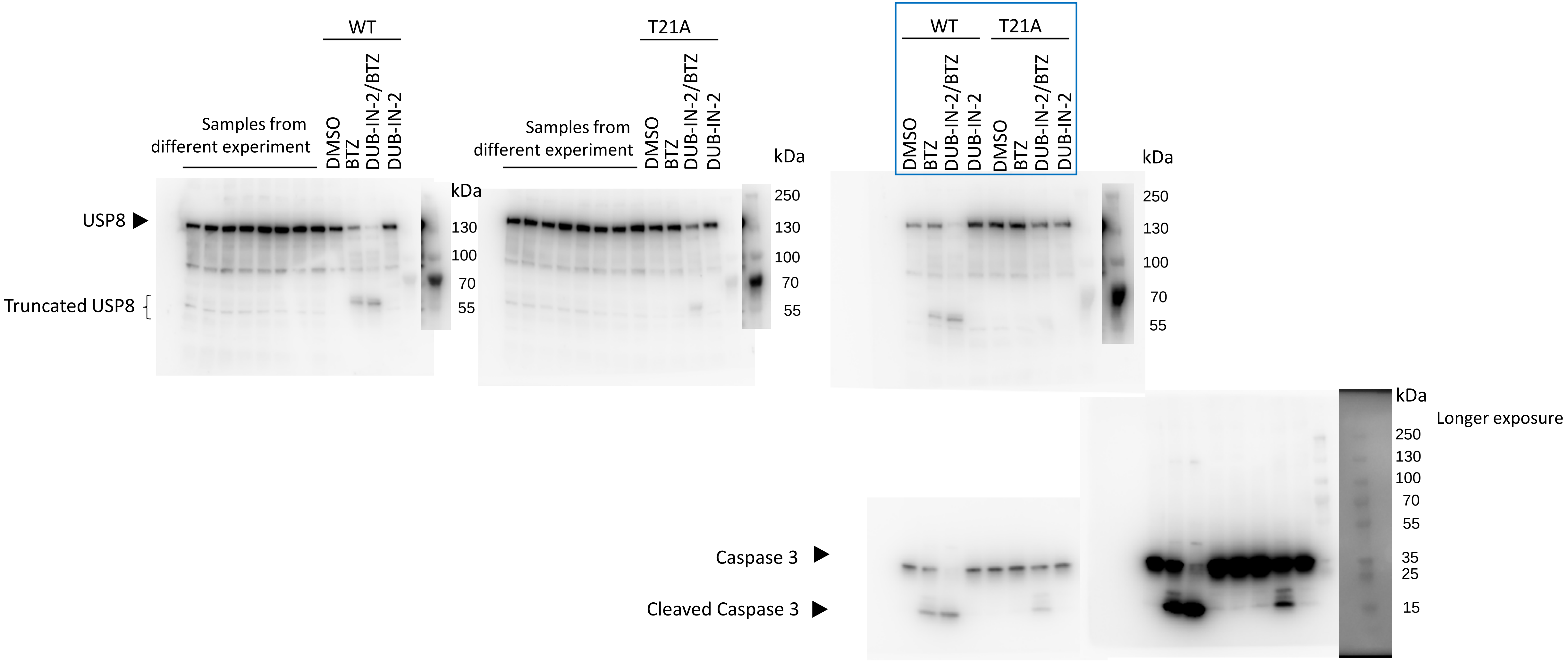

Fig. 4F KMS27-6h Western blots: 2 independent experiments

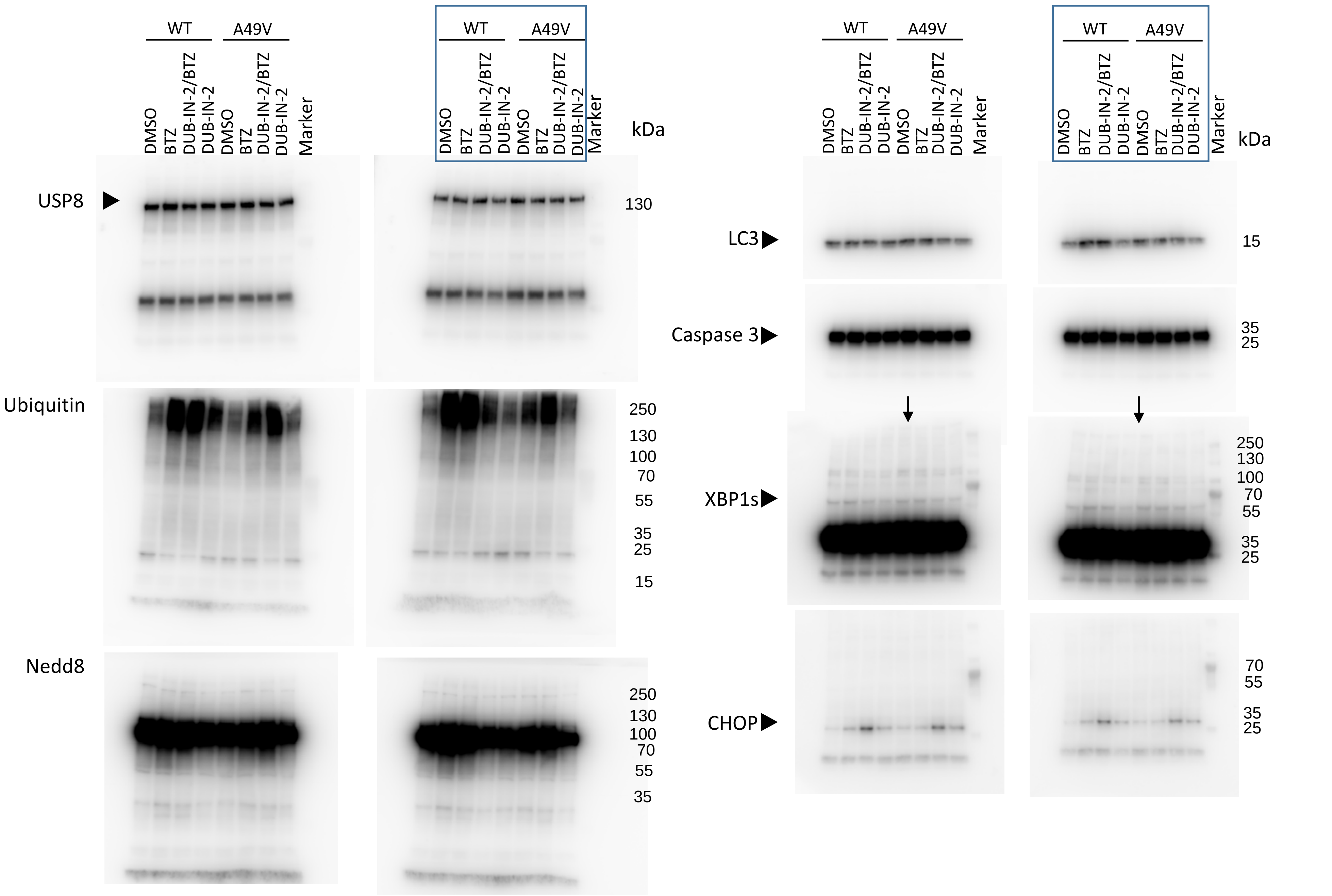

Fig. 4F KMS27-6h   Western blots: 2 independent experiments

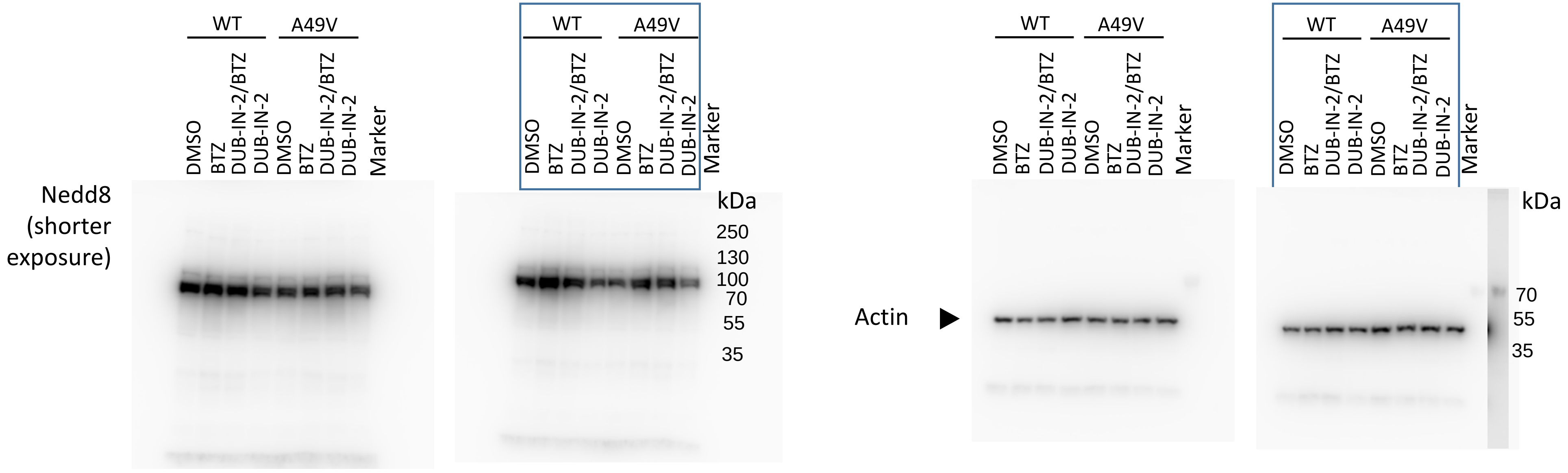

Fig. 4F KMS27-24h Western blots: 2 independent experiments

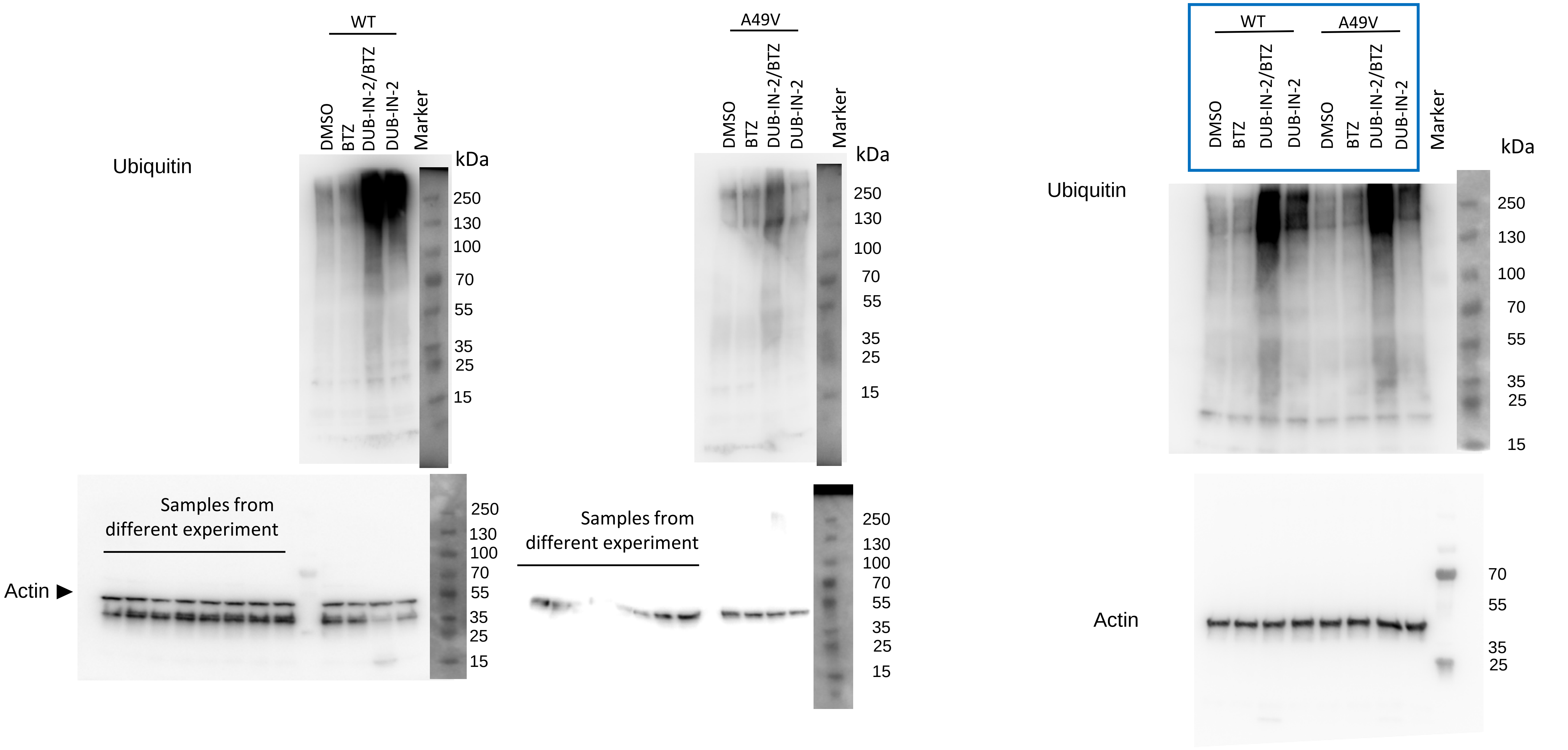

Fig. 4F KMS27-24h Western blots: 2 independent experiments

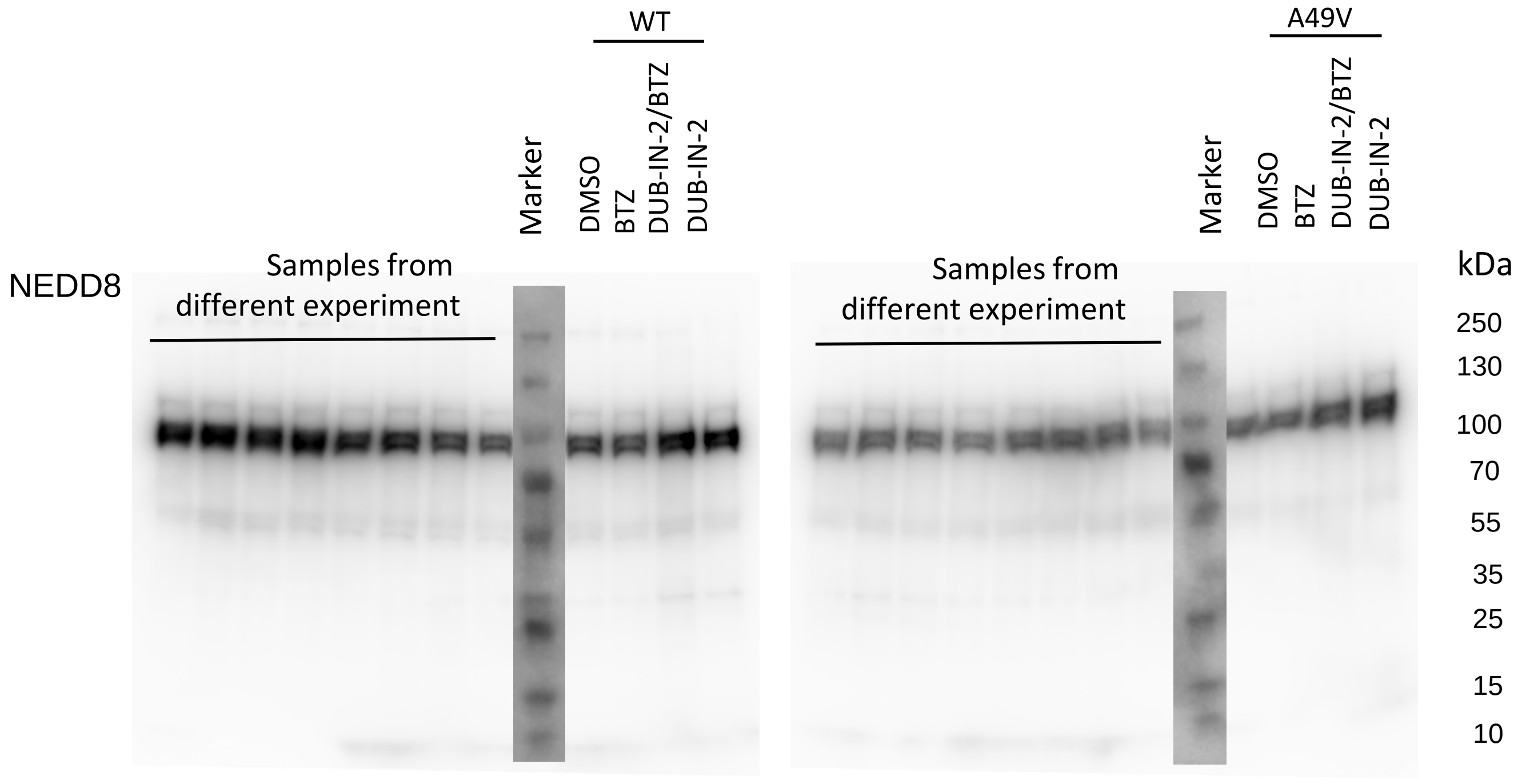

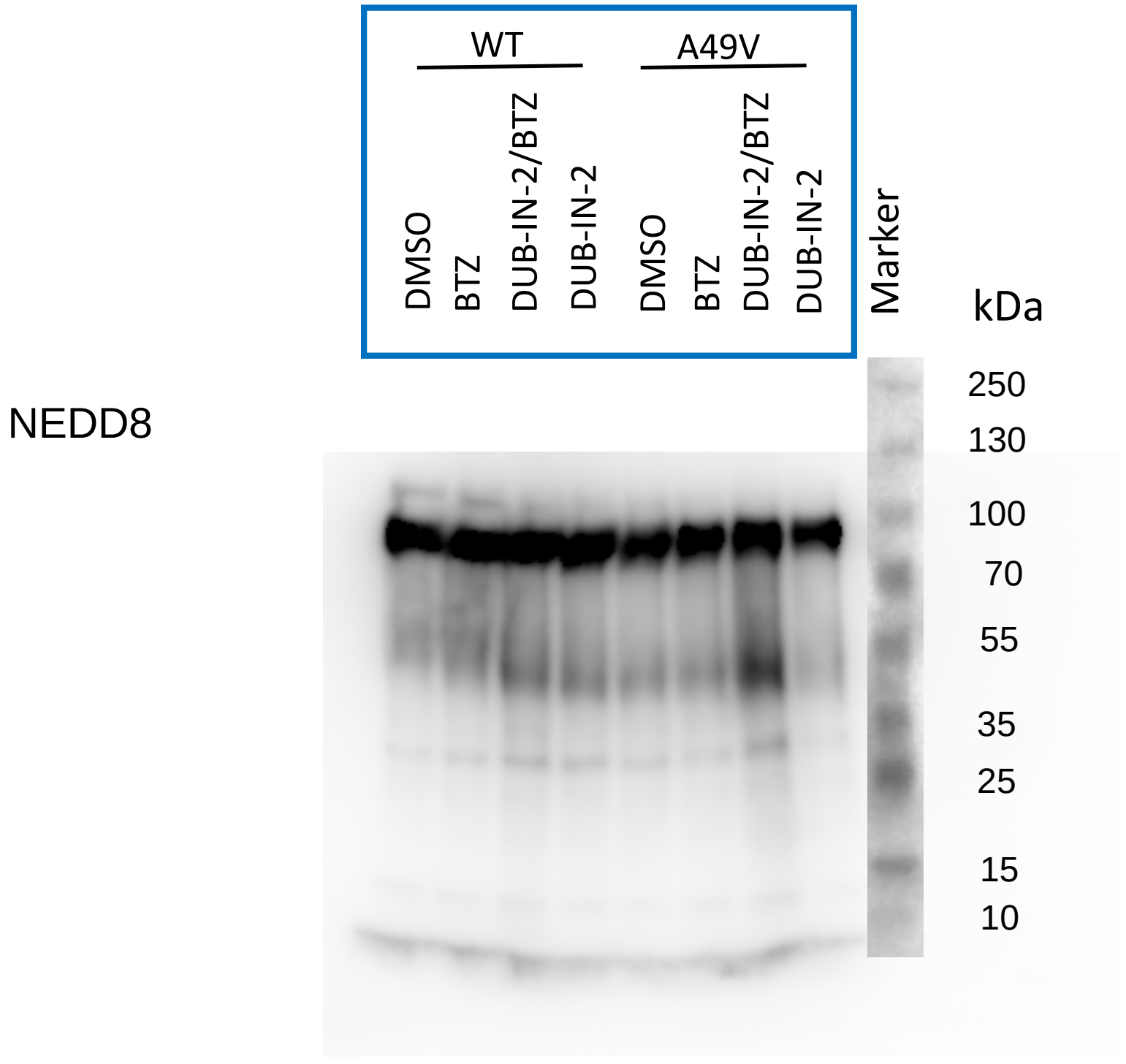

Fig. 4F KMS27-24h Western blots: 2 independent experiments

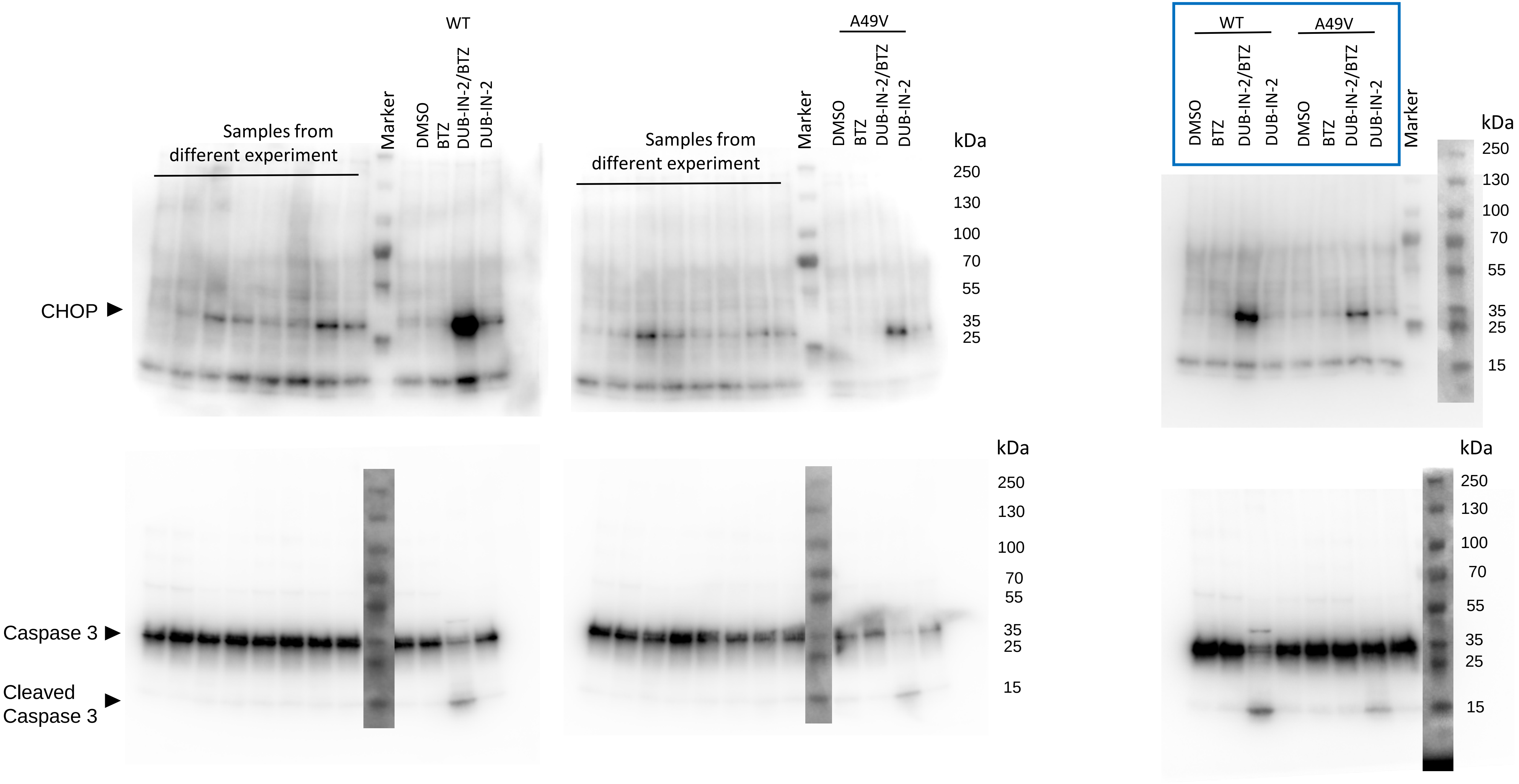

Fig. 4F KMS27-24h Western blots: 2 independent experiments

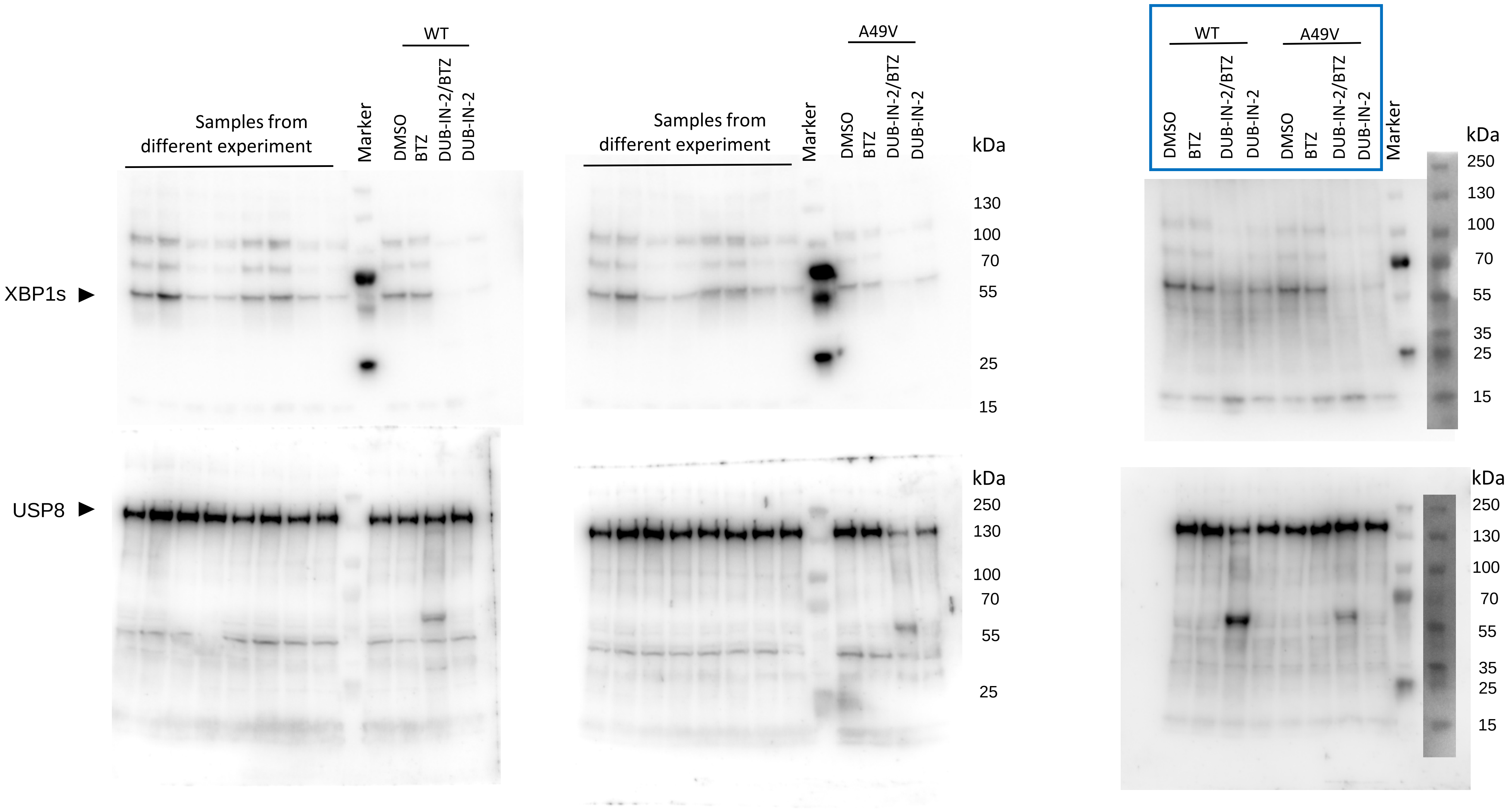

Fig. 4F KMS27-24h Western blots: 2 independent experiments

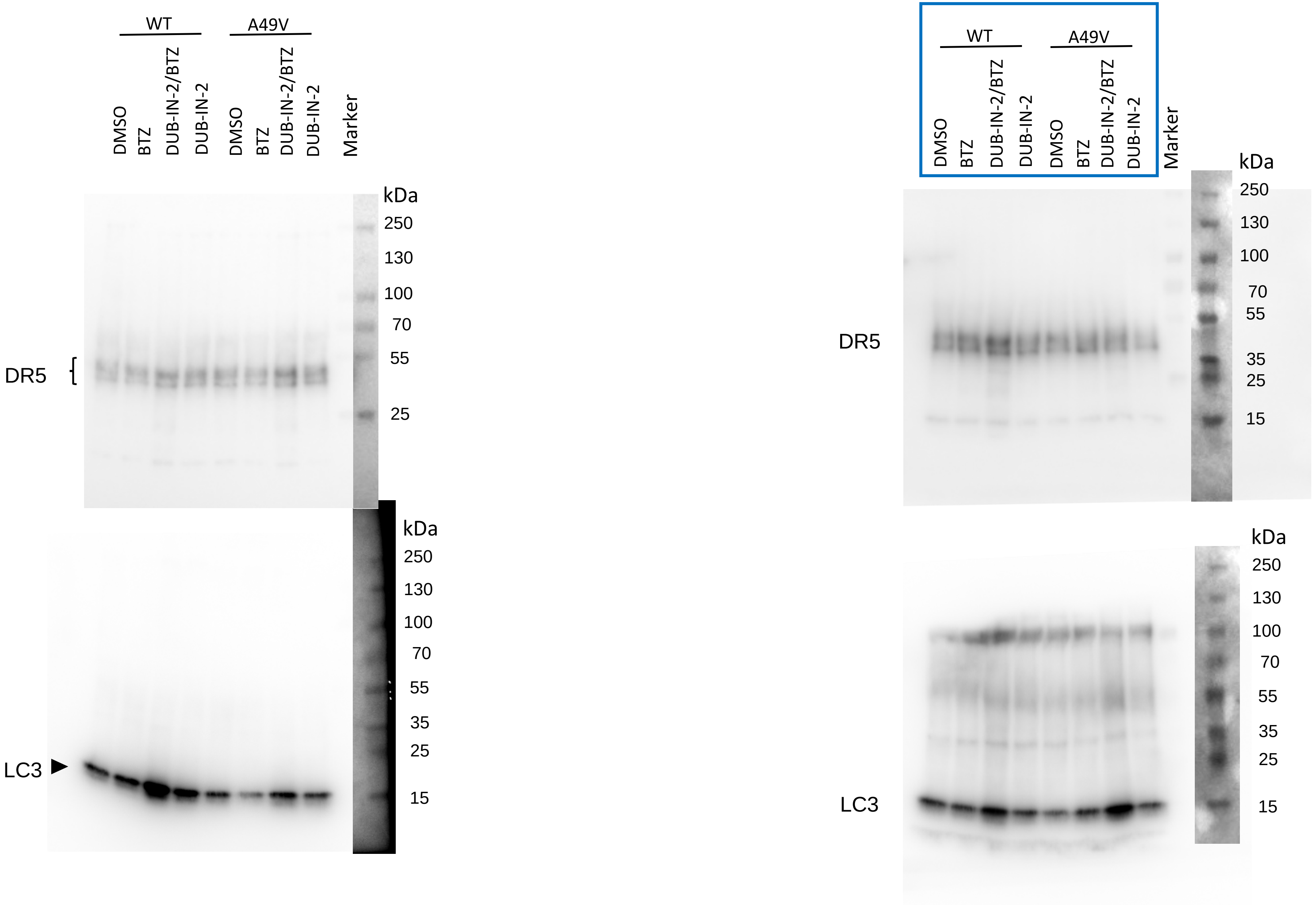

Fig. 5B KMS18+KMS27, USP8 siRNA+/-6h BTZ   Western blots: 2 different cell lines

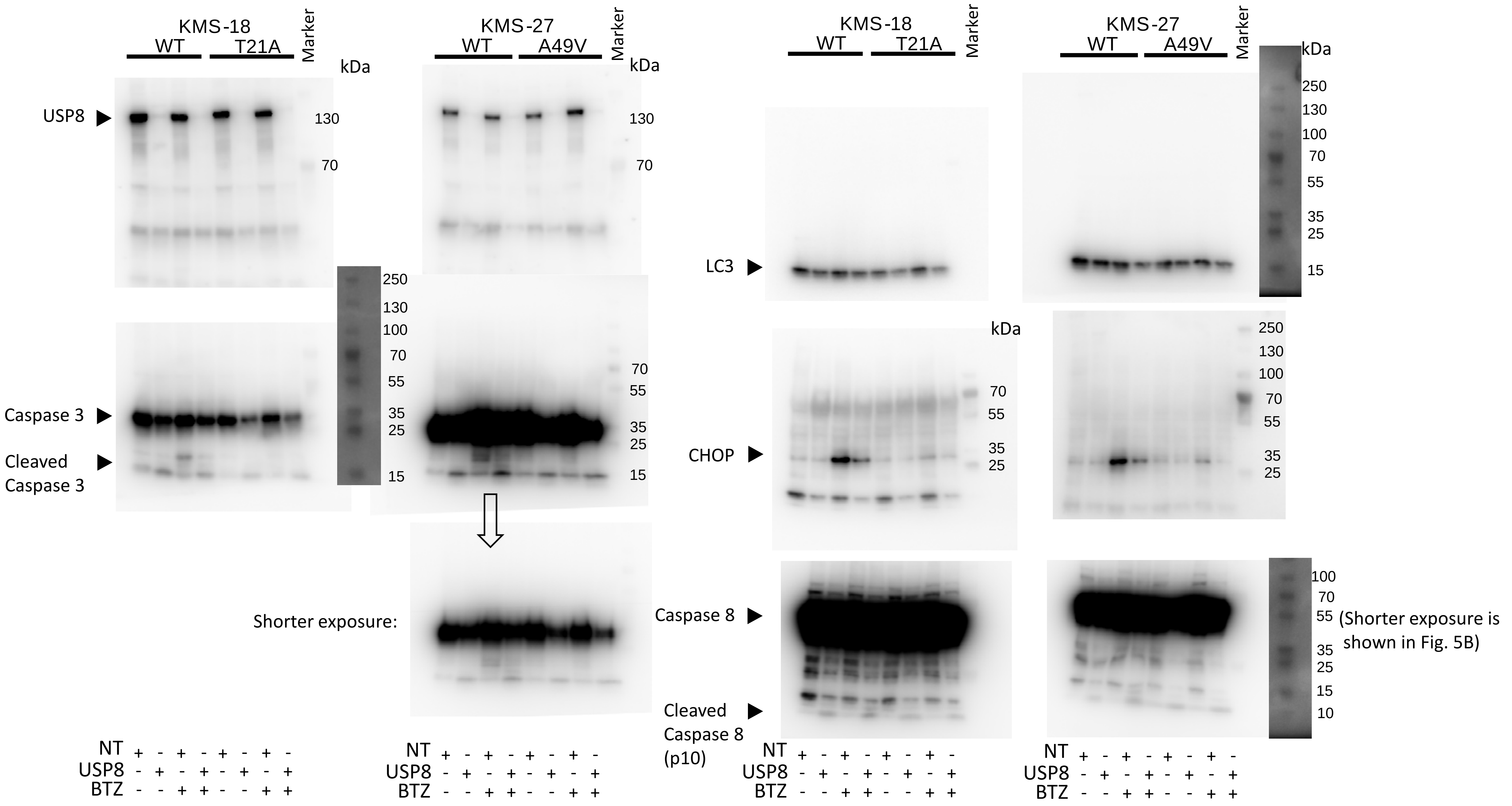

Fig. 5B KMS18+KMS27, USP8 siRNA+/-6h BTZ

Western blots: 2 different cell lines

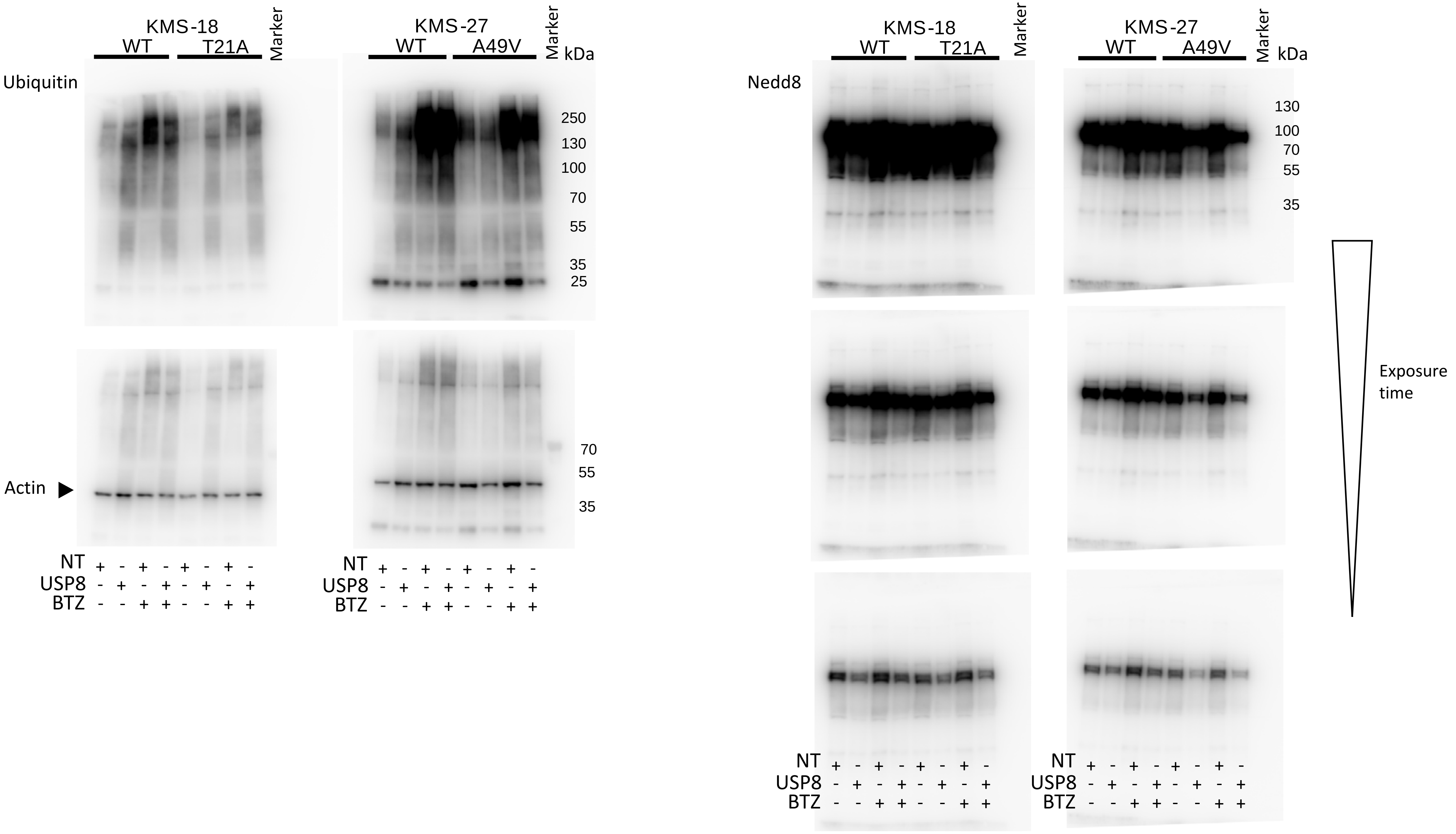

Fig. 5B KMS18+KMS27, USP8 siRNA+/-6h BTZ

(Additional/duplicate experiments, WBs used for quantification of ubiquitylation)

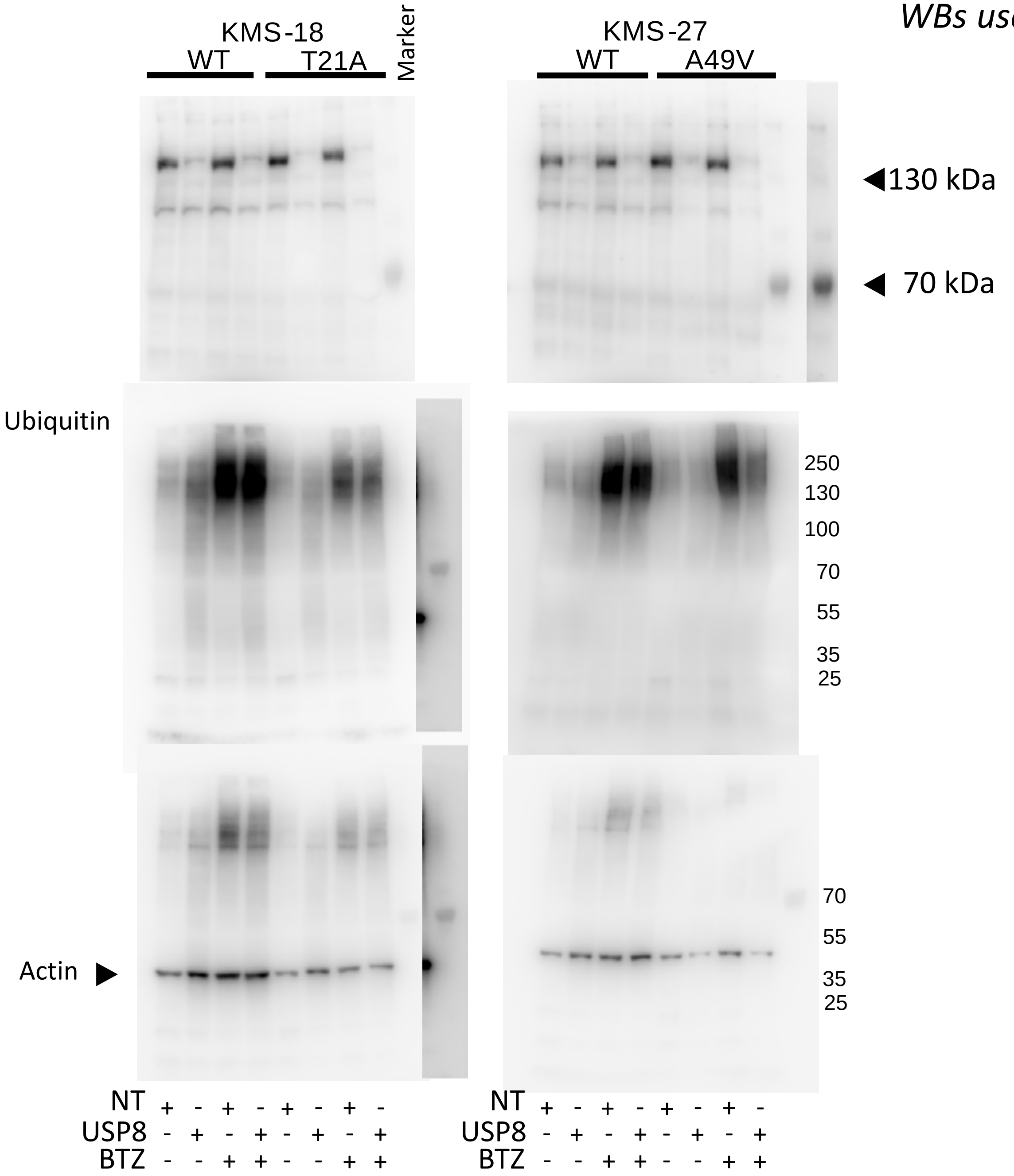

Fig. 5D KMS18 T21A cells: USP8 siRNA, +/- BTZ, DUB-IN-2, or BTZ/DUB-IN-2

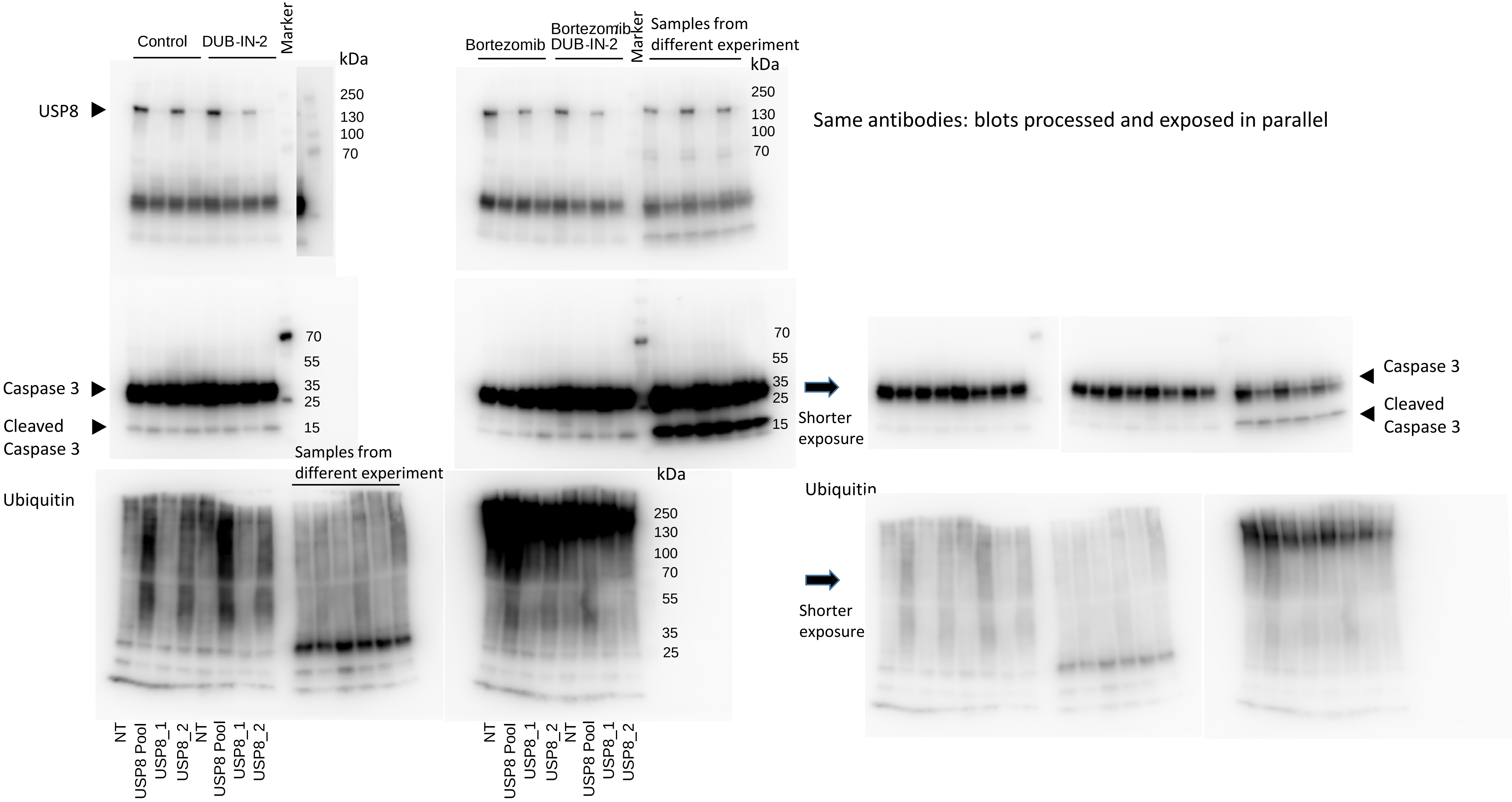

Fig. 5D KMS18 T21A cells: USP8 siRNA, +/- BTZ, DUB-IN-2, or BTZ/DUB-IN-2

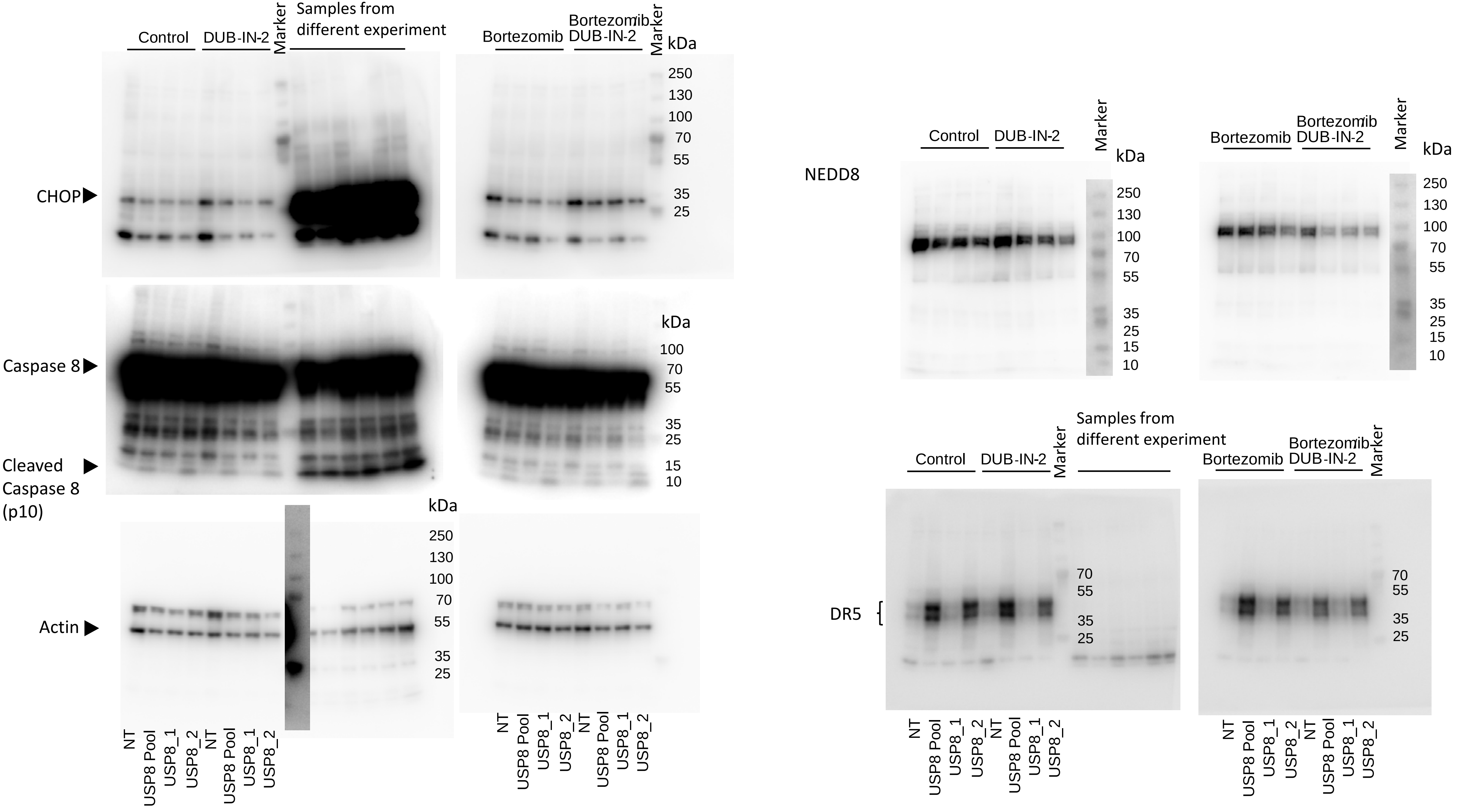

Fig. 5D KMS18 T21A cells: USP8 siRNA, +/- BTZ, DUB-IN-2, or BTZ/DUB-IN-2

Additional independent experiment: WBs used for quantification of ubiquitylation

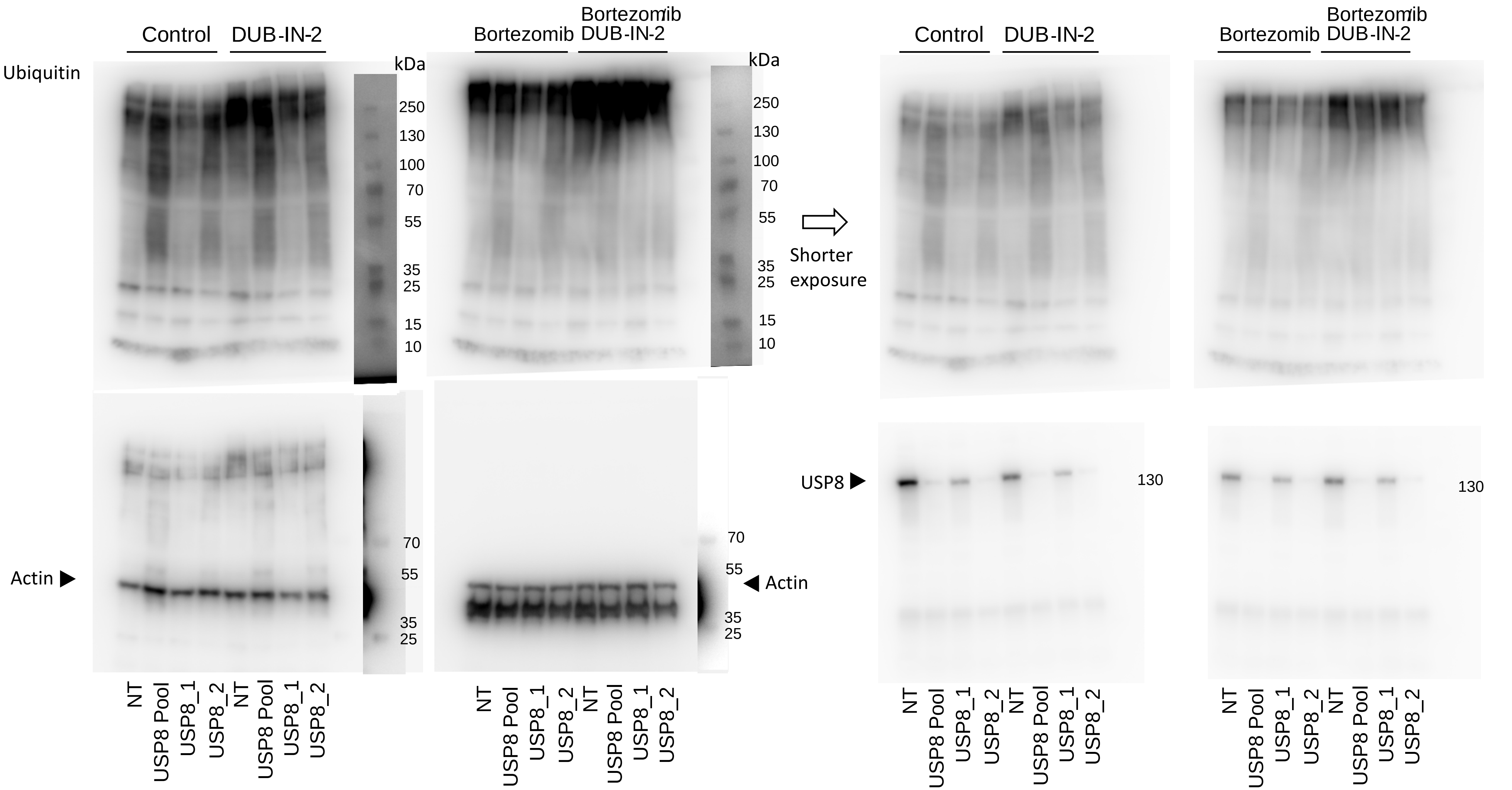

Fig. 5E HEK293T cells: siRNA rescue experiment: 2 independent experiments

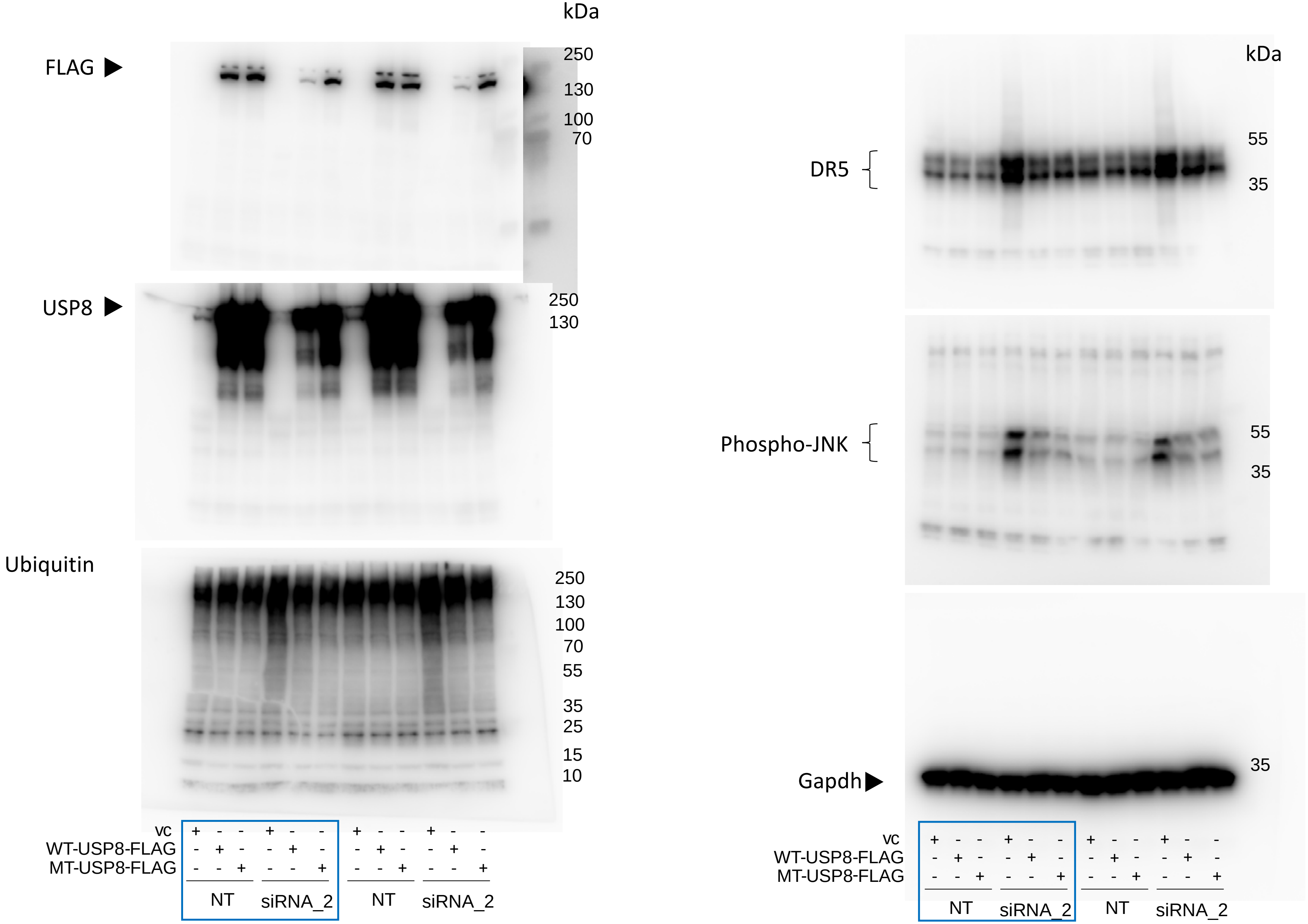

Fig. 5F KMS-18 T21A cells: CHOP knockdown, 2 independent experiments

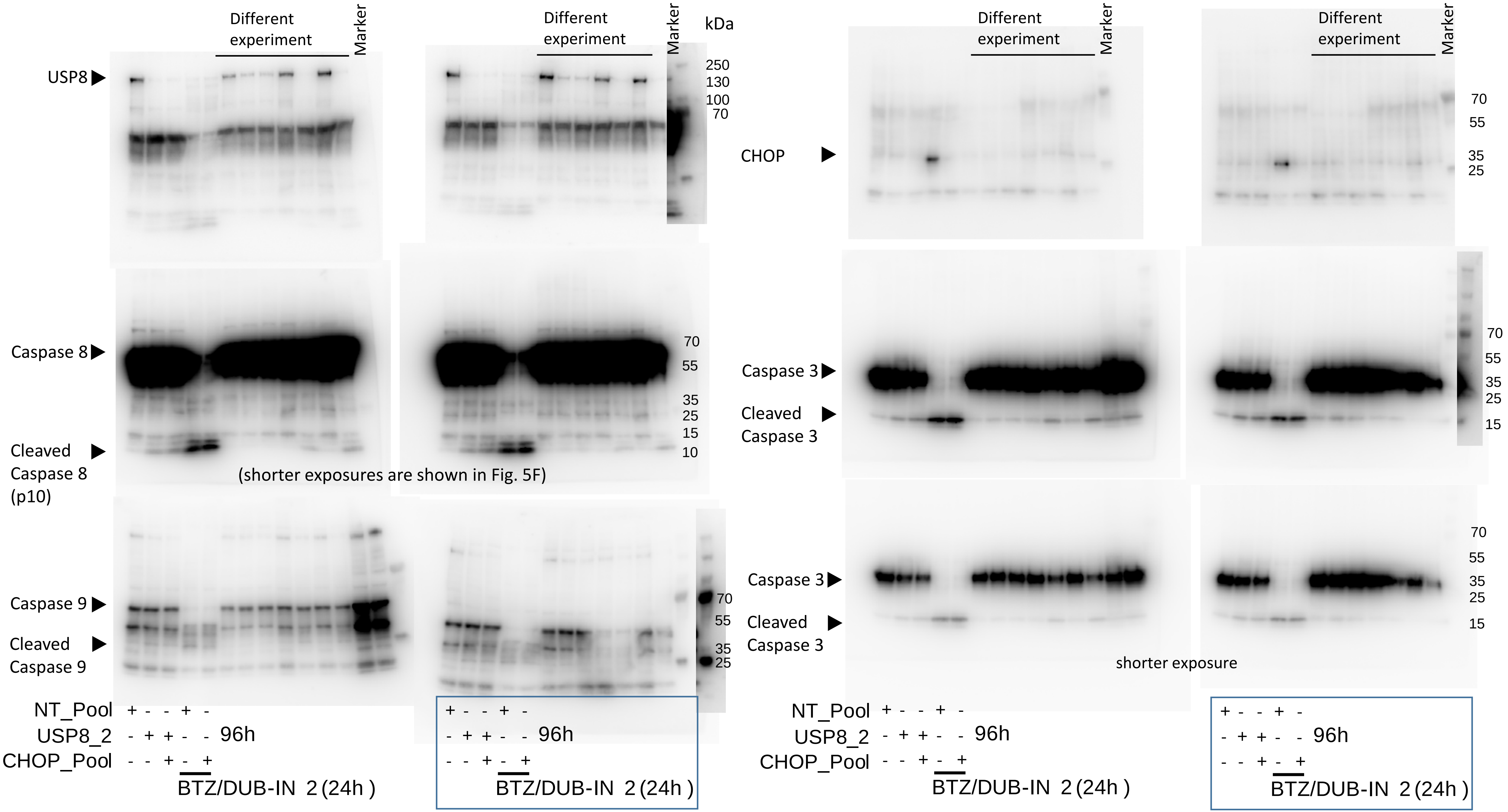

Fig. 5F

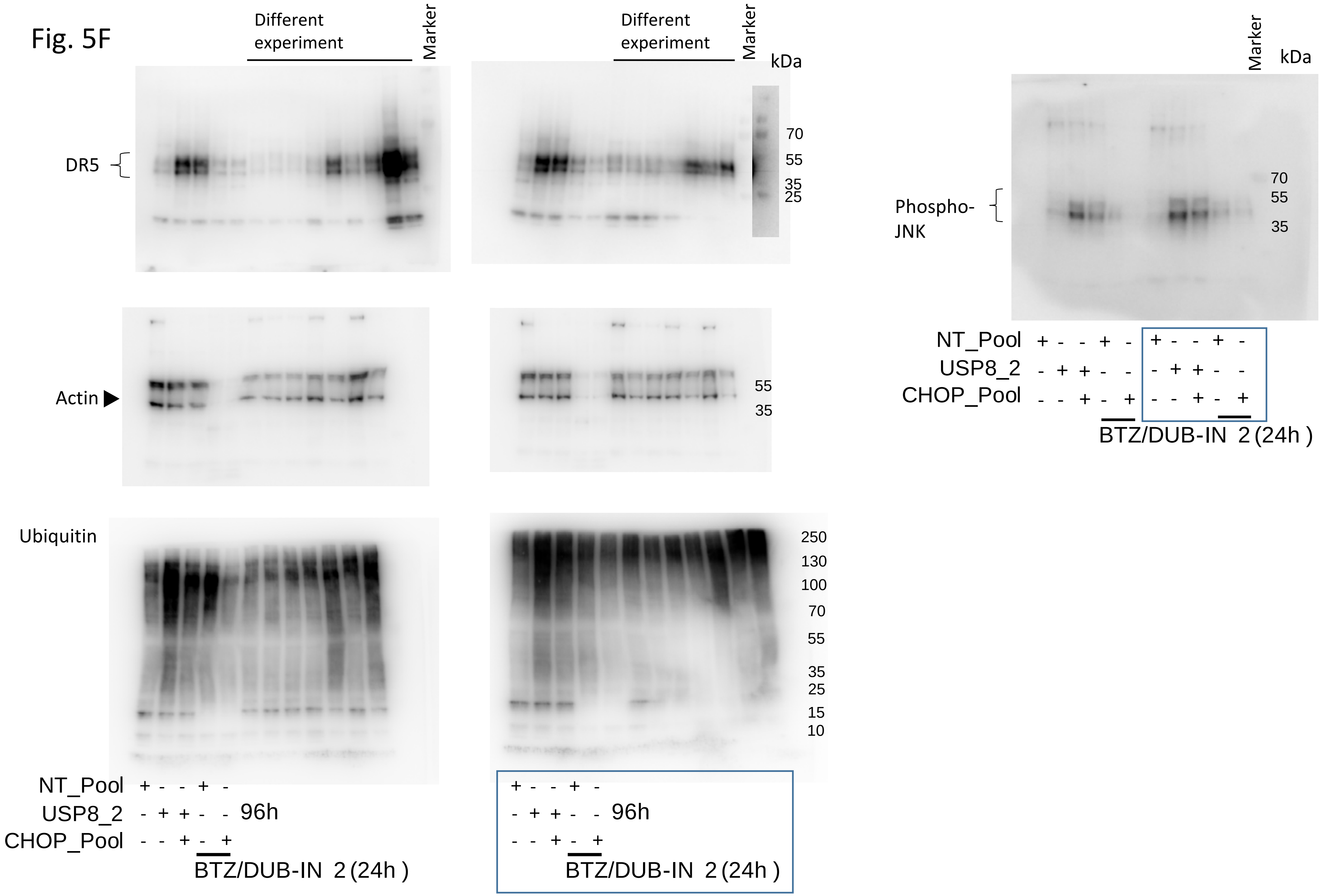
Different experiment
Marker
kDa
DR5
70
55
35
25
Actin
55
35
Ubiquitin
250
130
100
70
55
35
25
15
10
NT_Pool + - - + -
USP8_2 - + + - - 96h
CHOP_Pool - - + - +
BTZ/DUB-IN 2 (24h)
Phospho-JNK
70
55
35

Fig. 5I KMS-18 T21A cells: Folimycin time course

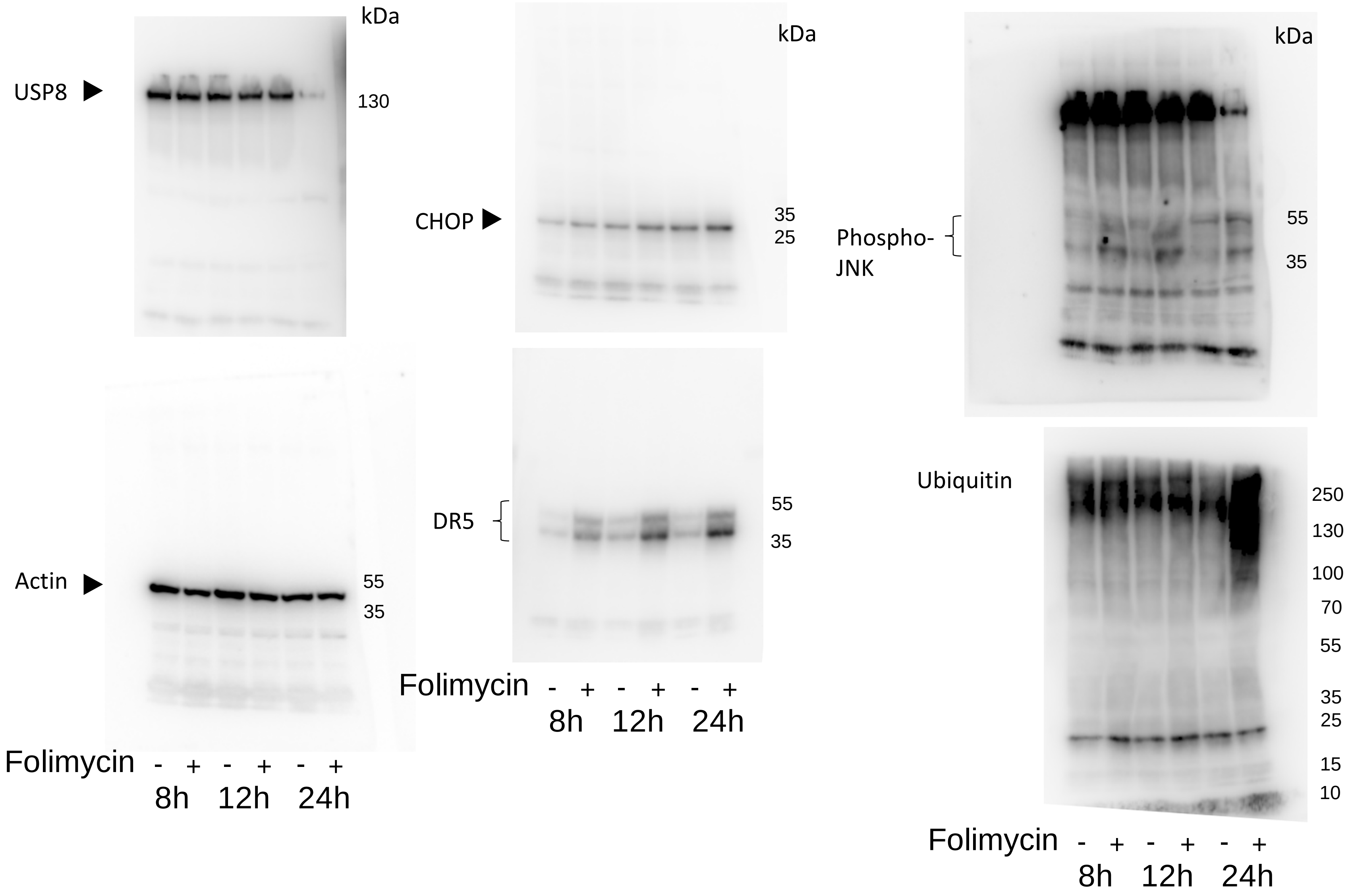

Fig. 5J KMS-18 T21A cells: Bafilomycin/inhibitor treatment, 2 independent experiments

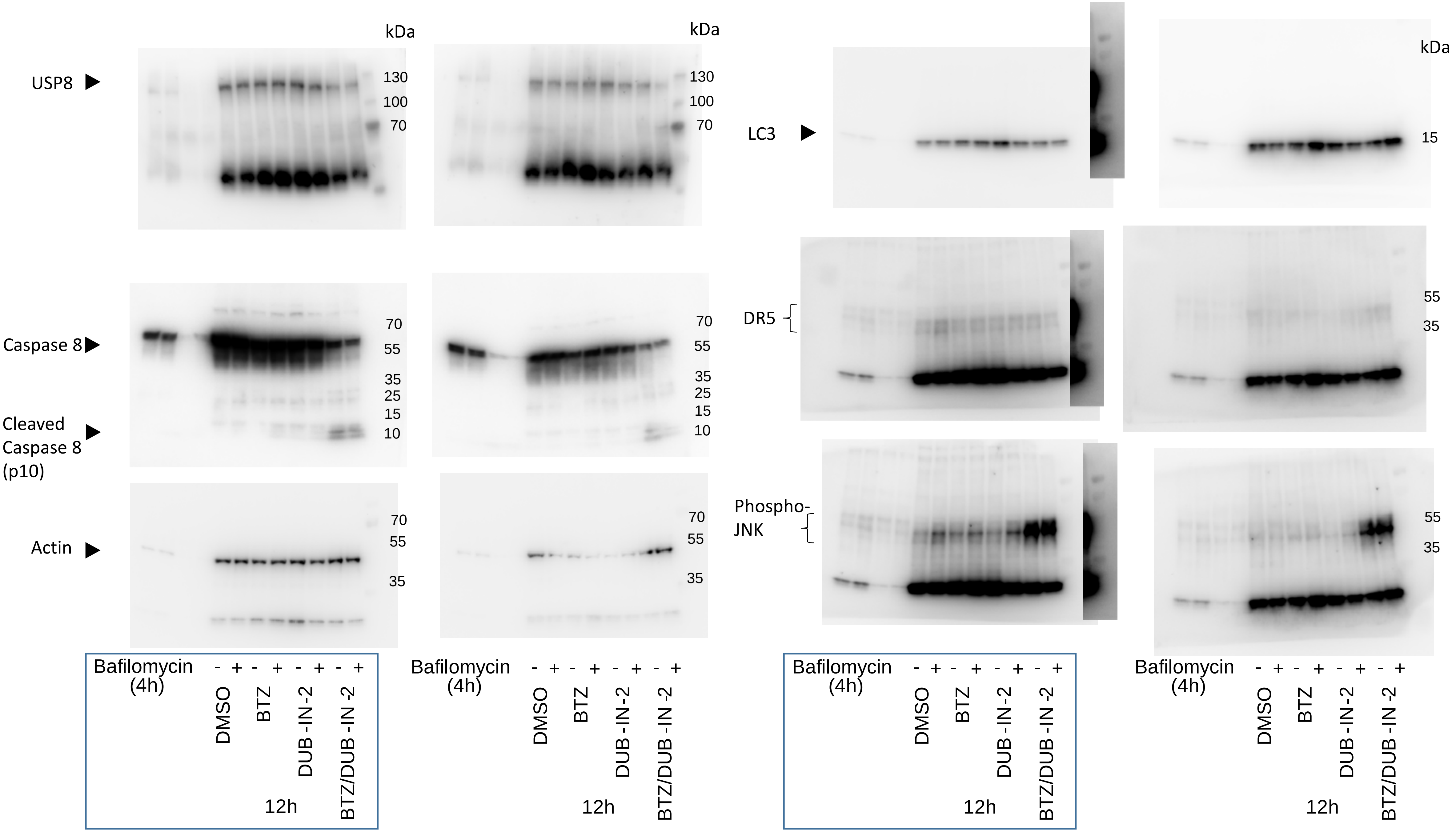

Fig. S1A Western Blots: GADS expression in T and B cells

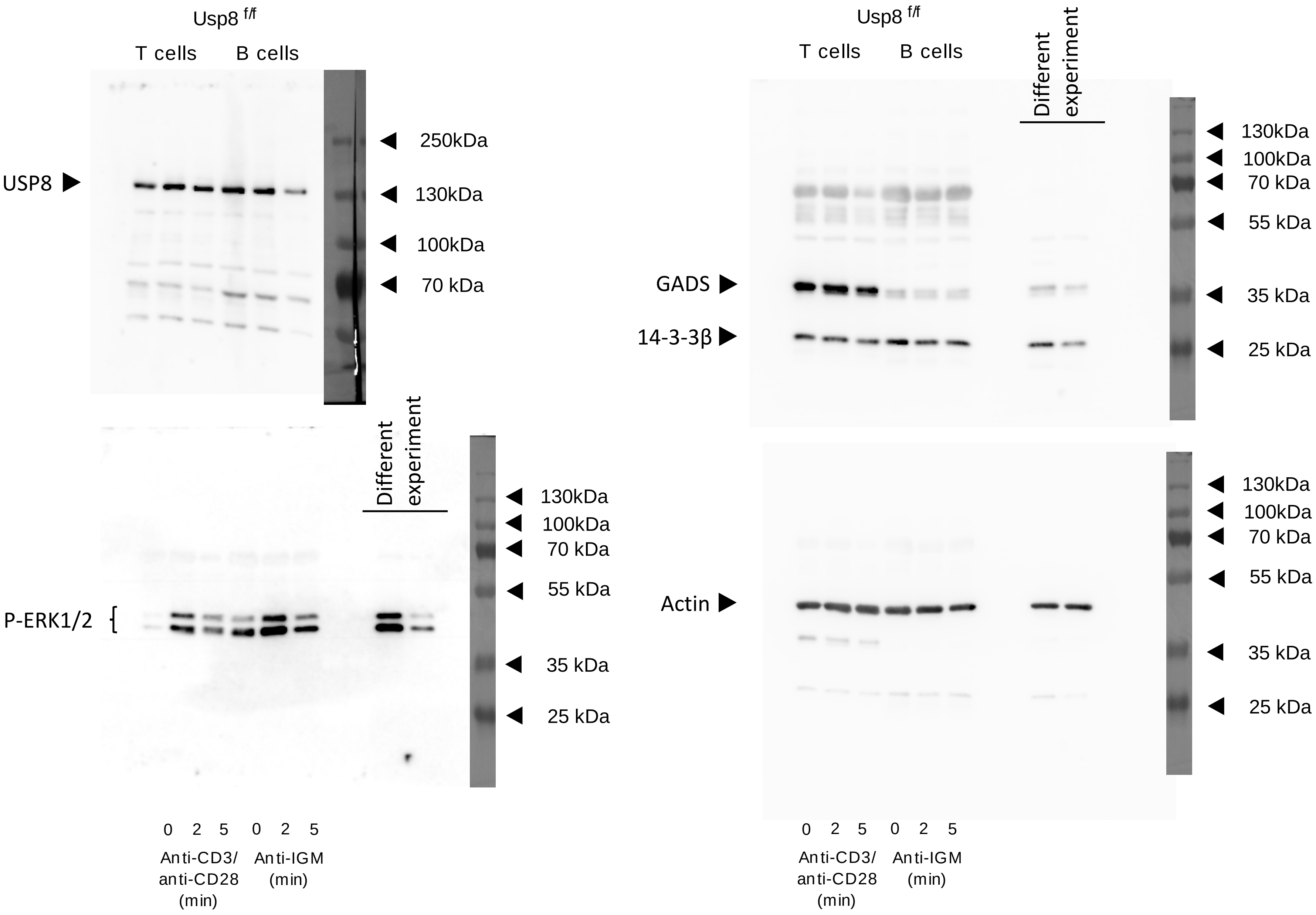

Fig. S3A Western Blots: Induced USP8 deletion in B cells: 48 and 72 hours post OHT

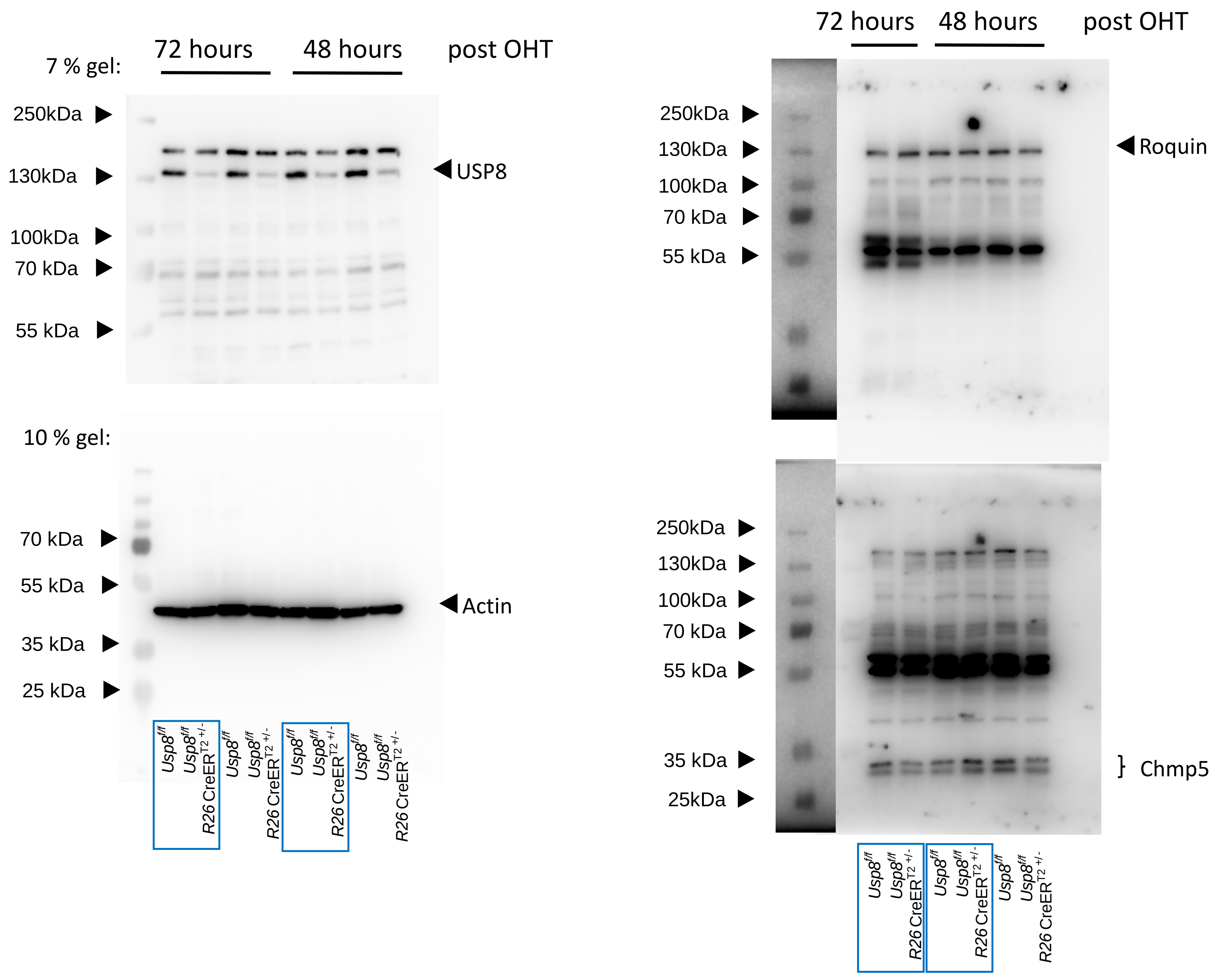

Fig. S3D

Tyrosine phosphorylation in $Usp8^{f/f}$ and $Usp8^{f/f}$Cd19Cre B cells

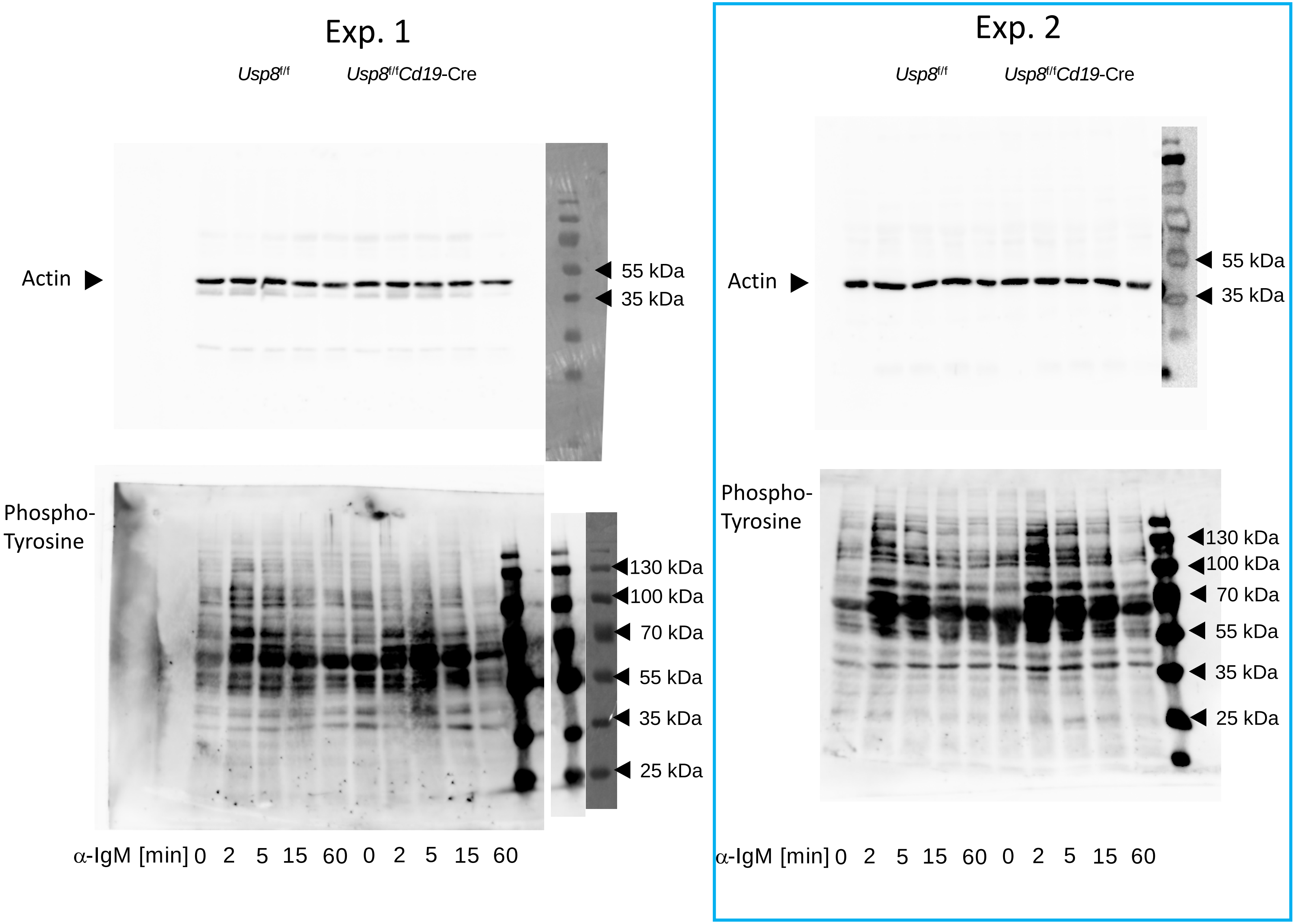

Fig. S3E JNK activation upon 48h USP8 depletion in USP8$^{f/f}$ R26Cre^{ERT2} B cells, 2 independent experiments

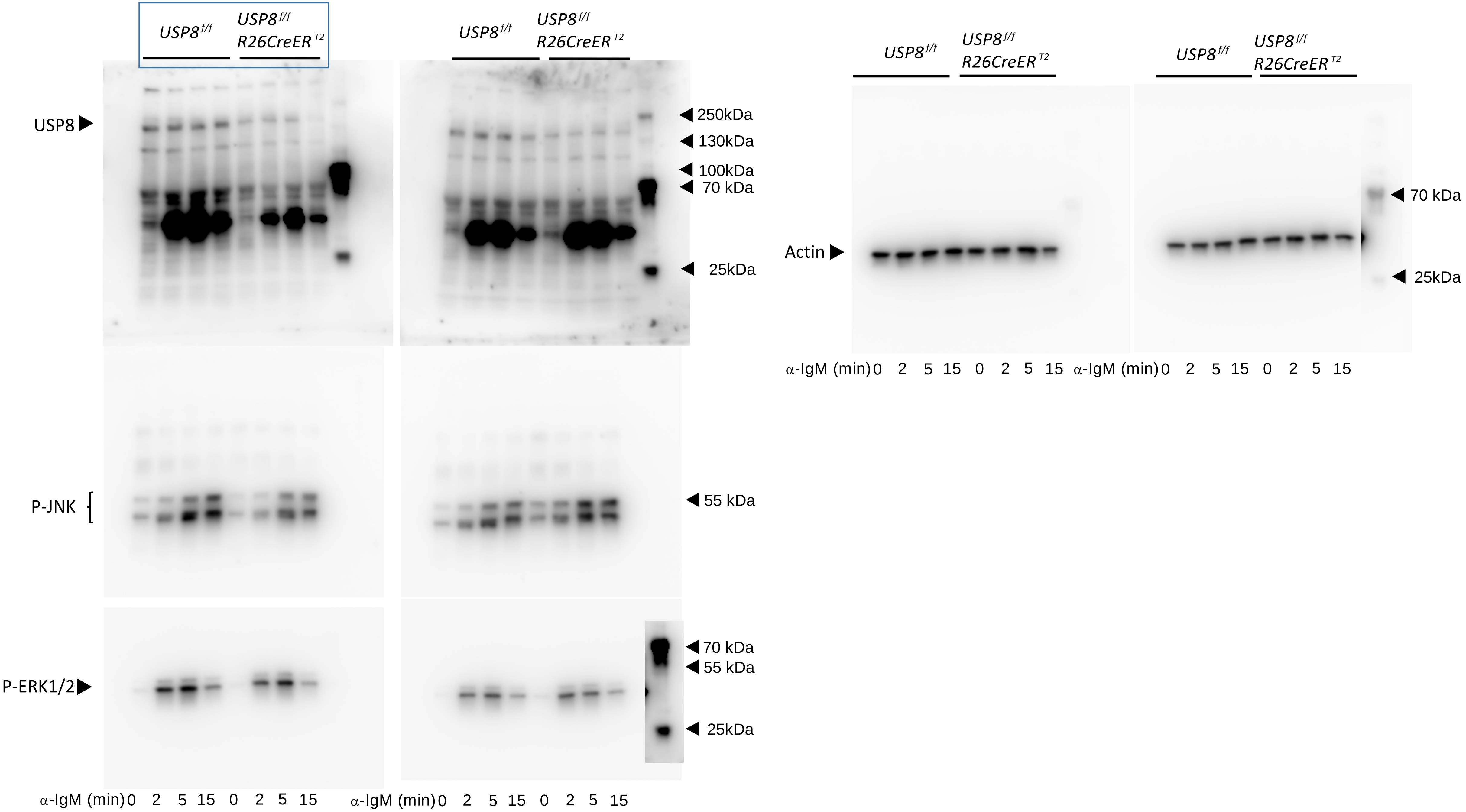

Fig. S4A Effects of NEDD8 K>R mutations on Neddylation/ubiquitylation in the presence of cat. inact. USP8, 2 independent experiments

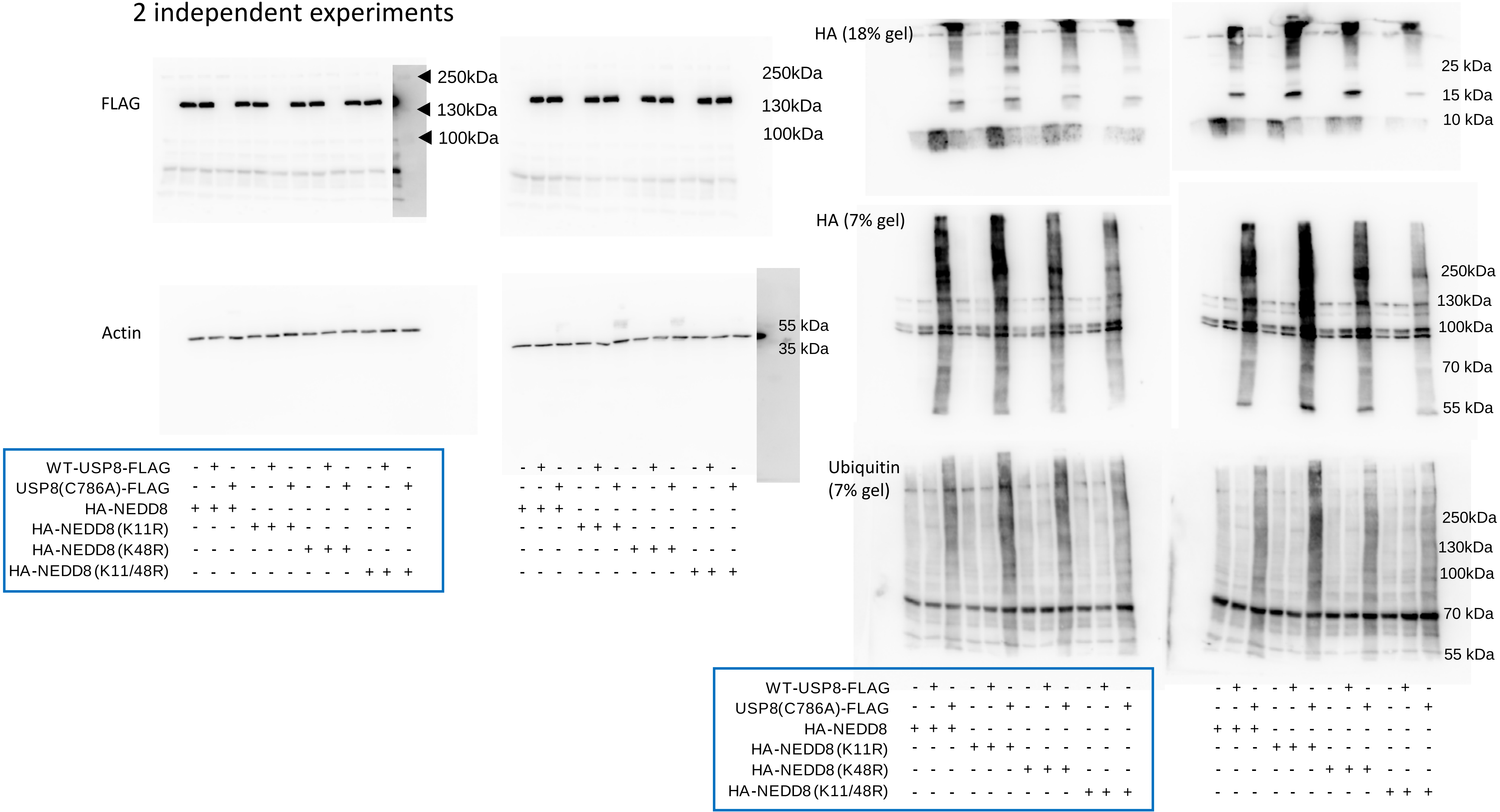

Fig. S4B Effects of NEDD8 overexpression on linkage-specific ubiquitylation/sumoylation in the presence of cat. inact. USP8, 2 independent experiments (same as above)

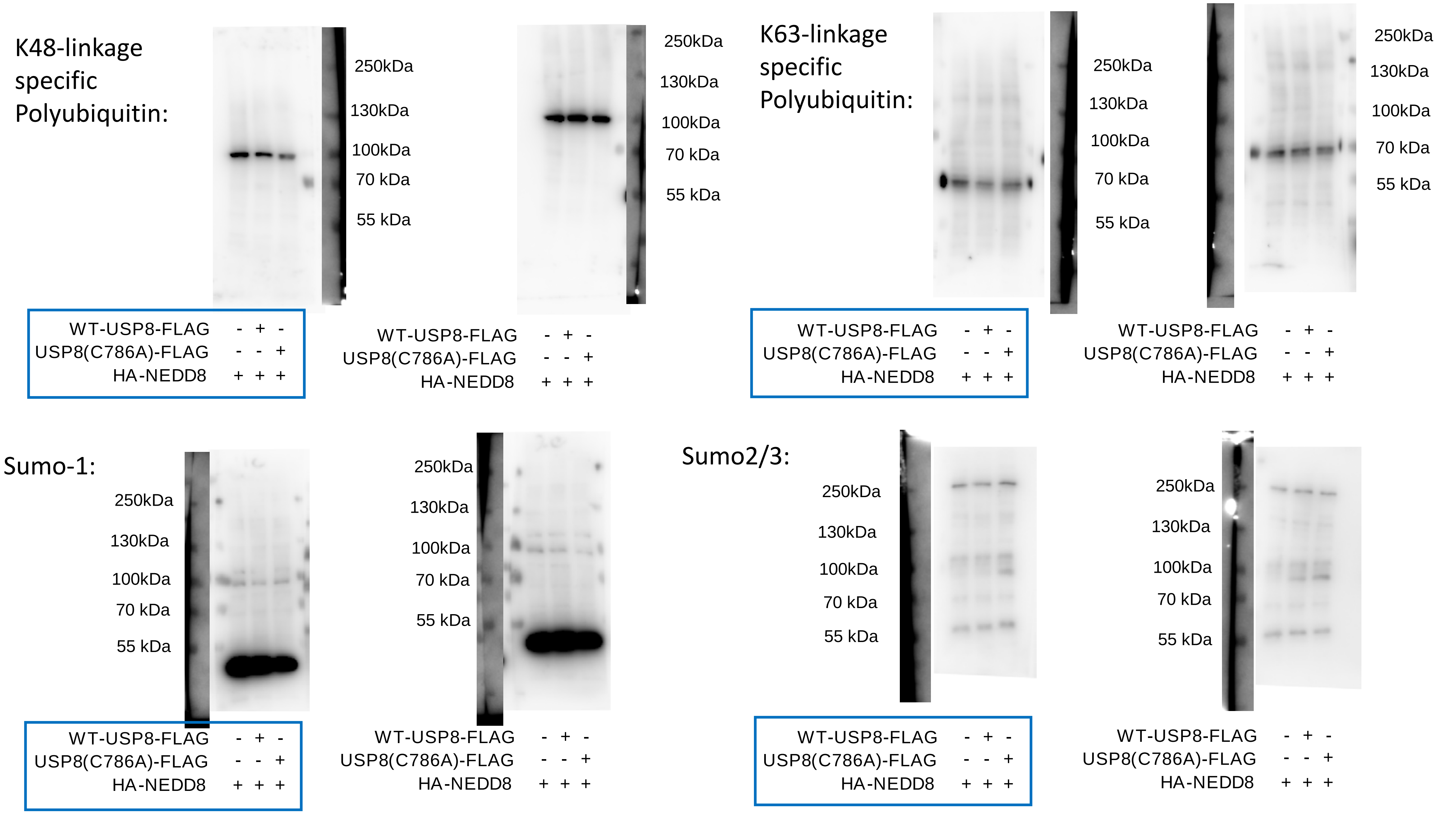

Fig. S5 C   6 h Carfilzomib, KMS27, 2 independent experiments

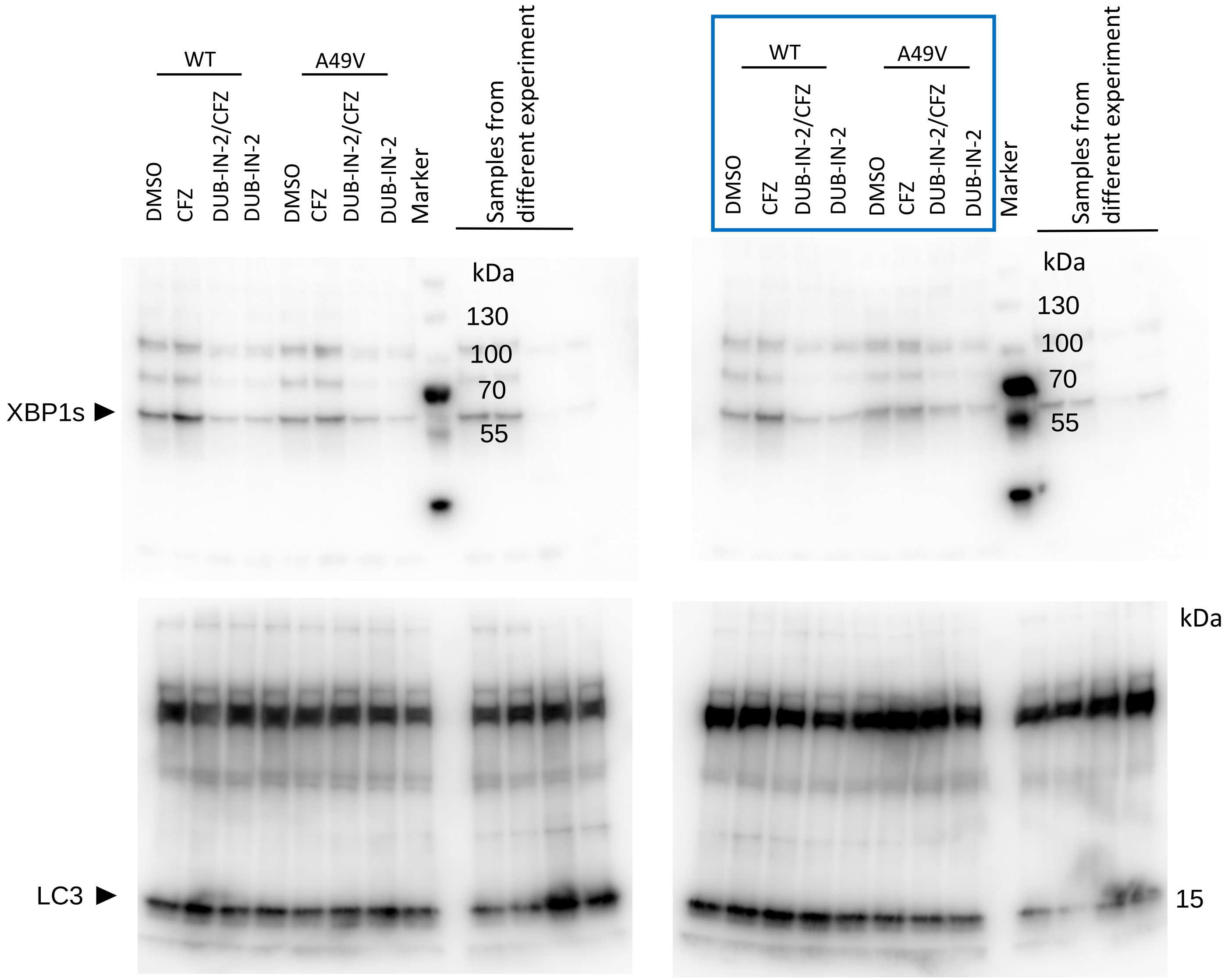

Fig. S5 C 6 h Carfilzomib, KMS27, 2 independent experiments

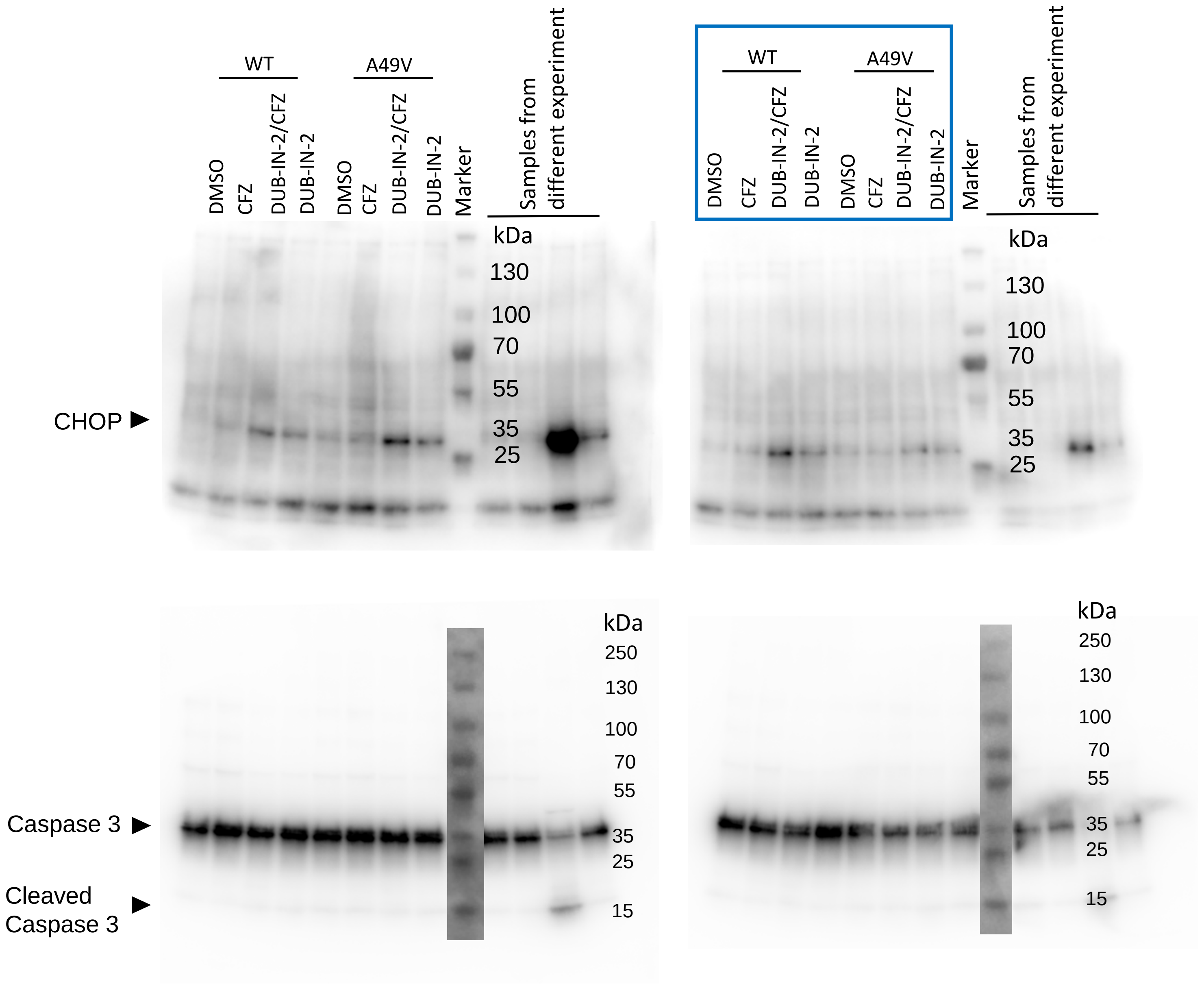

Fig. S5 C 6 h Carfilzomib, KMS27, 2 independent experiments

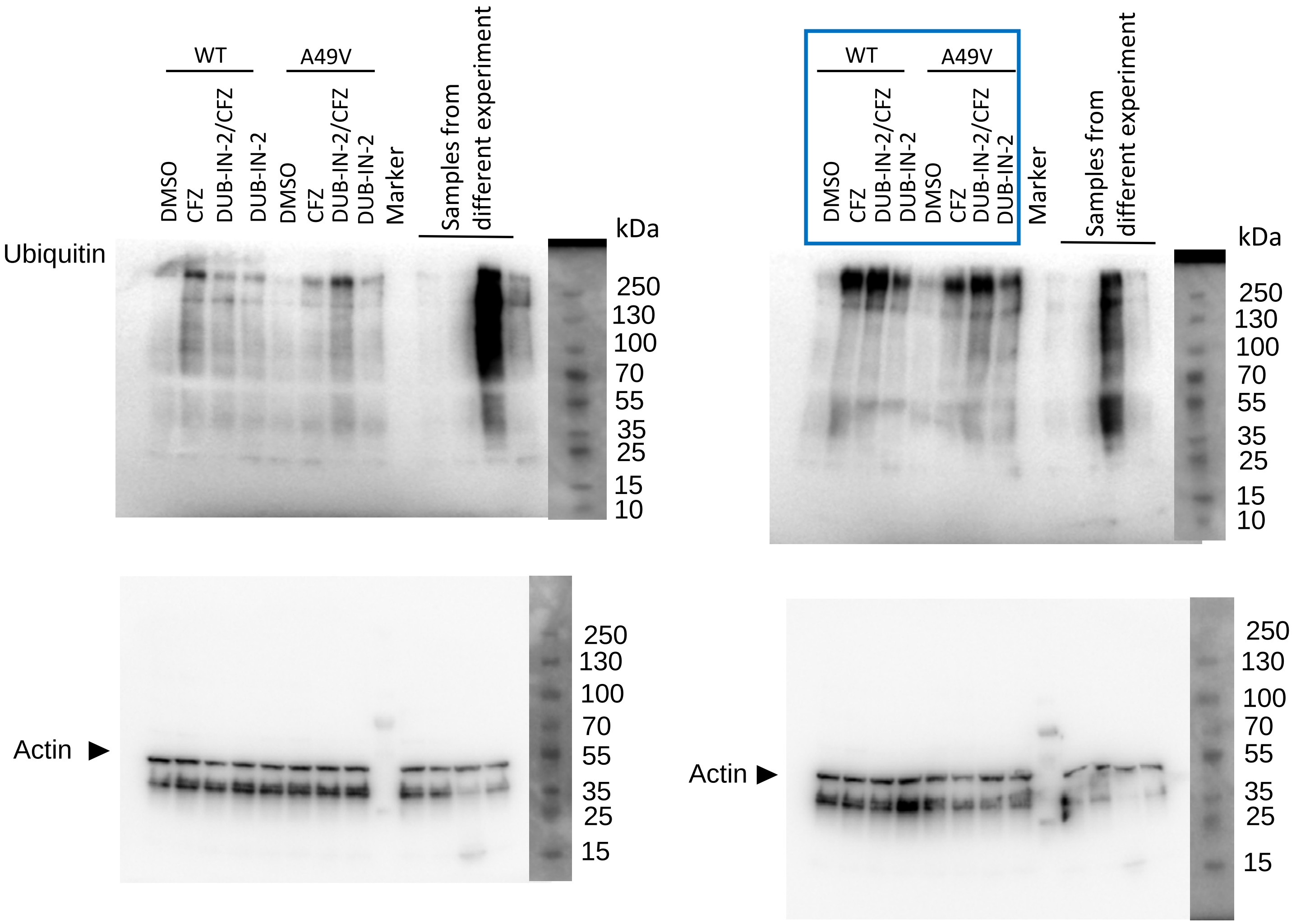

Fig. S5 C 6 h Carfilzomib, KMS27, 2 independent experiments

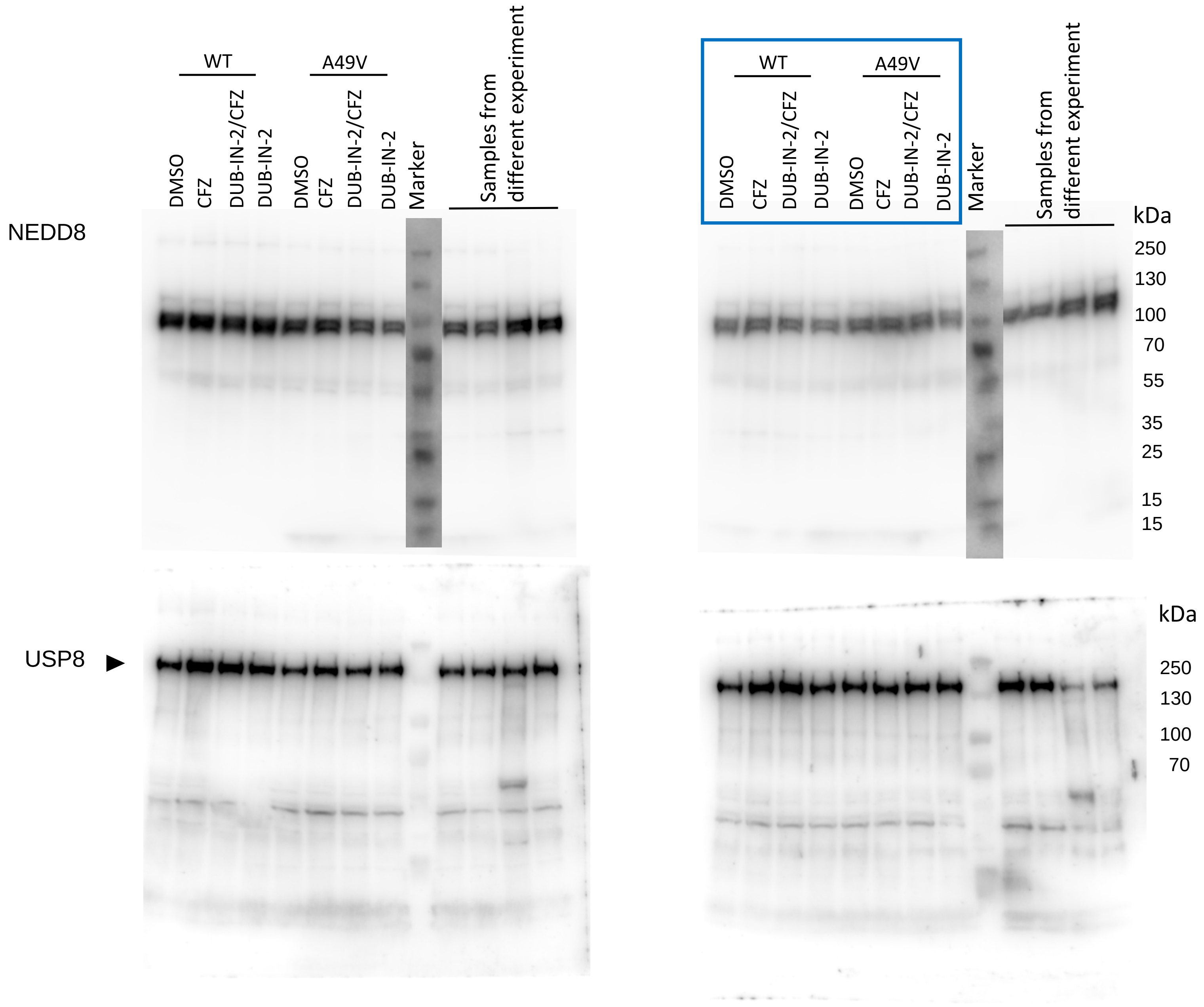

Fig. S6B OTUB1/USP8 inhibitor 61 (OU61): KMS-18-T21A 24 h treatment, duplicate experiments

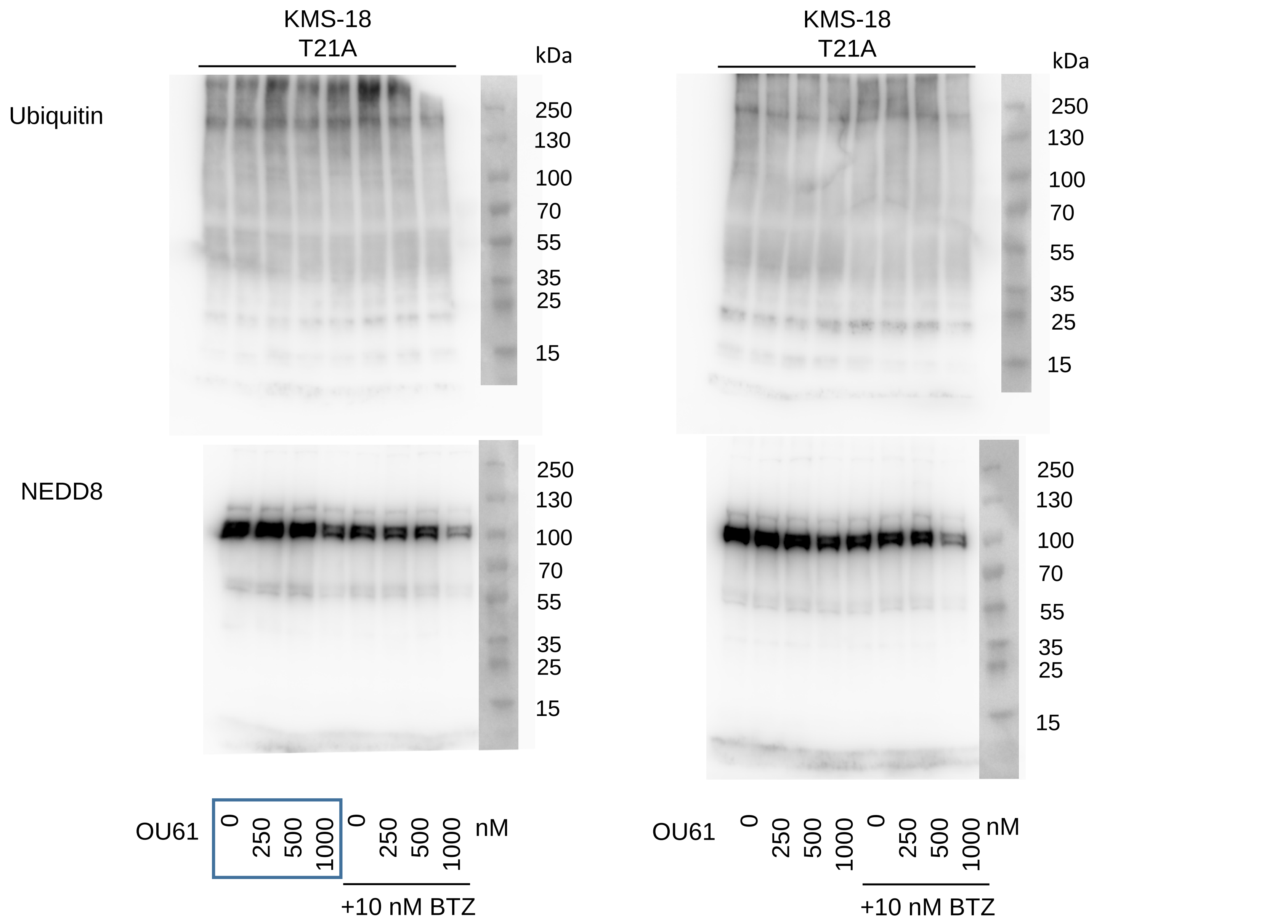

Fig. S6B OTUB1/USP8 inhibitor 61 (OU61): KMS-18-T21A 24 h treatment, duplicate experiments

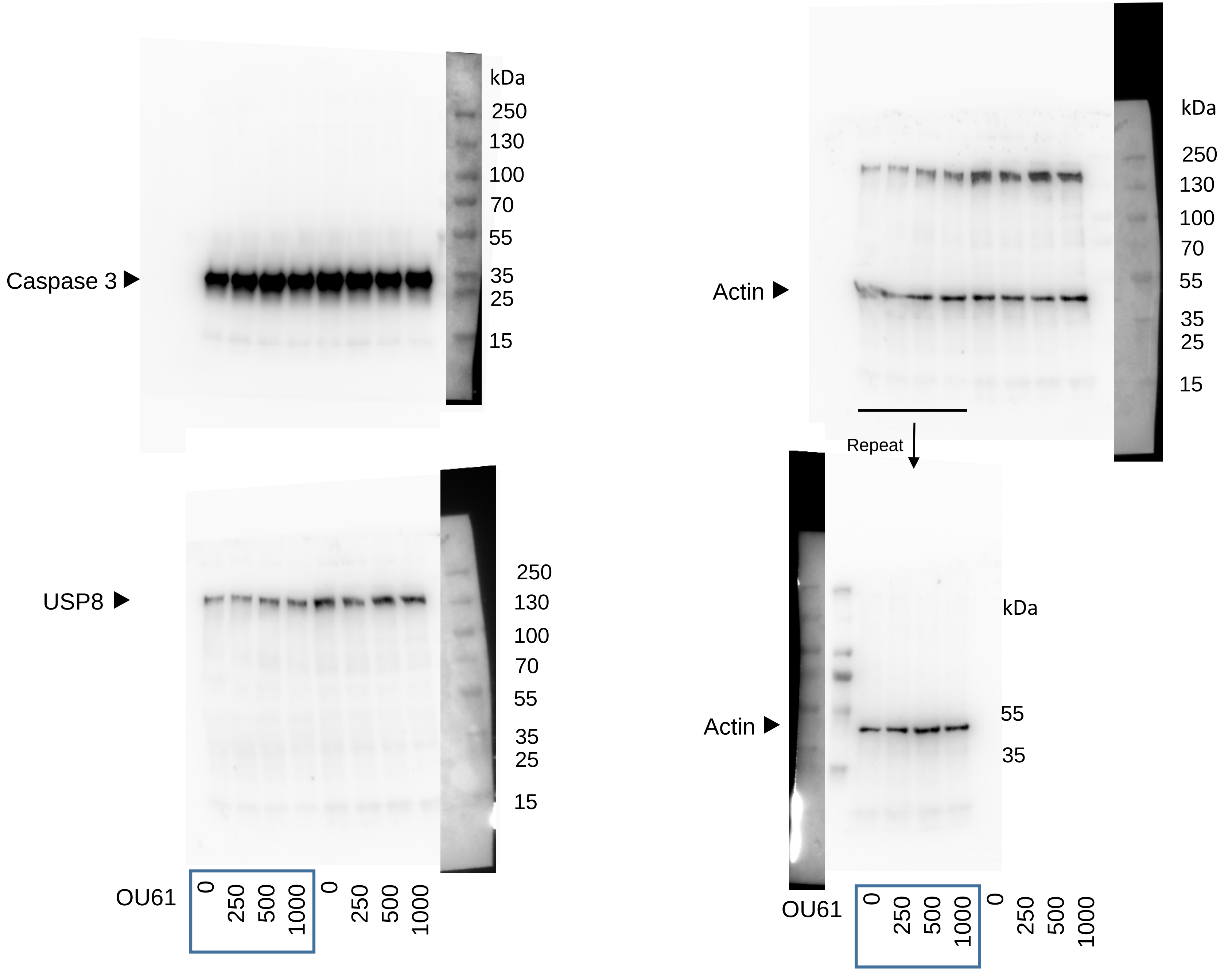

Supplement: Supplementary file 2 [file 41375_2026_3086_MOESM3_ESM.pdf]
